# The anti-CD33 antibody drug conjugate gemtuzumab ozogamicin depletes and functionally resets CD33^+^ myeloid-derived suppressor cells in patients with metastatic cancer: a phase 2 single-arm, open-label trial

**DOI:** 10.1093/jleuko/qiag083

**Published:** 2026-06-18

**Authors:** Carmela De Santo, Aimee Jackson, Francis Mussai, Saly Al-Taei, Neeraj Lal, Thomas Starkey, Livingstone Fultang, Nicola McJannett, Victoria Kunene, Lucinda Billingham, Anna Lawson, Su Lee, Joseph Rogers, Gary Middleton

**Affiliations:** Department of Immunology and Immunotherapy, College of Medicine and Health, University of Birmingham, Edgbaston, Birmingham B15 2TT, United Kingdom; Cancer Research UK Clinical Trials Unit, University of Birmingham, Edgbaston, Birmingham B15 2TT, United Kingdom; Paediatric Oncology, Birmingham Children's Hospital, Birmingham, B4 6NH, United Kingdom; Clinical Immunology Service, University of Birmingham, Edgbaston, Birmingham B15 2TT, United Kingdom; Department of Immunology and Immunotherapy, College of Medicine and Health, University of Birmingham, Edgbaston, Birmingham B15 2TT, United Kingdom; Faculty of Health and Life Sciences, Institute of Systems, Molecular & Integrative Biology, University of Liverpool, Liverpool L69 7ZX, United Kingdom; Cancer and Genomic Sciences, College of Medicine and Health, University of Birmingham, Edgbaston, Birmingham B15 2TT, United Kingdom; Department of Immunology and Immunotherapy, College of Medicine and Health, University of Birmingham, Edgbaston, Birmingham B15 2TT, United Kingdom; Department of Immunology and Immunotherapy, College of Medicine and Health, University of Birmingham, Edgbaston, Birmingham B15 2TT, United Kingdom; University Hospitals Birmingham NHS Foundation Trust, Mindelsohn Way, Birmingham B15 2WB, United Kingdom; Cancer Research UK Clinical Trials Unit, University of Birmingham, Edgbaston, Birmingham B15 2TT, United Kingdom; Cancer Research UK Clinical Trials Unit, University of Birmingham, Edgbaston, Birmingham B15 2TT, United Kingdom; Cancer Research UK Clinical Trials Unit, University of Birmingham, Edgbaston, Birmingham B15 2TT, United Kingdom; Cancer Research UK Clinical Trials Unit, University of Birmingham, Edgbaston, Birmingham B15 2TT, United Kingdom; Department of Immunology and Immunotherapy, College of Medicine and Health, University of Birmingham, Edgbaston, Birmingham B15 2TT, United Kingdom; Cancer Research UK Clinical Trials Unit, University of Birmingham, Edgbaston, Birmingham B15 2TT, United Kingdom; University Hospitals Birmingham NHS Foundation Trust, Mindelsohn Way, Birmingham B15 2WB, United Kingdom; National Institute for Health Research Birmingham Biomedical Research Centre, University of Birmingham, Birmingham B15 2TT, United Kingdom

**Keywords:** CD33, clinical trial, gemtuzumab ozogamicin, myeloid-derived suppressor cells, repolarization

## Abstract

We previously demonstrated that the anti-CD33 antibody drug conjugate gemtuzumab ozogamicin (GO) binds CD33-expressing monocytic myeloid-derived suppressor cells (M-MDSCs), is internalized, and decreases those cells’ viability. Treatment of MDSCs with GO restores T-cell proliferation in co-culture, overcomes M-MDSC suppression of CAR-T cell proliferation, and enhances target-cell killing. Gemtuzumab Ozogamicin Therapy in Hemophagocytic Lymphohistiocytosis or Macrophage Activation Syndrome (GOTHAM) is a phase 2 single-arm clinical trial for which patients were eligible if they had a diagnosis of solid cancer with radiological or clinical evidence of disease progression, or primary or secondary hemophagocytic lymphohistiocytosis, or macrophage activation syndrome disease relapsing or refractory to treatment at enrollment. An initial regimen of 3 mg/m^2^ GO on days 1, 8, and 15 was tested, adjusted to 21-d intervals: days 1, 22, and 43. The primary outcome was the impact of GO therapy on peripheral CD33^+^ myeloid cells. Using 2 schedules of GO, we could not convincingly demonstrate safe feasibility in patients with solid cancer, because of neutropenia. However, GO reproducibly and significantly reduced circulating MDSCs. Importantly, there is consistent preliminary evidence that, upon rebound, the monocyte population of CD33^+^ cells is replaced with nonsuppressive monocytes. These data support the phase 1b dose-escalation testing of GO up to 2 mg/m^2^ in combination with immune checkpoint blockade and other immunotherapies in patients with solid cancer to find a dose that depletes and repolarizes MDSCs without causing undue neutropenia, paving the way to use GO as an immune potentiator in this patient population.

Trial registration: ISRCTN 89158144

## Teaser

In people with cancer, the generation of myeloid-derived suppressor cells (MDSCs) is driven by myeloid cell expansion and conditioning in bone marrow and spleen, and subsequent peripheral pathological activation in blood and tumor. MDSCs are highly immunosuppressive, and mononcytic (M)-MDSCs, the most immunosuppressive MDSC subtype, give rise to tumor-associated macrophages upon migration into tumoral tissue. CD33 is highly expressed on the surface of MDSCs, particularly M-MDSCs. The anti-CD33 antibody drug conjugate gemtuzumab ozogamicin (GO) binds predominantly to M-MDSCs and induces dose-dependent decrease in their viability. We show that in people with metastatic cancer treated with GO, the drug significantly reduces circulating MDSCs and, upon rebound, the monocyte population of CD33^+^ cells is replaced with nonsuppressive monocytes.

## 1. Introduction

Myeloid-derived suppressor cells (MDSCs) are a paradigmatic, immunosuppressive cell population found in the blood and tumors of people with cancer.^1^ MDSC generation is driven by myeloid cell expansion and conditioning in bone marrow and spleen, and subsequent peripheral pathological activation in blood and tumor, giving rise to 2 distinct MDSC populations: polymorphonuclear MDSCs (G-MDSCs) and monocytic MDSCs (M-MDSCs). G-MDSCs preferentially use reactive oxygen species, peroxynitrite, arginase 1, and prostaglandin E_2_ to mediate immune suppression, whereas M- MDSCs principally use nitric oxide, IL-10, TGF-β, and PD-L1. Furthermore, M-MDSCs home to tumors and there differentiate into immunosuppressive tumor-associated macrophages (TAMs), in part related to hypoxia.^2–5^ Macrophages generated from M-MDSCs have higher expression of multiple immunosuppressive factors compared with macrophages generated from classical monocytes and, like their M-MDSC progenitors, suppress T-cell proliferation.^3^

Given their pivotal role immunosuppressive role in cancer, anti-MDSC strategies are an attractive approach to augment anticancer immunity and improve the efficacy of anticancer immunotherapies.^1^ One obvious approach is to deplete MDSCs. However, although some chemotherapies have been reported to reduce MDSC numbers, this approach is nonspecific, and it is difficult in the clinical setting to attribute the MDSC effect directly to the chemotherapy rather than to a reduction in disease burden with reduced stimulus to MDSC induction. A more specific approach that has been highlighted is to target CD33.^1^

MDSCs express the common myeloid marker CD33. We previously demonstrated that tumor stroma is significantly infiltrated by CD33^+^ myeloid cells and that CD33 expression is significantly higher on M-MDSCs compared with G-MDSCs.^6^ Peripheral CD33^+^ MDSCs in patients with cancer are suppressive of T-cell proliferation whereas CD33^+^ cells from healthy donors are not. We demonstrated that the anti-CD33 antibody drug conjugate (ADC) gemtuzumab ozogamicin (GO) binds predominantly with the M-MDSC population, is rapidly internalized, and induces dose-dependent decrease in their viability. Treatment of circulating patient-derived or tumor-polarized MDSCs with GO restores T-cell proliferation in co-culture. GO therapy overcomes CD33^+^ MDSC-mediated suppression of CAR-T cell proliferation and GO-induced MDSC killing significantly enhances target-cell killing. As such, depletion of M-MDSCs using an anti-CD33 ADC such as GO might not only reduce M-MDSC–related immunosuppression but also the accumulation of TAMs derived from them. This clinically translatable approach was highlighted in a prominent expert review on MDSC biology and targeting.^1^

We report here the results of the Gemtuzumab Ozogamicin Therapy in Hemophagocytic Lymphohistiocytosis or Macrophage Activation Syndrome (GOTHAM) trial, a phase 2 trial assessing the impact of GO on peripheral CD33^+^ cell count for the first time in patients with solid cancer. We initially tested the lower-dose fractionated schedule of 3 mg/m^2^ GO on days 1, 4, and 7, as approved by the US Food and Drug Administration (FDA) in 2017 and associated with less early death, hemorrhage, and veno-occlusive disease (VOD) but with equivalent therapeutic effect as a 9 mg/m^2^ for 2 doses 14 d apart.^6,7^ No dose de-escalation of GO was planned in the protocol. Using 2 different schedules of GO at 3 mg/m^2^, we could not convincingly demonstrate safe feasibility in these multiply pretreated patients, because of neutropenia. However, GO reproducibly and significantly reduced circulating MDSCs in patients with cancer, and we present preliminary but consistent evidence that, upon rebound, the monocyte population of CD33^+^ cells is replaced with nonsuppressive monocytes. These data support the ongoing exploration of anti-CD33 therapies as anti-MDSC agents and specifically dose-escalation phase 1b testing of GO to a maximum of 2 mg/m^2^ in combination with immune checkpoint blockade (ICB) and other immunotherapies in patients with solid cancer with the aim of finding a dose that depletes and repolarizes M-MDSCs without causing undue neutropenia at the same time.

## 2. Methods

### 2.1. GOTHAM trial

#### 2.1.1. Design

GOTHAM is a phase 2, single-arm, open-label trial of patients with either relapsed/refractory (R/R) hemophagocytic lymphohistiocytosis (HLH) or macrophage activation syndrome (MAS) (group 1), or R/R solid tumors (group 2) recruiting patients from 2 hospitals in the United Kingdom (clinical trial registration identifiers EudraCT 2020-002428-36 and ISRCTN 89158144). The full protocol is provided in Appendix S1.

#### 2.1.2. Ethics

Ethical approval for the trial protocol (ultimately v6.0, dated 20 July 2023) was gained from the South Central—Hampshire A Research Ethics Committee and the local institutional review board in accordance with national and international guidelines.

#### 2.1.3. Participants

Patients were eligible for trial entry if they were >1 yr old, had a diagnosis of primary or secondary HLH or MAS disease that is R/R to treatment at time of enrollment (group 1) or had histologically confirmed diagnosis of solid cancer with radiological or clinical evidence of disease progression or any subsequent recurrence (group 2). Pediatric safety and preliminary efficacy outcomes using the lower-dose fractionated schedule of 3 mg/m^2^ days 1, 4, and 7 of GO as monotherapy that the FDA approved in 2017 were comparable to those in adults, except for a higher incidence of VOD with the unfractionated regimen. Patients in group 2 were required to have adequate liver function and not have evidence of sinusoidal obstruction syndrome/VOD. Patients who had previous treatment with another CD33-targeting antibody or immunotoxin or hypersensitivity to GO were not eligible.

A negative pregnancy test for female patients of childbearing potential was required within 7 days prior to trial entry. Sexually active patients were only eligible if they agreed to use 2 methods of adequate and appropriate contraception while receiving the trial drug and for 4 (male) or 7 (female) months after treatment discontinuation. All participants and/or parents or legal guardian gave written informed consent. The patient’s sex was self-reported as male or female.

Patient registration into the trial by the treating clinician at the hospital site was by telephone to a central registration service at the Cancer Research UK Clinical Trials Unit (CRCTU) at the University of Birmingham.

#### 2.1.4. Intervention

Participants started trial treatment of 3 mg/m^2^ GO on day 1 after trial registration. The drug was administered as an infusion over 2 h by appropriately qualified and delegated clinical staff at the hospital site. The trial was originally designed with a 7-d interval dosing schedule (days 1, 8, and 15). After 4 participants had been treated on this schedule, it was deemed infeasible and, following a trial management group (TMG) meeting decision, ratified by the independent Trial Steering Committee (TSC) and approved by the UK's competent authority, the Medicines and Healthcare Products Regulatory Agency, the South Central—Hampshire A Research Ethics Committee, and the sponsor (University of Birmingham), all subsequent participants received the same doses of GO but at 21-d intervals on days 1, 22, and 43.

#### 2.1.5. Outcomes

The primary outcome was to assess the activity of GO therapy on CD33^+^ myeloid cells in the blood at days 1, 8, 15, 22, 29, 50, and 57. Secondary outcomes were to assess the effect of GO on overall survival and progression-free survival time, defined as the time from registration to the date of death and progression or death, respectively, as well as assessing the incidence of grades 3 and 4 adverse events according to National Cancer Institute's Common Terminology Criteria for Adverse Events version 4.03.^8^

Protocol-defined exploratory outcomes were to measure the change in blood plasma IL-1/IL-6/TNF-α concentrations as well as the change in CD33^+^ cells in the bone marrow or tissue where available.

#### 2.1.6. Statistical analysis

Because no formal statistical testing was planned for the trial, no sample size justification was made. Instead, a minimum of 10 patients per diagnostic group (R/R HLH or MAS [group 1] or R/R solid tumors [group 2]) was estimated to be feasible and set as the target recruitment.

Analysis was conducted in line with the predefined statistical analysis plan and conducted on an evaluable population, defined as patients who received at least 1 dose of treatment, provide a sample on day 1 of treatment, and received a sample at least 1 other time point. Safety analysis was carried out on the safety population, defined as all patients who received at least 1 dose of trial treatment. The statistical analysis plan is provided in Appendix S2.

Standard descriptive analysis techniques were used to present the data; no formal hypothesis testing took place. No formal interim analysis or stopping rules were specified in the trial design. Analyses were performed using SAS, version 9.4.

#### 2.1.7. Patient and public involvement

A patient and public involvement representative was a member of the independent TSC, in which they played a key role in assessing the trial's conduct, safety, and recruitment, as well as discussing and reviewing proposed amendments to the study. The patient and public involvement representative also was engaged in assisting with the dissemination of results, including production of a lay summary.

### 2.2. Immunohistochemistry analysis of tumor-associated macrophages

#### 2.2.1. Ethics

Ethical approval for specimens collected was gained from West Midlands—Edgbaston Research Ethics Committee ethical approval (reference: 13/WM/0339).

#### 2.2.2. Patient cohorts

Two retrospective paired cohorts were analyzed:

- Cohort A: 16 paired primary colorectal cancer (CRC) tumors and matched liver metastases (LMs), sourced from the Birmingham Human Biomaterials Resource Centre (Birmingham, UK).
- Cohort B: 16 paired primary CRC tumors and matched lung metastases. Primary tumors were processed at hospitals across the West Midlands, UK; lung metastases were sectioned at Heartlands Hospital (Birmingham, UK).

Formalin-fixed, paraffin-embedded (FFPE) blocks were sectioned at 4 µm thickness by Birmingham Tissue Analytics.

#### 2.2.3. Tissue processing and staining

We conducted 4-plex fluorescent staining of FFPE tissues using the Leica BOND RX Fully Automated Research Stainer according to standard sequential Leica staining protocols. Initial antigen retrieval was done using pH 9 Leica Bond Epitope Retrieval 2 solution (Leica, AR9640) for 20 min at 100 °C. Sequential staining was performed with rabbit anti-CD64 (1/800, clone EPR23840-139; Abcam, ab302901), followed by rabbit anti-CD206 (1/800, clone E2L9L; Cell Signaling Technology, 91992), mouse anti-CD163 (1/400, clone 10D6; Leica, NCL-L-CD163), and mouse anti-CD68 (1/50, clone 514H12; Leica, NCL-L-CD68). The markers were paired with Opal 520 (1/150; FP1487001KT), Opal 620 (1/100; FP1495001KT), Opal 690 (1/150; FP1497001KT) and Opal 570 (1/150; FP1488001KT)) all from Akoya Biosciences. Slides were counterstained with spectral DAPI (Akoya Biosciences, FP1490). Prolong Diamond anti-fade mountant (Fisher Scientific, 15205739) was used to preserve fluorescent signal during imaging. Images were acquired at 40× using PhenoImager HT (Akoya Biosciences).

#### 2.2.4. Slide analysis and image processing

Regions of interest were manually selected on digitized whole-slide images: multiplex immunofluorescence staining intensities for macrophage markers (CD68 [pan-macrophage], CD64 [M1], CD163 [M2], and CD206 [M2]) were quantified using QuPath (version 0.4.3). Marker positivity thresholds were established in primary tumor samples (colon) and applied to matched liver or lung metastases.

#### 2.2.5. Data preprocessing and normalization

Single-cell marker expression values (mean intensity per cell) were log2-transformed with an offset of 1 (log2 + 1) to approximate normality. Technical batch effects (due to interhospital sample-processing variability) were adjusted using the ComBat() function from the sva R package (version 3.50.0) to ensure batch correction.

#### 2.2.6. Marker positivity determination

Thresholds for marker positivity were derived using the MetaCyto R package (version 1.24.0) with a fixed seed [set.seed(100)]. Cutoff values were calculated on normalized primary tumor data and applied to metastases as indicated in Appendix S3.

#### 2.2.7. Macrophage-subset quantification

The proportion of marker-positive cells was calculated relative to the total cells per region of interest. Coexpression of CD68^+^CD64^+^ was used to define M1 macrophages and co-expression of CD68^+^CD163^+^ or CD68^+^CD206^+^ for M2; M1:M2 ratios were calculated as the ratio of CD68^+^CD64^+^ (M1) to CD68^+^CD163^+^ (M2) cells.

#### 2.2.8. Statistical analysis

The Wilcoxon signed-rank test was used to analyze paired comparisons (primary tumor vs. metastasis). Unpaired comparisons (liver vs. lung metastases) was analyzed using a Wilcoxon rank-sum test. All analyses were performed in R (version 4.3.1), with statistical significance defined as *P* < 0.05.

### 2.3. GOTHAM laboratory materials and methods

#### 2.3.1. Ethics

All translational work performed as ad hoc exploratory analyses were covered under a separate ethical approval (The role and mechanism of action of myeloid-derived suppressor cells (MDSCs) in malignancies: ethics reference no.: 10/H0501/39; sponsor reference no.: RG_12-181; 26 July 2022, version 7.0).

#### 2.3.2. Isolation of peripheral blood mononuclear cells from whole blood

Peripheral blood mononuclear cells (PBMCs) were isolated from the peripheral blood of patients before receiving the treatment. The blood was first diluted in RPMI medium (Sigma) to 2 times the original volume and layered on top of Lymphoprep (StemCell Technologies). The blood and Lymphoprep mixture were centrifuged for 20 min at 2,000 rpm at room temperature with the brake off. The PBMCs were washed twice with RPMI medium (15 min at 1,500 rpm) and for 10 min at 1,100 rpm. The cells pellet was resuspended in 1 mL of DMSO with 10% fetal calf serum (FCS) and the sample were preserved at −80 °C. Samples were defrosted and CD14^+^CD33^+^ cells enriched from the PBMCs using anti-CD14–coated magnetic beads and magnetic-activated cell sorting (MACS) separation columns (Miltenyi Biotec, Bisley, UK).

#### 2.3.3. Generation of monocyte-derived dendritic cells and macrophages

PBMCs were isolated from healthy donor blood by Lymphoprep gradient centrifugation, following the method described in 2.3.2. Monocytes were positively selected, using anti-CD14 MACS (Miltenyi Biotec) and cultured in RPMI medium with 10% FCS with 100 ng/mL granulocyte macrophage–colony-stimulating factor (GM-CSF) (Peprotech) and 100 ng/mL IL-4 (Peprotech) for 5 d in a 6-well plate. Macrophages were generated by culturing monocytes in RPMI medium and 10% FCS with 100 ng/mL GM-CSF (Peprotech), and 100 ng/mL macrophage-CSF (M-CSF) (Peprotech) for 5 d in a 6-well plate.

#### 2.3.4. In vitro treatment with GO

GM-CSF– and M-CSF–derived macrophages were harvested from the 6-well plates, washed twice in RPMI medium and plated in 200 μL of RPMI medium with 5% human AB serum (Sigma) at a range of concentrations of GO (20 to 0.312 mg/mL) in 96-well flat-bottom plates. Five days later, the cells were washed twice, resuspended in 100 mL of fluorescence-activated cell-sorting (FACS) buffer (1× PBS with 5% FCS) containing propidium iodide (Invitrogen) viability stain measured by CytoFLEX Flow Cytometer (Beckman Coulter). Flow cytometry data were analyzed using FlowJo software (BD Biosciences).

#### 2.3.5. Mixed lymphocyte reaction

T cells were enriched from peripheral blood of healthy donors using a Pan T-cell isolation Kit (Miltenyi Biotec) and labelled with cell tracker carboxyfluorescein succinimidyl ester (CFSE) (ThermoFisher Scientific). T cells (n = 2 × 10^5^) were cultured with allogeneic dendritic cells (5 × 10^4^), and 1 × 10^5^ CD14^+^CD33^+^ cells from patient were added. The cells were plated in 200 μL RPMI medium with 5% human AB serum (Sigma) containing β-mercaptoethanol (0.5 mM) in 96-well flat-bottom plates. Cells were incubated at 37 °C, 5% CO_2_ for 4 d, and the number of CFSE-labelled T cells were measured by flow cytometer (CytoFLEX) and analyzed by FlowJo. Data are expressed as a percentage of T-cell proliferation driven by allogeneic dendritic cells in the presence of patient CD14^+^CD33^+^ cells compared with alloreactive T-cell proliferation in the absence of patient myeloid cells (100%).

The use of allogeneic T cells from healthy donors, rather than autologous patient T cells, was deliberate because T-cell proliferation assays are designed to measure how strongly MDSCs inhibit T-cell activation and expansion. To obtain meaningful, interpretable results, the T cells used in the assay must have normal activation potential; even without MDSCs, patients’ T cells often proliferate poorly. Using autologous T cells, it is not possible to distinguish whether reduced proliferation is due to MDSC activity or baseline T-cell dysfunction.

#### 2.3.6. Immunophenotyping

The CD33 assay used to determine the primary end point was independently developed in the Clinical Immunology Service Laboratory at University of Birmingham/University Hospital Birmingham and was used to analyze the GOTHAM trial samples in the same facility. The Clinical Immunology Service is a UK Accreditation Service–accredited facility complying with the principles of good clinical practice to ensure the reliability and integrity of the data.

Whole blood (50 µL) was incubated with antibodies against the following surface antigens: CD33 (BD Pharmingen), CD14 (BD Horizon), CD15 (BD Biosciences), and CD45 (BD Biosciences) in a Trucount tube (BD Biosciences) to derive absolute counts. To the samples was added 450 µL of red blood cell lysis buffer (BD FACS Lysis Buffer) after the acquisition on a BD FACSCanto II Cytometer, and this was analyzed using FlowJo software. CD3/CD4/CD8 enumeration was carried out using the BD Multitest CE-IVD marked kit for CD45, CD3, CD4, and CD8 cells. Staining was also performed in Trucount tubes according to the following calculation:


$$\begin{array}{rl} \mathrm{Absolute}\;\mathrm{cell}\;\mathrm{count}\;\left(\frac{\mathrm{cells}}{\mu L}\right)\;= & \;\left(\frac{\mathrm{cell}\;\mathrm{count}}{\mathrm{bead}\;\mathrm{count}}\right)\;\\ & \times \;\mathrm{Trucount}\;\mathrm{beads}\;\mathrm{concentration} \end{array}$$

For exploratory outcomes, whole blood from patients was lysed with lysis buffer (Qiagen) and then incubated with CD14, HLA-Dr, CD56, CD19, CD127, CD4, CD25, PD1, TIM3, TIGIT, and LAG3 fluorochrome-conjugated antibodies (BD Biosciences) to phenotype immune cell subtypes. After 20 min at 4 °C, the cells were washed and stained with propidium iodide and measured by flow cytometry on a CytoFLEX. Data were analyzed using FlowJo software.

#### 2.3.7. Cytokines analysis

Plasma from peripheral blood of patients was collected following the blood sedimentation for 10 min. A 1 mL sample of plasma for each time point was frozen at −80 °C. Samples were defrosted and 50 mL used for cytokine quantification using a LegendPlex Kit (BD Biosciences). The samples were measured using CytoFLEX Flow Cytometer and analyzed.

#### 2.3.8. Statistical analysis

Statistical significance was assessed using unpaired, nonparametric Mann-Whitney tests for all analyses except for those pertaining to comparisons of T-cell proliferation, for which a paired Wilcoxon rank test was used. All analyses were performed in GraphPad Prism.

## 3. Results

### 3.1. GOTHAM participants

**Figure 1:**
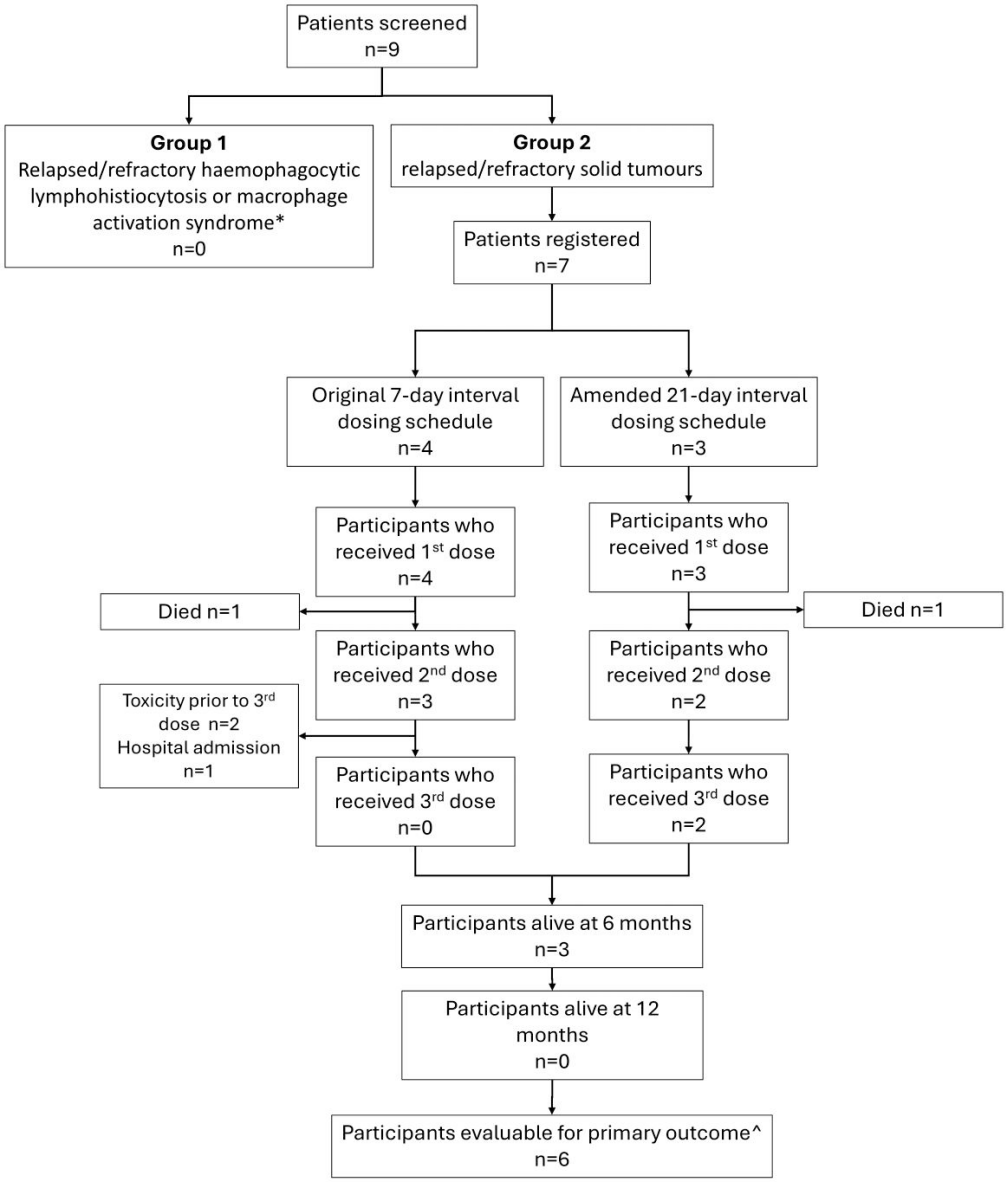
GOTHAM trial participant profile. The number of participants registered, and their treatment received, within the GOTHAM trial. One patient who received the 21-d interval dose schedule did not provide a sample at day 1 of treatment and, therefore, was not deemed evaluable for the efficacy outcomes of the trial.

Seven patients were recruited into GOTHAM between September 2021 and January 2023. All participants recruited had R/R solid tumors and, therefore, were recruited into group 2 (Fig. 1); none had R/R HLH or MAS disease. The other planned cohort (group 1) failed to recruit due to overestimation of patient numbers and a lack of capacity at the centers nationally seeing moderate numbers of these patients. Baseline characteristics of the participants are provided in Table 1. No participants withdrew from the trial, and none were lost to follow-up.

**Table 1:** Baseline participant characteristics.

Characteristic	Total
Sex, no. (%)	
Female	2 (29)
Male	5 (71)
Age, median (IQR) [n], years	65 (55, 67) [7]
Disease type, no. (%)	1 (14)
Localized	
Metastatic	6 (86)
Original tumor type, no. (%)	2 (29)
Adenocarcinoma	2 (29)
Adenocarcinoma of sigmoid colon	1 (14)
Ewing sarcoma	1 (14)
Rectal sigmoid tumor*—*adenocarcinoma	1 (14)
Sigmoid colon	1 (14)
Months since diagnosis, median (IQR) [n]	37.5 (27.7, 40) [7]
Radiologically confirmed relapse	7 (100)
Months since last treatment failure, median (IQR) [n]	3.3 (1.3, 5.3) [7]
Hemoglobin, median (IQR) [n], g/L	97 (91, 136) [7]
White blood cells (×10^9^/L), median (IQR) [n]	10 (7.1, 11.9) [7]
Neutrophils (×10^9^/L), median (IQR) [n]	6.5 (4.1, 8.5) [7]
Platelets (×10^9^/L), median (IQR) [n]	306 (185, 588) [7]
Alanine aminotransferase, median (IQR) [n], U/L	45 (14, 79) [7]
Aspartate aminotransferase, median (IQR) [n], U/L	63.5 (39, 88) [2]
Total bilirubin, median (IQR) [n], µmol/L	8 (7, 17) [7]

IQR, interquartile range.

Although originally designed as an age- and cancer type–agnostic trial (indeed, the first participant treated was a pediatric patient), given the small number of planned participants (a minimum of 10), we decided to reduce any potential heterogeneity in the impact of GO on MDSCs that might arise through inherent differences in MDSC biology or levels between different cancer types. As such, subsequent recruitment focused on a single common cancer type in which M-MDSCs are recognized as important immunosuppressive populations and in which checkpoint blockade has had limited therapeutic success: proficient mismatch repair colorectal cancer (pMMR CRC) with metastatic liver disease. Previously, we described the cross-talk between CRC that produce TGF-β to drive the generation of M-MDSCs and that suppress T-cell function through the production of IL-10.^9^ The liver is the most common site of metastatic involvement in pMMR CRC. Emerging evidence demonstrated that the presence of liver metastases (LM) significantly reduces the efficacy of ICB in melanoma and lung cancer^10,11^ and that metastatic pMMR CRC, classically seen as a nonimmunogenic disease, could respond usefully to ICB in the absence of LM.^12,13^ Single-cell RNA-sequencing data suggest TAMs are important immune subsets mediating immune suppression in CRC LM^10,14–17^ and, as mentioned, immunosuppressive TAMs are derived from M-MDSCs. Furthermore, the expression of CD33 is significantly higher in LM compared with primary CRC.^18^

**Figure 2:**
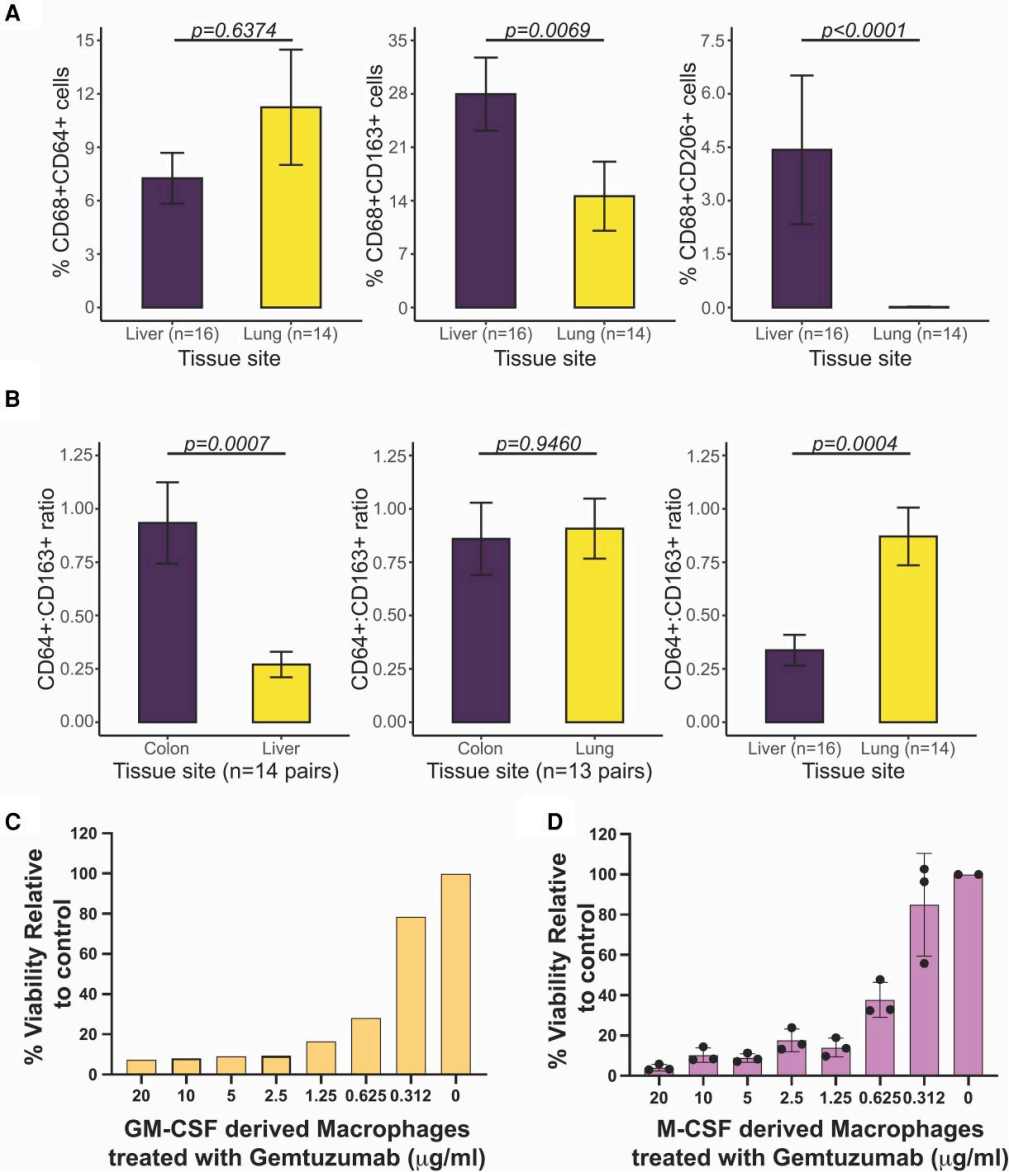
Immunohistochemistry of anti- and pro-tumoral tumor-associated macrophages and cell viability of monocyte-derived macrophages. (A and B) Comparisons are shown for macrophage markers between those identified within LMs and LuMs (A), as well as comparison of CD64/CD163 macrophage marker ratios between primary tumor and metastases (B) as analyzed by flow cytometry. Statistical comparisons were performed using the Wilcoxon signed-rank test. (C and D) Cell viability of monocytes treated with GM-CSF (C) and M-CSF (D) is shown and assessed by flow cytometry following treatment with different concentrations of GO for 5 d. Bars in all panels show means with standard error bars.

To further support this decision, we investigated whether there is a difference in the density of immunosuppressive TAMs in LM compared with primary CRC or CRC metastatic to other non-liver sites. We analyzed by immunohistochemistry the proportions of anti- and pro-tumoral TAMs by phenotyping 16 paired pMMR CRC primaries and LMs and 16 primary and lung metastasis (LuM) pairs using a previously reported panel to sub-type anti-tumoral and pro-tumoral TAMs.^19^ The proportion of both CD163^+^ and CD206^+^ TAMs were significantly lower in LuM compared with LM, and the dearth of CD206^+^ TAMs in LuM is striking (Fig. 2A). The proportion of CD64^+^ TAMs was nonsignificantly greater in LuM compared with LM; however, the CD64^+^:CD163^+^ ratio was significantly higher in LuM compared with LM (Fig. 2B). There was also a significantly lower CD64^+^:CD163^+^ ratio in LM compared with primary CRC tissue but no difference in this ratio between primary tissue and LuM. As expected, TAMs expressed CD33 (Appendix S4a), and we show that GO induces a dose-dependent decrease in the viability of monocyte-derived macrophages (Fig. 2C and 2D).

### 3.2. Feasibility and tolerability of GO

The first 4 trial participants were treated with 3 mg/m^2^ GO on a weekly dosing schedule (days 1, 8, and 15). The FDA identified the MyloFrance trial as an adequately sized and well-designed trial of GO at 3 mg/m^2^ on the day 1, 4, and 7 schedule.^7^ Although mature neutrophils express lower CD33 levels than do monocytes, this is the first time, to our knowledge, that GO has been administered to patients with solid cancer and the TMG felt that giving the total dose over a week as in R/R acute myeloid leukemia (AML) would possibly be too intensive when targeting myeloid cells in patients with non-AML. Therefore, initially, the same total dose was spaced out to 3 doses over 3 wk on a days 1, 8, and d15 schedule. However, this weekly regimen was deemed not to be feasible. The first participant was a pediatric patient with soft-tissue Ewing's sarcoma who had had received multiple lines of prior therapy and exhausted all standard-of-care therapy options. They received GO on days 1 and 8 but the participant rapidly became symptomatic and subsequently received palliative care and died of tumor progression. Subsequently, 3 adult participants with advanced chemorefractory pMMR CRC metastatic to the liver were treated on the days 1, 8, and 15 schedule. The first participant received days 1 and 8 of GO but was admitted with neutropenic sepsis on day 14; they fully recovered after receiving supportive care. The second participant received day 1 GO but was admitted on day 5 with non-neutropenic biliary sepsis due to calculous disease. This settled but, following a second episode of biliary sepsis, the consulting physician removed the patient from trial. The third participant received days 1 and 8 doses of GO. The patient had grade 4 neutropenia on day 14 and was treated with prophylactic G-CSF and did not receive the third dose of GO. Thus, none of the 4 participants treated on the days 1, 8, and 15 schedule received all 3 doses; hence, this schedule was deemed unfeasible in multiply pretreated patients with solid cancer. It is important to note that the administration of G-CSF is associated with the induction of immunosuppressive M-MDSCs and an increased frequency of G-MDSCs.^20^ Thus, any use of G-CSF to offset the potential for serious infection due to GO-induced neutropenia will likely counteract the effect of GO delivered as an anti-MDSC therapy.

Following a TMG meeting decision, ratified by the independent TSC and approved by the competent authorities, ethics board, and sponsor, subsequent participants received the same doses of GO at 21-d intervals on days 1, 22, and 43. It was hoped that this would avoid the cumulative effect of closely spaced GO dosed at 3 mg/m^2^. Both patients with CRC in the initial group who received more than 1 dose of GO developed grade 4 neutropenia after the day 8 dose. Furthermore, this schedule would provid an anti-MDSC effect during the first 3 cycles of ICB given on a 3 weekly schedule or the first 2 cycles given on a 6-weekly schedule, rather than just covering the first cycle. Three patients with pMMR CRC and LM were subsequently treated on this 3-weekly schedule. The first 2 participants treated received all 3 doses and stayed well throughout. However, both patients had cycle 1 grade 4 neutropenia lasting no more than 7 days, and 1 received prophylactic G-CSF. This latter patient also had grade 4 neutropenia during cycle 3. The third participant received day 1 GO but was admitted to the outside referring institution on day 8 and subsequently died at day 14. The primary cause of death was certified as pneumonia secondary to immunosuppression. The nadir neutrophil count was 0.12.

Despite the small numbers of treated patients, it was concluded that modified-schedule fractionated GO at 3 mg/m^2^ is unlikely to be feasible as an immunomodulatory agent that can be routinely and safely used as part of combination immunotherapy regimens in patients with solid cancer. Despite being unable to define a feasible schedule of GO 3 mg/m^2^ in pretreated patients with solid cancer, it was important to determine the impact of GO on the dynamics and biology of MDSCs, given this is the first time the drug has been used for this purpose.

### 3.3. The impact of GO administration on CD33^+^ cells

**Figure 3:**
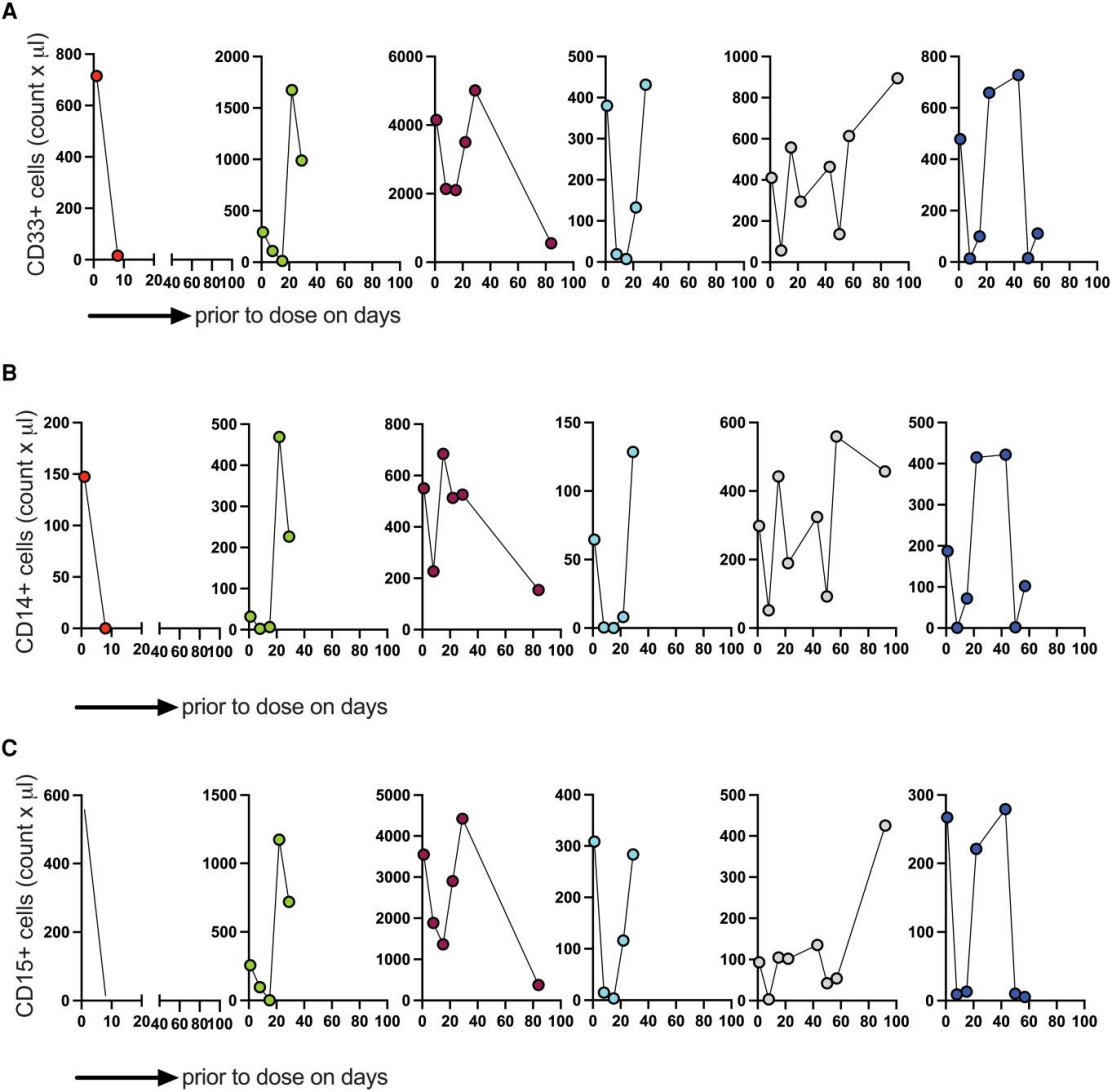
CD33^+^ myeloid cell populations after GO therapy. (A–C) Blood samples collected from participants recruited into the GOTHAM trial at each visit (those collected prior to GO treatment were relevant) analyzed for CD33^+^ cell frequency (A) and the numbers of CD33^+^CD14^+^ cells (B), and CD33^+^CD15^+^ cells (C) using flow cytometry. Each plot represents samples from a single patient; timing of GO administration (in days) is indicated above each plot. Note the prompt and robust drop in CD33^+^ cell count after cycle 1 and cycle 3 of GO given on the 3-weekly schedule in the final 2 plots of each panel. No blood collection was performed the week after GO cycle 2 on this schedule (day 29), hence the apparent lack of reduction after cycle 2.

The predefined primary outcome of the trial was to assess whether GO caused a reduction in circulating CD33^+^ cells. In the 6 trial participants evaluable for this end point (sequential on-treatment blood samples following baseline were not available from the last patient treated), GO administration using both dosing schedules caused a highly reproducible and profound short-lived reduction in the absolute number of circulating CD33^+^ cells (Fig. 3A). Appendix S5a shows the degree of change from baseline (baseline set at 100%) in CD33^+^ cells. The time to reach the lowest value was 7 d in 50% of patients and 14 d in the other 50%, and in all patients, the CD33^+^ cell count subsequently rebounded to values greater than baseline within 2 to 3 wk. Therefore, we show, for the first time, to our knowledge, that GO administration in people with solid cancer causes a prompt, significant, and predictable reduction in circulating nonmalignant CD33^+^ cells. The trajectories of the separate CD14^+^ and CD15^+^ populations were very similar to that of the total CD33^+^ cell population (Fig. 3B and C, respectively). Appendix S5b shows the degree of change from baseline in CD14^+^ cells.

**Figure 4:**
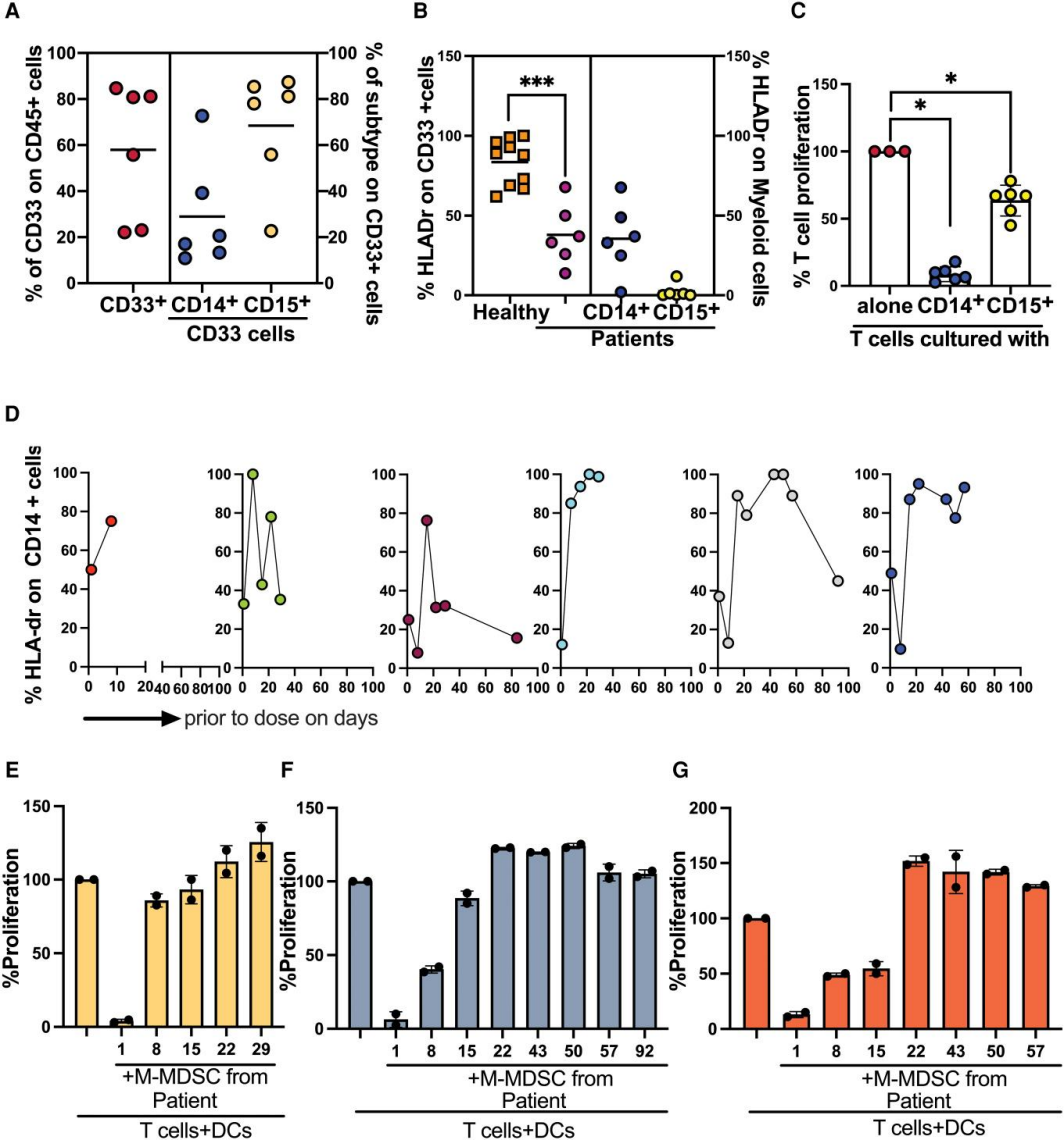
Impact of GO treatment on HLA-DR expression and T-cell proliferation in patients with cancer. (A) Blood samples collected from GOTHAM participants prior to any GO treatment were analyzed for CD33^+^ cell frequency. (B) Low expression of HLA-DR expression was evaluated on these CD33^+^ cells expressing CD14 or CD15 from participants compared with healthy donors. ***P<0.001, as tested using unpaired, non-parametric Mann-Whitney test. (C) T-cell proliferation was also measured after a 4-d co-culture with either CD33^+^CD14^+^ or CD33^+^CD15^+^ cells. The number of T cells were determined using flow cytometry. Activated T cells cultured in RPMI medium with 10% FCS was used as a control. *P<0.05, as tested using unpaired, non-parametric Mann-Whitney test. (D) Blood samples collected from participants recruited into the GOTHAM trial at each visit (prior to GO treatment where relevant) analyzed for HLA-DR expression on CD33^+^CD14^+^ cells; each plot represents samples from a single patient; HLA-DR expression was restored after GO treatment. (E–G) In addition, these CD33^+^CD14^+^ cells lost the capacity to suppress T-cell proliferation; data shown for 3 patients. *P* values correspond to the comparison of the T cells plated with CD14^+^ cells from the samples after the treatment compared with the T cells plated with CD14 sample before the first treatment (day 1 in the graph), showing a complete recovery of T-cell proliferation (by paired Wilcoxon rank test, *P* = 0.0312 [e], *P* = 0.0078 [f], and *P* = 0.0156 [g]). DC, dendritic cells.

We analyzed the phenotype of the monocytic cells on rebound, performing additional ad hoc analyses to explore other parameters of myeloid cell biology and functionality. The expression of HLA-DR was significantly lower on the CD33^+^ cells from the participants enrolled into the trial at baseline compared with the MHC class II expression on CD33^+^ cells from healthy donors (Fig. 4A and B). In line with this, both CD33^+^CD14^+^ and CD33^+^ CD15^+^ populations from the trial participants were significantly suppressive of polyclonal T-cell proliferation, an effect particularly marked with the CD14^+^ CD33^+^ M-MDSC populations (Fig. 4C).

Interestingly, the CD33^+^CD14^+^ cells at rebound showed a marked and maintained increase in the number of cells expressing HLA-DR, suggesting replacement with nonsuppressive monocytes after GO therapy (Fig. 4D and Appendix S4b and c). Importantly, in line with this phenotypic shift in CD33^+^ cells at rebound, there was a sustained and complete loss of monocyte suppression of activated CD4^+^ T-cell proliferation (Fig. 4E–G). Thus, GO significantly reduced M-MDSCs and G-MDSCs in patients with pMMR CRC with LM and, on CD33^+^ cell reset, the CD14^+^ cells were both phenotypically and functionally no longer immune suppressive. This loss of monocyte-induced immune suppression was maintained throughout the entire duration of treatment.

**Figure 5:**
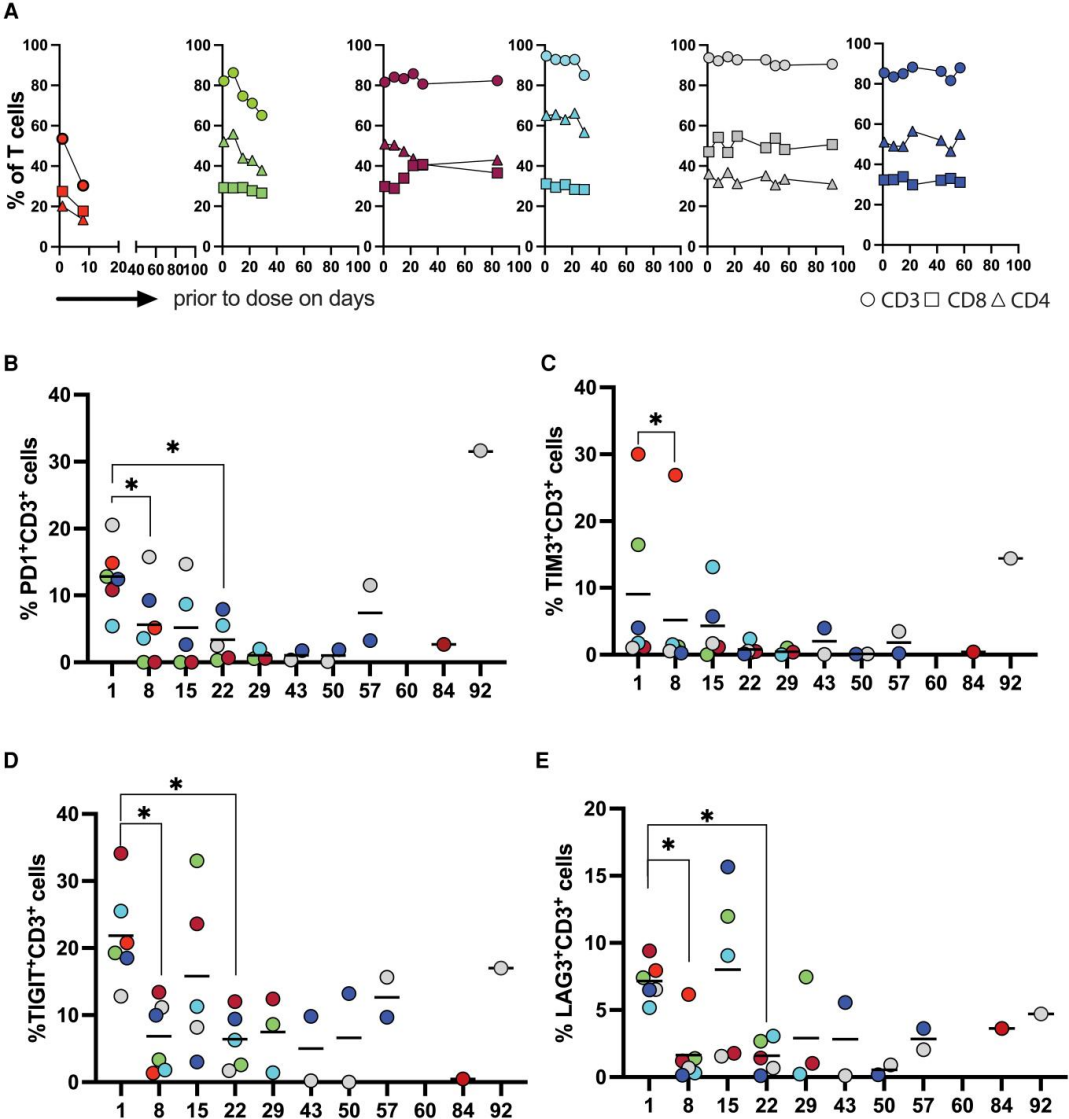
Impact of GO treatment on T-cell subsets in patients with cancer. (A) Blood samples collected from participants recruited into the GOTHAM trial at each visit (samples collected prior to GO treatment were relevant) analyzed for T-cell frequency; each plot represents samples from a single patient. The percentage of CD3^+^, CD4^+^. and CD8^+^ cells were not affected by GO treatment. (B–E) The measurement of markers of exhaustion showed a significant reduction of expression of PD1 (B), TIM3 (C), TIGIT (D), and LAG3 (E) on T cells after GO treatment. Individual patients' samples are identified by a distinct color. Bars indicate means. *P = 0.03 by paired Wilcoxon rank test.

**Figure 6:**
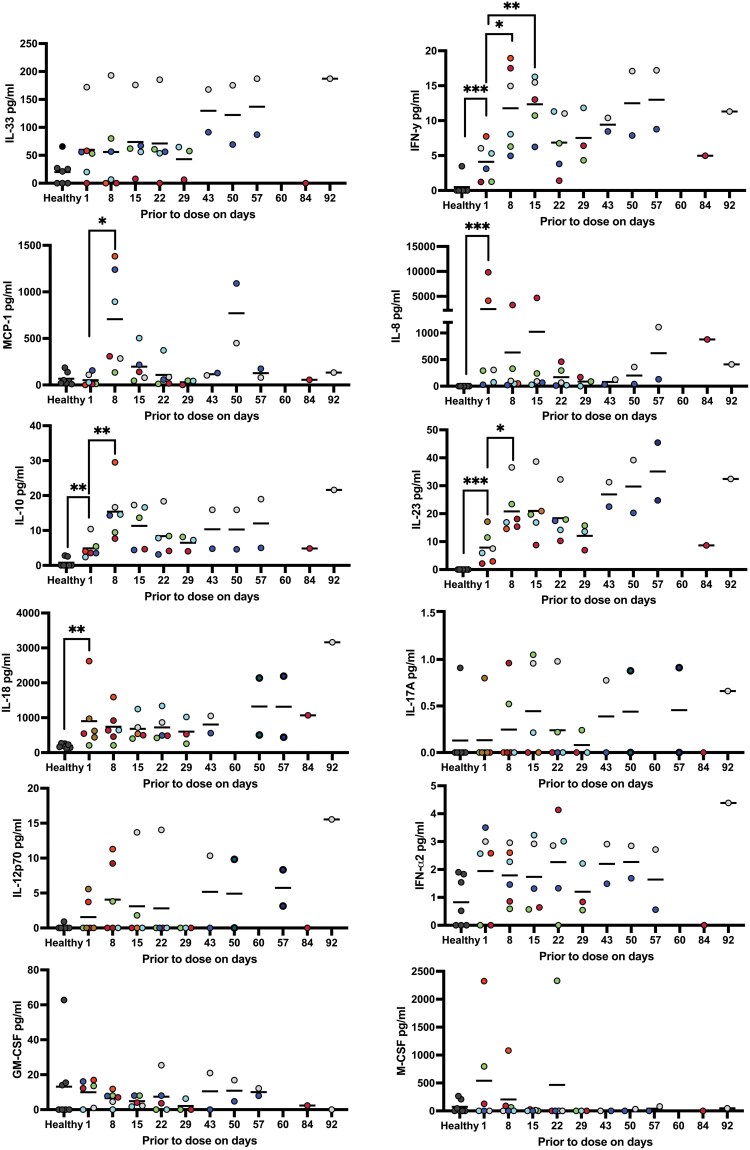
Cytokine analysis in plasma of patients with cancer prior to GO treatment. Plasma samples from patients with cancer were analyzed for cytokine levels using the LegendPlex flow cytometry kit. Individual patients' samples are identified by a distinct color. Bars show means. *P < 0.05, **P < 0.01, ***P < 0.001, as tested using unpaired, nonparametric Mann-Whitney tests.

In additional ad hoc analyses, there was no significant impact of GO administration on the percentage of CD3^+^, CD4^+^, or CD8^+^ T cells (Fig. 5A), or on the percentage of B cells, natural killer cells, and regulatory T cells (Appendix S6), confirming the specific monocyte-myeloid targeting of GO. However, there was a significant and maintained reduction of PD-1^+^, TIM3^+^, TIGIT^+^, and LAG3^+^ expression on CD3^+^ T cells (Fig. 5B–E). Finally, GO administration was associated with a significant increase in serum levels of IL-23, IL-10, MCP-1, and IFN-γ, all of which were significantly higher at baseline than in healthy donors (Fig. 6).

### 3.4. Other secondary trial outcomes

**Figure 7:**
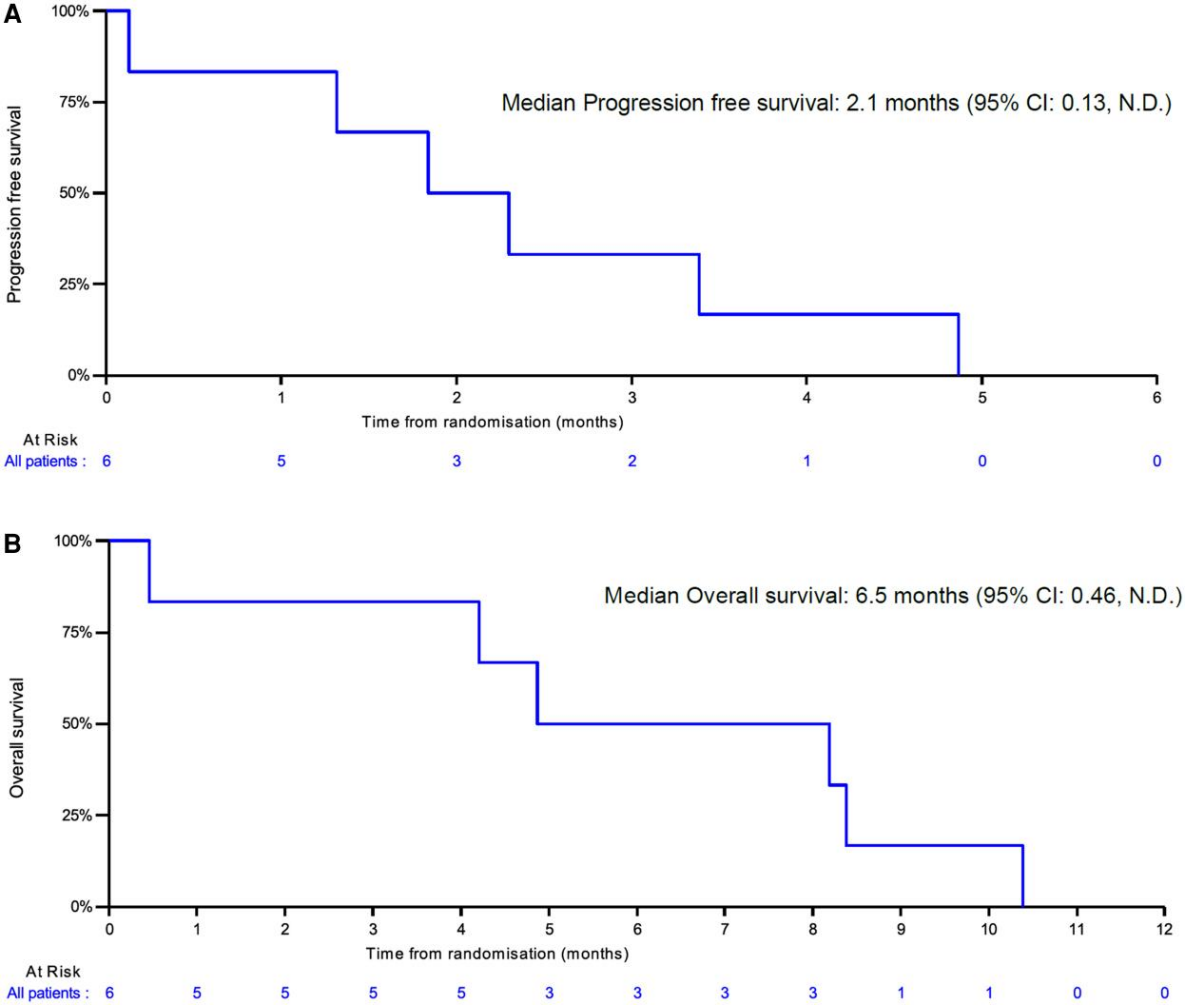
Progression-free and overall survival of participants in the GOTHAM trial. (A) Progression-free survival time defined as the time from the date of entry into the trial to the date of disease progression is shown. Patients lost to follow-up were censored accordingly at date last seen. (B) Overall survival time as defined as the time from the date of entry into the trial to the date of death is shown. Patients lost to follow-up were censored accordingly at date last seen.

Median progression-free and overall survival for evaluable participants were 2.1 mo (95% CI, 0.13 to not defined) and 6.5 mo (95% CI, 0.46 to not defined), respectively (Fig. 7). The observed progression-free survival underlies the criticality of combining GO with other therapies aimed at enhancing the immune attack against cancer, including ICB. All 7 patients died within 11 mo from trial entry, 5 due to disease progression and 2 due to pneumonia. On average, trial participants experienced 2.8 adverse events each while on the trial (Appendix S7). Five serious adverse events were reported in 3 patients, this included 3 events of abdominal pain, 1 event of febrile neutropenia, and 1 event of pneumonia (Appendix S7).

## 4. Discussion

The GOTHAM trial achieved its primary end point; GO is highly effective at depleting CD33^+^ MDSCs in the blood of patients with solid cancer. Critically, after rebound of CD33^+^ cells, CD14^+^ cells are replaced by monocytes that are almost uniformly DR^+^, consistent phenotypically with nonsuppressive classical monocytes, unlike highly suppressive HLA-DR^negative/low^ CD14^+^ cells. We provide preliminary but consistent data showing that the suppressive monocytes are replaced with monocytes that are functionally no longer suppressive of activated T cells. However, fractionated GO at 3 mg/m^2^ on either of the schedules tested in this trial is unlikely to be feasible as a drug that can be routinely and safely used as part of combination immunotherapy regimens in patients with solid cancer. When using any adjunctive therapy alongside standard anticancer immunotherapeutic approaches, a high incidence of grade 4 neutropenia and infection would be considered an unacceptable added risk by most solid cancer oncologists. Furthermore, the use of such an agent would be precluded as part of any combination containing cytotoxic agents such as chemo-immunotherapy or ICB combinations with other ADCs.

There are significant limitations of the study. First, only 7 patients were treated. We originally planned this as a proof-of-principle pilot study in 2 cohorts enrolling a minimum of 10 participants in each cohort. The failure to define a schedule that did not result in significant neutropenia using the protocolized dose of 3 mg/m^2^ was the cause of the limitation of recruitment in the cohort with solid cancer. Additional doses were not explored because dose de-escalation was not per-protocol planned. Although we report on only 7 patients, we clearly demonstrate proof of principle that GO reduces CD33^+^ count and preliminary but consistent data that GO therapy results in replacement of suppressive monocytes with nonsuppressive monocytes. A second major limitation is the lack of pharmacodynamic paired tissue biopsy specimens from liver lesions to assess changes in TAM density and phenotype with GO therapy, but the rationale of this study was to provide proof of principle that GO reduced circulating CD33^+^ MDSCs in patients with solid cancer to support subsequent combination immunotherapy trials with GO in which changes in microenvironmental biology might be more appropriately explored. However, we do show that GO administration is associated with a significant increase in serum levels of IL-23, IL-10, MCP-1, and IFN-γ. MCP-1 (CCL2) is a key chemokine responsible for mediating macrophage recruitment to tumor sites via the CCR2 receptor. Finally, ethnicity data were not collected during GOTHAM, which was a limitation of the trial design and will need to be rectified in future trials.

In conclusion, we show in this study that this anti-CD33 ADC causes a profound reversible reduction in peripheral nonmalignant CD33^+^ cells (both M-MDSCs and G-MDSCs), and there is a functional reset of the monocyte population, which is replaced by CD33^+^ cells that no longer are phenotypically or functionally immunosuppressive. Although we are unable to recommend the 3 mg/m^2^ dose as approved by the FDA in R/R AML, the translational data support continued exploration of GO, and other anti-CD33 therapies, as an anti-MDSC agent. The data support a phase 1b dose-escalation trial of low-dose GO delivered 3-weekly to cover the initial cycles of ICB plus GO with dosing to a maximum dose of GO of 2 mg/m^2^. The aim of this trial would be to define a dose of GO that depletes and resets monocytes without significantly depleting mature neutrophils that express lower levels of surface CD33. As mentioned in the FDA Oncologic Drugs Advisory Committee re-approval of GO,^7^ the original pharmacodynamic analyses of GO showed that GO binding to CD33 is saturated at doses above 2 mg/m^2^. The focus on safely targeting M-MDSCs is based on the cells’ highly potent immunosuppressivity,^2,21^ their intratumoral conversion to TAMs,^3^ and the finding that baseline M-MDSCs, rather than G-MDSCs, are robust predictive biomarkers for outcome with ICB across cancer types, ICB therapeutic, sample size, and cutoff criteria.^22^

## Supplementary Material

qiag083_Supplementary_Data

## Data Availability

Participant data and the associated supporting documentation will be available within 6 months after the publication of this article. Details of our data request process are available on the CRCTU website. Only scientifically sound proposals from appropriately qualified research groups will be considered for data sharing. The decision to release data will be made by the CRCTU Director's Committee, who will consider the scientific validity of the request, the qualifications and resources of the research group, the views of the chief investigator and the trial steering committee, consent arrangements, the practicality of anonymizing the requested data, and contractual obligations. A data-sharing agreement will cover the terms and conditions of the release of trial data and will include publication requirements, authorship, acknowledgements, and obligations for the responsible use of data. An anonymized encrypted dataset will be transferred directly using a secure method and in accordance with the University of Birmingham's information technology guidance on encryption of datasets.
