# Supplementary material for: The anti-CD33 antibody drug conjugate gemtuzumab ozogamicin depletes and functionally resets CD33+ myeloid-derived suppressor cells in patients with metastatic cancer: a phase 2 single-arm, open-label trial
Source: J Leukoc Biol. 2026 Jun 18;118(7):qiag083. doi: 10.1093/jleuko/qiag083 (PMC13346843; doi:10.1093/jleuko/qiag083)
Supplement: qiag083_Supplementary_Data [file qiag083_supplementary_data.pdf]

Appendix 1. GOTHAM trial protocol

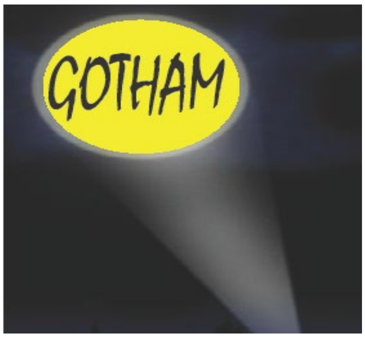

GOTHAM

A phase II trial to assess the activity of Gemtuzumab Ozogamicin Therapy in HAemophagocytic lymphohistiocytosis (HLH) or Macrophage activation syndrome (MAS) or relapsed/refractory solid tumours

Protocol version 6.0

20th July 2023

Sponsor:	University of Birmingham
Sponsor Protocol Number:	RG_19-271
CAS Number:	HC2001
EudraCT Number:	2020-002428-36
ISRCTN Reference Number:	ISRCTN89158144

CANCER RESEARCH UK | BIRMINGHAM CANCER RESEARCH UK CLINICAL TRIALS UNIT | UNIVERSITY OF BIRMINGHAM

TRIAL MANAGEMENT GROUP

Chief Investigator	
Prof. Gary Middleton	Professor of Medical Oncology, , School of Cancer Sciences, University of Birmingham, Edgbaston, Birmingham, B15 2TT<br>☎ 0121 371 2000<br>✉ G.Middleton@bham.ac.uk
**Co-Investigators**	
Professor Pamela Kearns	Director of Cancer Research UK Clinical Trials Unit (CRCTU), University of Birmingham
Dr Carmela De Santo	CRUK New Investigator Fellow, Institute of Immunology and Immunotherapy, University of Birmingham
Professor Lucinda Billingham	Professor of Biostatistics, CRCTU, University of Birmingham

TRIAL SPONSOR

Sponsor	
University of Birmingham, Edgbaston, Birmingham, B15 2TT	
**Coordinating Centre**	
CRCTU, University of Birmingham	
Trial Management Team Leader	Mrs Anna Lawson
Trial Coordinator	Dr Su Lee
Trial Statistician	Mr Amit Patel
Contact Details	Children's Cancer Trials Team (CCTT)<br>Cancer Research UK Clinical Trials Unit (CRCTU)<br>Institute of Cancer and Genomic Sciences, University of Birmingham<br>B15 2TT<br>☎ +44 (0) 121 415 1060<br>✉ GOTHAM@trials.bham.ac.uk<br>🖷 +44 (0) 121 414 9520
Registration	https://www.cancertrials.bham.ac.uk/GOTHAMLive<br>In case of any problems with online registration, please use the contact details above.
Serious Adverse Event (SAE) Reporting	✉ reg@trials.bham.ac.uk
**Laboratory contact**	
Dr Carmela De Santo	Institute of Immunology and Immunotherapy<br>University of Birmingham<br>Birmingham<br>B15 2TT

	☏ +44 (0) 121 414 7047<br>✉ c.desanto@bham.ac.uk

CLINICAL COORDINATORS CONTACT DETAILS

Prof. Gary Middleton	Professor of Medical Oncology, School of Cancer Sciences, University of Birmingham, Edgbaston, Birmingham, B15 2TT<br>☏ 0121 371 2000<br>✉ G.Middleton@bham.ac.uk

SIGNATURE PAGE

GOTHAM Trial Protocol v6.0, 20th July 2023

This protocol has been approved by:

**Name:**	Prof. Gary Middleton	**Trial Role:**	Chief Investigator
**Signature:**	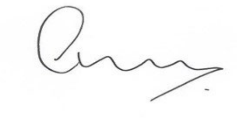	**Date:**	13-Jul-2023

This protocol describes the GOTHAM trial and provides information about procedures for patients taking part in the GOTHAM trial. The protocol should not be used as a guide for treatment of patients not taking part in the GOTHAM trial.

AMENDMENTS

The following amendments and/or administrative changes have been made to this protocol since the implementation of the first approved version

Amendment number	Date of amendment	Protocol version number	Type of amendment	Summary of amendment
1	9-Feb-2021	3.0	Substantial	IMP now provided by Pfizer Ltd instead of from local hospital.
2	16-Mar-2022	4.0	Substantial	Change of dosing schedule
4	30-Sep-2022	5.0	Substantial	Change of Chief Investigator
5	20-Jul-2023	6.0	Substantial	Addition of Day 29 blood sample

TRIAL SYNOPSIS

Title

GOTHAM: A phase II trial to assess the activity of Gemtuzumab Ozogamicin Therapy in HAemophagocytic lymphohistiocytosis (HLH) or Macrophage activation syndrome (MAS) or relapsed/refractory (R/R) solid tumours

Trial Design

GOTHAM is a single arm, open-label, phase II trial in patients with R/R HLH or MAS (Group 1) or patients with R/R solid tumours (Group 2).

Primary Objective

- To assess the activity of gemtuzumab ozogamicin 3 mg/m^2/dose given on Days 1, 22, 43 by measuring the change in CD33+ myeloid cells in the blood in 2 parallel groups.

Secondary Objective

- To assess the effect of gemtuzumab ozogamicin on overall survival time (OS) and progression free survival time (PFS) (Group 2 only).
- To assess the feasibility of delivering gemtuzumab ozogamicin

Exploratory Objectives

- To assess the change in IL-1/IL-6/ TNF-α in the plasma
- To assess the change of CD33+ cells in the bone marrow/ tumour tissue (as available)

Outcome Measures

Primary Outcome Measure

- CD33+ cell count in the blood samples of patients collected at the pre-specified time points Days 1, 8, 15, 22, 29, 43, 50 and 57

Secondary Outcome Measures

- OS time
- PFS time (Group 2 only)
- Incidence of grade 3 and 4 adverse events

Exploratory Outcome Measures

- Change in IL-1/IL-6/ TNF-α in the plasma
- Change of CD33+ cells in the bone marrow/ tumour tissue (as available)

Patient Population

Group 1: Patients with R/R HLH or MAS

Group 2: Patients with R/R solid tumours

Sample Size

A total of 20 evaluable patients: 10 patients per group

Main Inclusion and Exclusion Criteria

Inclusion Criteria

- Aged >1 year old at the time of trial entry
- Diagnosis of primary or secondary HLH or MAS disease that is relapsing/refractory to treatment at time of enrolment (Group 1)
  OR
  Histologically confirmed diagnosis of solid cancer with radiological or clinical evidence of disease progression (during or after completion of at least one previous treatment) or any subsequent recurrence (biopsy at relapse is not mandated) (Group 2)

Note: patients who meet the inclusion criteria for both groups 1 and 2 should be entered into group 1

- Group 2 only – must have adequate liver function:
  - Total bilirubin ≤ x2 upper limit of normal (ULN)
  - Aspartate aminotransferase *(*AST) and/or alanine aminotransferase (ALT) ≤ 2.5 x ULN
- Documented negative pregnancy test for female patients of childbearing potential within 7 days prior to trial entry
- Sexually active patients must agree to use 2 methods of adequate and appropriate contraception while on trial drug and for 4 months (male) and 7 months (female) following treatment discontinuation[1]
- Written informed consent given by patient and/or parents/legal guardian

Exclusion Criteria

- Evidence of sinusoidal obstruction syndrome (SOS) / veno-occlusive disease (VOD)
- Previous treatment with another CD33 targeting antibody or immunotoxin
- Hypersensitivity to Gemtuzumab Ozogamicin or to any of the excipients
- Pregnant or lactating female

[1] Patients with reproductive potential must agree to use two adequate methods of birth control during the period of therapy. Men should be advised not to father a child for 4 months after receiving the last dose of trial treatment. Women of childbearing potential should be advised to use effective contraception to avoid pregnancy for 7 months after the last dose of trial treatment. Effective contraceptive methods include implants, injectables, combined oral contraceptives, intrauterine device (IUD or coil), bilateral tubal occlusion, true sexual abstinence or vasectomised partner, progestogen-only oral hormonal contraception where inhibition of ovulation is not the primary mode of action, male or female condom with or without spermicide and cap, diaphragm or sponge with spermicide. Sperm or egg preservation should be offered as per standard practice to patients at risk of irreversible infertility, where appropriate. Sexual abstinence must be in line with the preferred and usual lifestyle of the patient. Periodic abstinence (e.g. calendar, ovulation, symptothermal, post-ovulation methods), declaration of abstinence for the duration of the trial, and withdrawal are not acceptable methods of contraception.

Trial Duration

The trial will recruit for approximately 2 years, and all patients will be followed up for a minimum of 1 year.

Trials Office Contact Details

GOTHAM Trial Coordinator
Children's Cancer Trials Team (CCTT)
Cancer Research UK Clinical Trials Unit (CRCTU)
Institute of Cancer and Genomic Sciences
Vincent Drive
University of Birmingham
Edgbaston
Birmingham
B15 2TT

Trial Schema

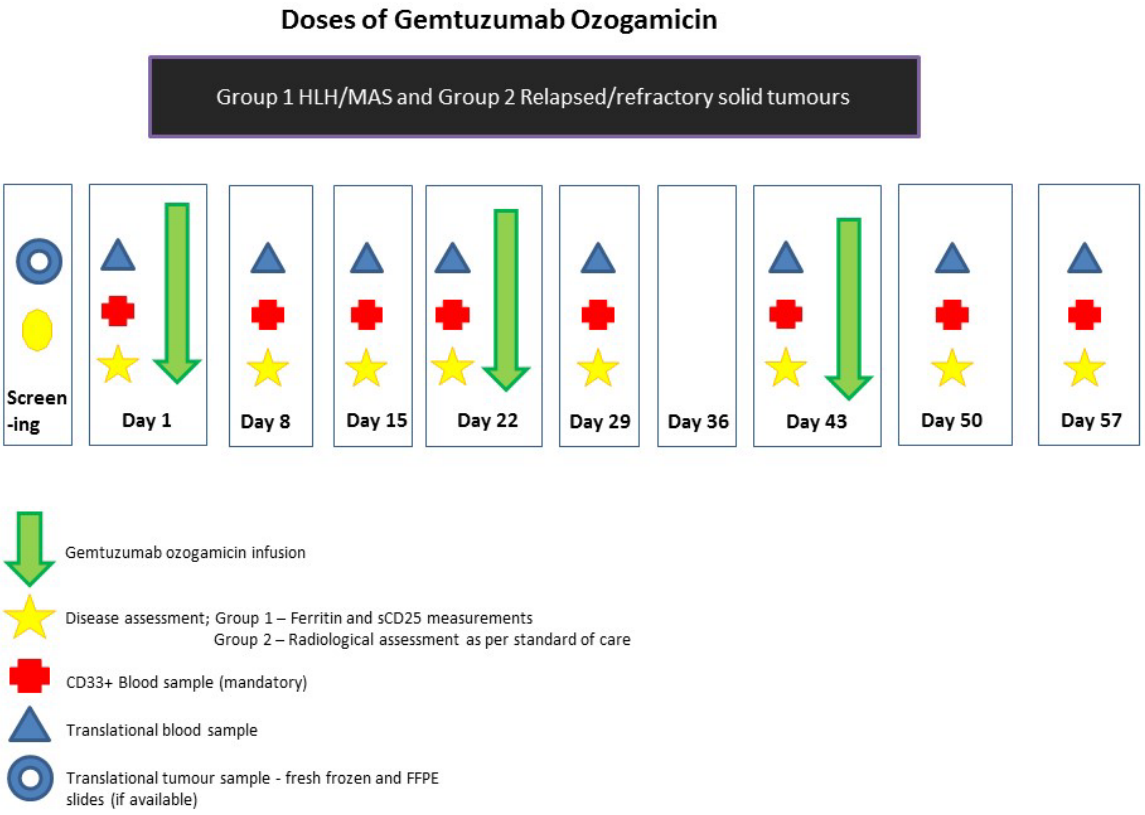

Figure 1: Trial Schema

Schedule of Events

Protocol activity	Screening [a]	Prior to first dose Day 1	Day 8 and 15	Prior to each subsequent dose (Days 22 and 43)	Days 29, 50 and 57 (from first dose)	Follow-up visits	At relapse or progression
		Should be performed on day of dose		Should be performed on day of dose			
**Informed consent/assent**	✓					As per standard of care	
**History/demographics**	✓						
**Physical examination[b]**	✓	✓	✓	✓	✓		✓
**Height**		✓					
**Weight**	✓	✓		✓			
**Vital signs (blood pressure and heart rate, temperature) [c]**	✓	During Infusion		During infusion			
**Premedication**		✓		✓			
**Investigations**							
**Full blood count [d]**	✓	✓	✓	✓	✓	As per standard of care	
**Biochemistry [e]**	✓	✓	✓	✓	✓		
**Pregnancy test [f]**	✓	Monthly during treatment and up to 30 days after				As per standard of care	
**Disease assessment**							
**Group 1** – **Ferritin**	✓	✓	✓	✓	✓	As per standard of care	
**Group 1** – **sCD25**	✓	✓	✓	✓	✓		
**Group 2** – **Radiological disease assessment**		As per standard care					
**Sample collection**							
**Whole blood for CD33+ analysis [g]**		✓	✓	✓	✓		✓
**Whole blood for translational research**		✓	✓	✓	✓		✓
**Fresh frozen tissue from bone marrow aspirate (group 1) or biopsy (group 2) [h]**	✓ (if available)						✓ (if available)
**Paraffin embedded tumour tissue (group 2) [i]**	✓ (if available)						✓ (if available)
**Miscellaneous**							
**Adverse event reporting**		Assess throughout trial					
**Concomitant medications**		Assess throughout trial					

a. Screening assessments are to be conducted within 1 week prior to start of treatment (except consent)
b. Including monitoring for signs of infection
c. Observations required during infusion if clinically indicated

d. Full blood count to include: haemoglobin (Hb), white blood cells (WBC), neutrophils and platelets
e. Biochemistry to include: urea, electrolytes, creatinine, total bilirubin, alkaline phosphate (ALP), AST or ALT (all patients) and for group 1 only: ferritin, sCD25, clotting and triglycerides. For patients who develop abnormal liver tests, more frequent monitoring of liver tests and clinical signs and symptoms of hepatotoxicity is recommended
f. Female patients of childbearing potential
g. Sample collection mandatory and required for the primary outcome measure analysis for the trial
h. Flash frozen tissue if available from routine, clinically indicated biopsy (group 2) or bone marrow aspirate/trephine at diagnosis (group 1) or relapse/progression. In the event of further routine bone marrow aspirates/trephines or tumour biopsies required during the trial, tissue samples are requested
i. Approximately 10 slides from paraffin fixed tissue block required where biopsy has been performed as part of standard of care – see GOTHAM Laboratory Manual

ABBREVIATIONS

ADR	Adverse Drug reaction
AE	Adverse event
ALP	Alkaline phosphatase
ALT	Alanine aminotransferase
AML	Acute myeloid leukemia
AST	Aspartate aminotransferase
BITEs	Bi-specific T cell engager antibodies
BSA	Body surface area
CAR-T	Chimeric-Antigen Receptor T
CD	Cluster of differentiation
CRCTU	Cancer Research UK Clinical Trials Unit
CRF	Case Report Form
CT	Computerised tomography
eRDC	Remote Data Entry Capture system
FDA	Food and Drug Administration
GCP	Good Clinical Practice
GCSF	Granulocyte colony stimulating factor
GM-CSF	Granulocyte/Macrophage Colony Stimulating Factor
GO	Gemtuzumab Ozogamicin
GP	General Practitioner
HCT	Hematopoietic cell transplantation
HLH	Haemophagocytic Lymphohistiocytosis
HSCT	Hematopoietic stem cell transplantation
ICF	Informed Consent Form
IL	Interleukin
IMP	Investigational medicinal product
ISF	Investigator Site File
ITIM	Immunoreceptor tyrosine-based inhibitory motif
IUD	Intrauterine device
MAS	Macrophage activation syndrome
MDSC	Myeloid derived suppressor cells
MHC	Major histocompatibility complex
MHRA	Medicines and Healthcare products Regulatory Agency
NK	Natural Killer
OS	Overall survival time and
PFS	Progression free survival time
PIS	Parent/Patient Information Sheets
PK	Pharmacokinetics
R/R	Relapsed/refractory
R2PD	Recommended phase 2 dose
REC	Research Ethics Committee
SAE	Serious adverse event
SIGLEC	Sialic acid-binding immunoglobulin-type lectins
SmPC	Summary of product characteristics
SOS	Sinusoidal obstructive syndrome
SSDL	Site Signature and Delegation Log

SUSAR	Suspected unexpected serious adverse reaction
TAM	Tumour associated macrophages
TEAE	Treatment emergent adverse events
TLS	Tumour Lysis Syndrome
TMG	Trial Management Group
TSC	Trial Steering Committee
VOD	Veno-occlusive disease

Table of contents

Trial Management Group **2**
Trial sponsor **2**
Clinical coordinators contact details **3**
signature page **4**
AMENDMENTS **5**
Amendment number 5
Date of amendment 5
Protocol version number 5
Type of amendment 5
Summary of amendment 5
Trial Synopsis **6**
Title 6
Trial Design 6
Outcome Measures 6
Patient Population 6
Sample Size 6
Main Inclusion and Exclusion Criteria 7
Trial Duration 7
Trials Office Contact Details 7
Trial Schema 8
Schedule of Events 9
1. Background and Rationale **16**
1.1 Lay Summary 16
1.2 Background 16
1.2.1 Preclinical background 17
1.2.2 Preclinical rationale for gemtuzumab ozogamicin 18
1.2.3 Clinical background 18
1.3 Trial Rationale 19
1.3.1 Justification for patient population 19
1.3.2 Justification for design 20
2. Aims, Objectives and Outcome Measures **20**
2.1 Aims and Objectives 20
2.2 Outcome Measures 21
3. Trial Design **21**
4. Eligibility **22**
4.1 Inclusion Criteria 22
4.2 Exclusion Criteria 22
5. Screening and Consent **22**
5.1 Screening 22
5.2 Informed Consent 23
5.3 Contraception 24
6. Trial Entry **24**
7. Treatment Details **25**
7.1 Investigational Medicinal Products 25
7.2 Treatment Schedule 25
7.3 Pre-Medication 25
7.4 Warnings for use 25

7.5 Assessments 26
7.6 Sample Collection 27
7.6.1 Blood samples for CD33+ analysis 27
7.6.2 Tumour tissue and blood sample collection for translational research 27
7.7 Dose Modifications 28
7.7.1 SOS/VOD 28
7.7.2 Haematopoietic stem cell transplant (HSCT), moderate/severe liver impairment and SOS/VOD 29
7.8 Treatment Compliance 29
7.9 Supportive Treatment 29
7.9.1 Nausea and Vomiting 29
7.9.2 Fever and Neutropenia 30
7.9.3 Thrombocytopenia, decreased haemoglobin or infection 30
7.9.4 Diarrhoea 30
7.9.5 Allergic reaction 30
7.9.6 Blood products 30
7.9.7 GCSF / GM-CSF 30
7.9.8 Tumour lysis syndrome 30
7.9.9 SOS/VOD 30
7.10 Concomitant Medication 30
7.11 Patient Follow Up 31
8. Treatment Discontinuation and Patient Withdrawal 31
8.1 Discontinuation of gemtuzumab ozogamicin 31
8.2 Withdrawal of consent 31
9. Adverse Event Reporting 31
9.1 Reporting Requirements 32
9.1.1 Adverse Events (AE) 32
9.1.2 Serious Adverse Advents 32
9.1.3 Reporting period 32
9.2 Reporting Procedure 33
9.2.1 Site 33
9.2.2 Trials Office 34
9.2.3 Reporting to the Competent Authority and main Research Ethics Committee 34
9.2.4 Investigators 34
9.2.5 Trial Steering Committee 34
10. Data Handling and Record Keeping 34
10.1 Data Collection 34
10.2 Archiving 35
11. Quality Management 35
11.1 Site Set-up and Initiation 35
11.2 On-site Monitoring 35
11.3 Central Monitoring 35
11.4 Audit and Inspection 36
11.5 Notification of Serious Breaches 36
12. End of Trial Definition 36
13. Statistical Considerations 36
13.1 Definition of Outcome Measures 36
13.1.1 Primary outcome measures 36

13.1.2 Secondary outcome measures 36
13.1.3 Exploratory Outcome Measures 37
13.2 Analysis of Outcome Measures 37
13.3 Planned Interim Analysis 37
13.4 Planned Final Analyses 37
13.5 Sample Size and Power Calculations 38
14.Trial Organisational Structure 38
14.1 Sponsor 38
14.2 Coordinating Centre 38
14.3 Trial Management Group 38
14.4 Trial Steering Committee 38
14.5 Data Monitoring Committee 38
14.6 Finance 38
15.Ethical Considerations 39
16.Confidentiality and Data Protection 39
17.Insurance and Indemnity 39
18.Publication Policy 40
19.Reference List 41
Appendix 1 - WMA Declaration of Helsinki 44
Appendix 2 - Definition of Adverse Events 47
Appendix 3 - Common Toxicity Criteria Gradings 49
Appendix 4 – HLH Diagnostic Criteria 50

1. BACKGROUND AND RATIONALE

1.1 Lay Summary

Haemophagocytic lymphohistiocytosis (HLH) and macrophage activation syndrome (MAS) are severe and life-threatening activations of the immune system that happen alongside cancer and infections in children. Current treatment for these conditions is based on chemotherapy but the outlook is extremely poor with 25-50% of patients failing to respond and eventually dying of disease. The current treatment strategy is an untargeted immunosuppressive approach and no effective new therapies have been developed.

Research conducted by the Mussai/De Santo research group has shown that in HLH/MAS there is an expansion of immune cells which drive the disease. A similar effect is also observed by a number of established research groups in a wide range of aggressive cancers where the cells stop the child's immune system from working effectively against the cancer. These cells can be identified by a marker called CD33 on the cells.

Importantly, an existing drug called gemtuzumab ozogamicin can specifically target CD33 on these immune cells leading to eradication of the cells. The GOTHAM trial is the first trial to test gemtuzumab ozogamicin specifically in this population and will test the activity of gemtuzumab ozogamicin in improving the outcomes for adults or children with HLH/MAS (10 patients) and relapsed/refractory (R/R) cancer (10 patients). Gemtuzumab ozogamicin will be given at 21-day intervals for 6 weeks and will measure the change in the number of the CD33 positive immune cells in the blood, survival and other markers of disease. This trial could provide a new therapy for children who otherwise have no other treatment options.

1.2 Background

Despite significant improvements in the survival of adults and children with some cancers, many of these patients will relapse and have poor outcomes with current treatment approaches. Therapeutic strategies for relapsed and refractory malignancies are limited, frequently re-combining drugs which are used in front-line treatment or drugs with similar mechanisms of cytotoxicity. In many cases the benefits of dose-intensification of these agents have similarly been maximised leading to significant acute and chronic toxicities for patients. Therapeutic strategies, which target malignancies through new mechanisms, and that have a low burden of toxicity are urgently needed.

Cancer cells exist within an immune microenvironment, containing populations of cells which may act to coordinate and target the cancer or in contrast be subverted to promote cancer growth and survival [1] . The increasing use of immunotherapies to boost patients' autologous immune responses against cancer has seen some dramatic clinical responses [2], however in many cases, notably paediatrics, responses can be short-lived or non-existent [3]. This failure is due to the presence of an immunosuppressive microenvironment that inactivates immunotherapy responses [4]. One of the major drivers of immunosuppression in cancer patients is the expansion of populations of myeloid cells in the blood and tissue, which cross-talk through cell-cell signalling, release of factors, or consumption of amino acids to inactivate surrounding cells [5-7]. Although strategies to target these immunosuppressive mechanisms have been trialled, they are limited by the targeting of only single mechanism and by a failure to eradicate the expanded alternatively activated myeloid population [8].

Myeloid cells also play critical roles in driving the pathology of other conditions. HLH/MAS are rare conditions secondary to cancer, infection, or autoimmunity resulting in immune activation and severe, life-threatening systemic inflammation [9, 10]. Expansions of myeloid cells which secrete pro-inflammatory factors are central to the underlying pathology of these conditions, yet to date no therapies have directly targeted this driver [11]. As a rare and non-malignant disease few treatments have been developed which rationally target the underlying biological mechanisms [12]. Trials of new drugs in such patients pose a challenge due to the relatively low numbers of patients and a lack of commercial interest from major pharmaceutical companies.

Academic-investigator led, multi-national clinical trials of novel drugs therefore play a pivotal role in improving the outcomes for both these patient groups. Here we propose a trial to target the myeloid cells which are central to the pathophysiology of cancer and HLH/MAS.

1.2.1 Preclinical background

The resurgence in immunotherapy approaches for adult and paediatric cancers has highlighted the potential of the immune system to detect and remove cancer cells. In adult malignancies antibodies against cell surface antigens [13] and immune checkpoint molecules [14], or immunotoxins [15, 16] have significantly improved clinical response rates for a range of cancer types. In paediatrics Bi-specific T cell engager antibodies (BITEs) [17] and Chimeric-Antigen Receptor T (CAR-T) [18] cells targeting B-Acute Lymphoblastic Leukaemias have generated startling clinical responses and cures, even in multiply relapsed patients. However, such dramatic responses are not universal. Anti-immune checkpoint antibodies have demonstrated minimal activity in a number of clinical trials in children and despite the impressive results in B-ALL CAR-T cell activity in adult solid cancers, and paediatric cancers such as neuroblastoma remains poor.

Tumours use a number of mechanisms to resist the immune response of both patients and engineered therapies. These include reducing the capacity for antigen presentation by downregulation of Major histocompatibility complex (MHC) Class I and II or surface CD molecular targets, upregulation of surface molecules which engage and inactivate immune cells, release of cytokines, and modulation of surrounding immune and stromal cells to an immunosuppressive phenotype. Altering metabolism of amino acids, glucose, and oxygen within the tumour microenvironment can also manipulate immune function [19].

One of the major mechanisms of tumour-immune escape, is through the expansion of alternatively activated and immunosuppressive myeloid cells [20]. Myeloid cell populations include macrophages, monocytes, granulocytes, and Myeloid-Derived Suppressor Cells (MDSCs). It is well established that these cells may be significantly expanded in the blood and tumours of adults and children with solid malignancies, regardless of tumour type and are associated with higher stage, metastatic disease, and a worse prognosis. These cells have been polarised by the tumour microenvironment to switch off autologous anti-cancer T and natural killer (NK) cells' responses and can impair the function of immunotherapies [21]. These myeloid cells engage a number of different mechanisms to suppress the immune response including deprivation of amino acids such as arginine and cysteine, secretion of Reactive Oxygen and Nitric Oxide species [4], release of immunomodulatory cytokines like interleukin (IL)-10, IL-4, IL-6, IL-1b [22, 23] and expression of cell surface receptors like PDL1. The major challenges in targeting such cells is that each population has a unique immunophenotype based on expression of cell surface molecules such as: CD68/CD206/CD163 (macrophages), CD14 (monocytes/ M-MDSCs), CD15 (granulocytes, G-MDSCs), MDSCs (CD11b, HLA-Dr low/- in combination with CD14 or CD15); more than one myeloid cell type is contributing to immunosuppression in each individual patient, and finally that different populations may be expanded in different cancer patients even of the same subtype.

More rarely abnormal expansion and activation of circulating and tissue-infiltrating myeloid cells can lead to a severe and life-threatening systemic inflammation - a condition known as HLH. Primary HLH may be due to underlying genetic mutations in genes regulating lymphocyte cytotoxicity [24], whilst secondary HLH is associated with cancers, and also infectious or autoimmune conditions [10]. The later may include a spectrum of clinical findings termed MAS [9]. Recently the diagnosis of MAS has become notable due to the use of immunotherapy approaches in cancer patients treated with Chimeric-Antigen Receptor T cells or antibodies [25, 26]. Regardless of cause HLH/MAS is diagnosed using criteria that include the presence of cytopenias, fever, splenomegaly, high ferritin, high triglycerides, and low fibrinogen. Immune malfunction such as increased sCD25, low NK activity, haemophagocytosis in the bone marrow, and raised inflammatory cytokines such as IL-1β, IL-6, and TNF-α may also be present.

1.2.2 Preclinical rationale for gemtuzumab ozogamicin

In murine models monocytic MDSCs and tumour-associated macrophages can be defined by their expression of Ly6C and CCR2, or granulocytic MDSCs by the expression of Ly6G surface antigens [27]. Administration of antibodies against these surface antigens leads to a systemic depletion of myeloid cells, and a reactivation of normal T cell responses against cancers or infection in vivo – providing proof of principle that antibody targeting of these cells can have a dramatic and helpful effect on immunity [28, 29]. However, in humans, therapeutic targeting of MDSCs and Tumour associated macrophages (TAMs) has focused on targeting the intracellular pathways of immunosuppression or myeloid cell induction [8], in many cases with drugs which are not well tolerated or which are only applicable to one disease sub-type.

CD33 is a Sialic-Acid-Binding-immunoglobulin-like lectin (SIGLEC), composed of a type 1 membrane protein with two immunoglobulin domains that binds sialic acid and intracellular immunoreceptor tyrosine-based inhibitory motifs (ITIMs) [30, 31]. Binding of sialic acid ligands to CD33 can induce a number of physiological function resulting in inhibition of cellular proliferation and activation, apoptosis, or modulation of cytokine release [31]. Although in humans monocytes, MDSCs, and macrophages may be differentiated from each other by the relative expression of cell surface molecules such as CD14, HLA-DR, CD68, CD163, and CD206 and by their functional phenotypes, these cells do commonly express CD33 providing a universal therapeutic target [6, 32].

CD33 may be targeted through unconjugated antibodies, however these antibodies are not usually cytotoxic and do not lead to sustained depletions of the target cells thus minimising any therapeutic benefit [33, 34]. However one approach to enhancing antibody-induced cytotoxicity is by antibody-toxin [16] or drug conjugates. Gemtuzumab ozogamicin is an anti-CD33 humanized murine antibody conjugated to the cytotoxic antibiotic calicheamicin [35]. On binding of gemtuzumab ozogamicin to CD33 positive cells, the drug is rapidly internalised followed by release of calicheamicin intracellularly. Calicheamicin induces double-stranded DNA breaks and apoptosis. Gemtuzumab ozogamicin has predominantly been tested against acute myeloid leukemia (AML) blasts. It demonstrates dose-dependent cellular cytotoxicity against CD33+ blasts with no activity against CD33- cells.

Analogous to findings in AML blasts, the Mussai group at the University of Birmingham have demonstrated that gemtuzumab ozogamicin can also bind to CD33 on cells of the monocytic lineage (pro-inflammatory monocytes, tumour associated monocytes/macrophages and MDSCs) found in HLH/MAS and different cancer patients [36]. The gemtuzumab ozogamicin is rapidly internalised leading to target cell death without non-specific toxicity to normal immune cells such as T cells and neutrophils which do not express CD33. These findings are supported by initial control experiment published during gemtuzumab ozogamicin’s early translational development even though they were not clinically exploited [37]. In the case of HLH/MAS-associated CD33+ cells the result of gemtuzumab ozogamicin treatment is also a decrease in IL-1, IL-6 and TNF-α ex vivo and improvements in haematopoiesis and splenomegaly in vivo [38]. In cancer patients CD33+ targeting with GO leads to a restoration of normal T cell proliferation and activation, as the immunosuppressive microenvironment unique to these patients is removed. Thus gemtuzumab ozogamicin is able to specifically target and kill the CD33+ myeloid cells which are central to the pathology of HLH/MAS and restore normal immune function by cytotoxicity to cancer-associated MDSCs/TAMs.

1.2.3 Clinical background

Activity of gemtuzumab ozogamicin in adult and childhood AML

Gemtuzumab ozogamicin has a European Union wide marketing authorisation as MYLOTARG (Pfizer and is indicated for use in combination with daunorubicin and cytarabine chemotherapy for the treatment of patients age 15 years and above with previously untreated types of CD33-positive AML.

In the USA gemtuzumab ozogamicin is Federal Drug Administration (FDA) approved for the treatment of newly-diagnosed CD33-positive AML in adults and for treatment of relapsed or refractory CD33-positive AML in adults and in paediatric patients 2 years and older. It may be used in combination with daunorubicin and cytarabine for adults with newly-diagnosed AML, or as a stand-alone treatment for certain adult and paediatric patients.

The recommended licensed dose of gemtuzumab ozogamicin is 3 mg/m2/dose (up to a maximum of one 5 mg vial) infused over a 2-hour period on Days 1, 4, and 7.

In paediatrics, several studies [39-43], including the MyeChild 01 trial, sponsored by the University of Birmingham, has reported that in patients over 1 year of age 3 doses of 3 mg/m2 gemtuzumab ozogamicin combined with induction chemotherapy is well tolerated in this patient population, consistent with data from adult AML studies. As a result patients over 1 year of age will be included in the GOTHAM trial [44].

Pharmacokinetics of gemtuzumab ozogamicin in paediatric patients follows a similar profile and variability to that of adult patients [45, 46].

GOTHAM will be one of the first clinical trials using gemtuzumab ozogamicin for the targeting of pathological non-malignant myeloid cells.

Principal toxicity data in adults and children:

Adults:

As documented in the summary of product characteristics (SmPC) for gemtuzumab ozogamicin:

The overall safety profile of gemtuzumab ozogamicin is based on data from patients with AML from the combination therapy trial ALFA-0701 [47], monotherapy studies, and from post-marketing experience. In the combination therapy trial, safety data consisting of selected treatment emergent adverse events (TEAEs) considered most important for understanding the safety profile of gemtuzumab ozogamicin consisted of all grades haemorrhages, all grades veno-occlusive disease (VOD), and severe infections. All of these TEAEs were determined to be adverse drug reactions (ADRs).

In the combination therapy trial ALFA-0701, clinically relevant serious adverse reactions were hepatotoxicity, including VOD/sinusoidal obstructive syndrome (SOS) (3.8%), haemorrhage (9.9%), severe infection (41.2%), and tumour lysis syndrome (1.5%). In monotherapy studies, clinically relevant serious adverse reactions also included infusion related reactions (2.5%), thrombocytopenia (21.7%), and neutropenia (34.3%).

The most common adverse reactions (> 30%) in the combination therapy trial were haemorrhage and infection. In monotherapy studies the most common adverse reactions (> 30%) included pyrexia, nausea, infection, chills, haemorrhage, vomiting, thrombocytopenia, fatigue, headache, stomatitis, diarrhoea, abdominal pain and neutropenia.

Paediatric:

In the completed randomised paediatric Phase 3 Trial AAML0531 [48] of gemtuzumab ozogamicin combined with intensive first-line therapy in 1,063 newly diagnosed children (93.7% of patients <18 years of age), and young adults (6.3% of patients) with de novo AML aged 0 to 29 years, the safety profile was similar with that observed in the other studies of gemtuzumab ozogamicin combined with intensive chemotherapy in adult patients with de novo AML.

The most frequent (≥ 1%) adverse reactions that led to permanent discontinuation in monotherapy studies were infection, haemorrhage, multi-organ failure, and VOD.

1.3 Trial Rationale

1.3.1 Justification for patient population

HLH and MAS

Haemophagocytic lymphohistiocytosis (HLH) and macrophage activation syndrome (MAS) are severe and life-threatening activations of the immune system that happen alongside cancer, inflammation and infections in adults and children. Clinically, patients present with a spectrum of findings and in cases of

advanced or relapsed/refractory disease may necessitate admission to the intensive care unit for management of organ dysfunction. HLH is usually defined according to the HLH-2004 diagnostic criteria from the Histiocyte Society [49, 50] (See Appendix 4). Although no consensus criteria for MAS are well established, many of the same HLH parameters are used as a guide to the diagnosis [51, 52]. In both diseases other findings such as significant transaminitis and hyperbilirubinemia, renal dysfunction, and neurological abnormalities may also develop.

Treatment for primary HLH is based on multi-drug chemotherapy (HLH-2004 guidelines) with the crude goal to suppress the immune response [24]. For secondary HLH and MAS the best approach is believed to be through treatment of the underlying initiating condition (e.g. cancer, arthritis, infection). The acute morbidity is high with the majority of patients undergoing treatment which frequently results in intensive care admission [53, 54]. Furthermore in both HLH and MAS the outlook is extremely poor with 25-50% of patients failing to achieve a complete remission and dying of disease [54-57]. Current salvage strategies are sub-optimal and frequently based on anecdotal evidence. Such therapies include anti-thymocyte globulin or alemtuzumab (targeting T cells), and anti-cytokine antibodies such as infliximab (TNF-α), tocilizumab (IL-6), emapalumab (IFN-g) and anakinra (IL-1) [12]. Notably these approaches do not target the macrophages/monocytes which are at the centre of the pathology and drive the clinical condition. This trial would provide a therapeutic approach which could target the myeloid cells that drive the life-threatening inflammation in HLH/MAS.

<u>R/R solid cancers in adults and children</u>

Myeloid cells are expanded in the blood and tumours of all major categories of adult and paediatric tumours. As described above these cells are central to tumour pathophysiology and limit the ability of the patients' own immune responses to attack and destroy cancer cells. Gemtuzumab ozogamicin offers a therapy to universally target the populations of expanded, immunomodulatory myeloid cells in cancer patients and would provide a specific way to reactivate the autologous immune response against cancer/ act as an adjunct to immunotherapies to enhance their response.

1.3.2 Justification for design

The purpose of this trial is to provide initial evidence on whether single agent gemtuzumab ozogamicin is safe and has activity in adult or paediatric HLH/MAS and relapsed/refractory solid tumours. There is no *a priori* reason to believe that the activity of gemtuzumab ozogamicin will vary according to patient or tumour type due to the preclinical evidence showing that CD33 expression is stable, constitutive and prevalent on the relevant myeloid cell populations across multiple adult and paediatric tumour types or HLH/MAS.

The pharmacokinetic (PK) profiles of gemtuzumab ozogamicin in adult and paediatric trials have been reproducible with a predictable rise in gemtuzumab ozogamicin with weekly dosing at 3 mg/m^2 concentration and a sustained depletion of CD33+ cells in the patients with AML. Thus the PK relationship is not expected to be significantly different in our patient populations if the adult and paediatric recommended phase 2 dose (R2PD) of 3 mg/m^2 is used.

Although no patients with HLH/MAS have been treated with gemtuzumab ozogamicin to date, and such patients may have deranged liver function tests before commencing the trial, these patients often have no alternative treatment strategies left except palliation. As such the risk-benefit of reducing myeloid-cell induced liver inflammation with this drug is an important factor in the trial's decision to include such patients. Close monitoring of patients' liver parameters will take place as detailed in the protocol. As liver derangements in HLH/MAS patients may be grossly abnormal due to their disease it is not possible to apply an exact scientifically justified threshold for the upper limits of acceptable liver function in this population.

2. AIMS, OBJECTIVES AND OUTCOME MEASURES

2.1 Aims and Objectives

Primary Objective

- To assess the activity of gemtuzumab ozogamicin 3 mg/m^2/dose given on Days 1, 22, 43 by measuring the change in CD33+ myeloid cells in the blood in 2 parallel groups.

Secondary Objectives

- To assess the effect of gemtuzumab ozogamicin on overall survival time (OS) and progression free survival time (PFS) (Group 2 only).
- To assess the feasibility of delivering gemtuzumab ozogamicin

Exploratory Objectives

- To assess the change in IL-1/IL-6/ TNF-α in the plasma
- To assess the change of CD33+ cells in the bone marrow/ tumour tissue (as available)

2.2 Outcome Measures

Primary Outcome Measures

- CD33+ cell count in the blood samples of patients collected at the pre-specified time points Days 1, 8, 15, 22, 29, 43, 50 and 57

Secondary Outcome Measures

- OS time
- PFS time (Group 2 only)
- Incidence of grade 3 and 4 adverse events

Exploratory Outcome Measures

- Change in IL-1/IL-6/ TNF-α in the plasma
- Change of CD33+ cells in the bone marrow/ tumour tissue (as available)

3. TRIAL DESIGN

GOTHAM is a single arm, open-label, phase II trial in patients with R/R HLH or MAS (Group 1) or patients with relapsed/refractory (R/R) solid cancers (Group 2).

A minimum of 10 evaluable patients will be recruited to each of the 2 patient groups. All patients will be treated with gemtuzumab ozogamicin administered as an intravenous infusion on Days 1, 22, and 43 at a starting dose of 3 mg/m^2/dose. Any patients who withdraw from the trial or who die prior to collection of a CD33+ blood sample on day 8 may be replaced.

CD33+ levels will be measured serially for each patient before and after treatment.

Patients will be considered to be evaluable once they have been treated with at least 1 dose of gemtuzumab ozogamicin, and CD33+ blood samples have been collected on day 1 of treatment and at one other future time point.

4. ELIGIBILITY

4.1 Inclusion Criteria

- Aged >1 year old at the time of trial entry
- Diagnosis of primary or secondary HLH or MAS disease that is relapsing/refractory to treatment at time of enrolment (Group 1)
  OR
  Histologically confirmed diagnosis of solid cancer with radiological or clinical evidence of disease progression (during or after completion of at least one previous treatment) or any subsequent recurrence (biopsy at relapse is not mandated) (Group 2)

Note: patients who meet the inclusion criteria for both groups 1 and 2 should be entered into group 1

- Group 2 only – must have adequate liver function:
  - Total bilirubin ≤ x2 upper limit of normal (ULN)
  - Aspartate aminotransferase *(*AST) and/or alanine aminotransferase (ALT) ≤ 2.5 x ULN
- Documented negative pregnancy test for female patients of childbearing potential within 7 days prior to trial entry
- Sexually active patients must agree to use 2 methods of adequate and appropriate contraception while on trial drug and for 4 months (male) and 7 months (female) following treatment discontinuation[1]
- Written informed consent given by patient and/or parents/legal guardian

[1] Patients with reproductive potential must agree to use two adequate methods of birth control during the period of therapy. Men should be advised not to father a child for 4 months after receiving the last dose of trial treatment. Women of childbearing potential should be advised to use effective contraception to avoid pregnancy for 7 months after the last dose of trial treatment. Effective contraceptive methods include implants, injectables, combined oral contraceptives, intrauterine device (IUD or coil), bilateral tubal occlusion, true sexual abstinence or vasectomised partner, progestogen-only oral hormonal contraception where inhibition of ovulation is not the primary mode of action, male or female condom with or without spermicide and cap, diaphragm or sponge with spermicide. Sperm or egg preservation should be offered as per standard practice to patients at risk of irreversible infertility, where appropriate. Sexual abstinence must be in line with the preferred and usual lifestyle of the patient. Periodic abstinence (e.g. calendar, ovulation, symptothermal, post-ovulation methods), declaration of abstinence for the duration of the trial, and withdrawal are not acceptable methods of contraception.

4.2 Exclusion Criteria

- Evidence of sinusoidal obstruction syndrome (SOS) / veno-occlusive disease (VOD)
- Previous treatment with another CD33 targeting antibody or immunotoxin
- Hypersensitivity to Gemtuzumab Ozogamicin or to any of the excipients
- Pregnant or lactating female

5. SCREENING AND CONSENT

5.1 Screening

Investigators will be expected to maintain a screening log of all potential trial participants. This log will contain limited information about the potential participant and will include the date and outcome of the screening process.

The Investigator will provide trial information to patients and/or parents/legal guardians of children who are considered to meet the trial eligibility criteria. This information should be sufficient to allow patients/parents/legal guardians to make an informed decision about participation. If informed consent is obtained, the Investigator will conduct a full screening evaluation to ensure that the patient meets all inclusion and exclusion criteria.

Note that assessments conducted as standard of care do not require informed consent and may be provided as screening data if conducted within an appropriate time-scale.

A complete list of assessments at diagnosis/relapse/recurrence is given in the Schedule of Events.

5.2 Informed Consent

It is the responsibility of the Investigator, or person to whom the Investigator delegates the responsibility, to obtain written informed consent for each patient/parent/legal guardian prior to performing any trial related procedure. A Patient Information Sheet is provided to facilitate this process. Where this responsibility has been delegated, this must be explicitly stated on a Site Signature and Delegation Log (SSDL). Parent/Patient Information Sheets (PIS) are provided.

Investigators must ensure that they adequately explain the trial aims, trial treatment, anticipated benefits and potential hazards of taking part in the trial to the patient/parent/legal guardian. It must be clearly explained to the patient/parent/legal guardian that participation in the biomarker studies is mandatory given its scientific relevance.

The Investigator should make it absolutely clear that the patient/parent/legal guardian is completely free to refuse to take part or withdraw from the trial at any time. The patient/parent/legal guardian should be given adequate time to read the PIS and to discuss their participation with others outside of the site research team should they wish to do so. However, because of the acute nature of HLH/MAS, the available time for some patients may be less than 24 hours. The patient/parent/legal guardian must be given an opportunity to ask questions which should be answered to their satisfaction. The right of the patient/parent/legal guardian to refuse to participate in the trial without giving a reason must be respected.

If the patient/parent/legal guardian expresses an interest in them/their child participating in the trial they should be asked to sign and date one copy of the latest approved version of the Informed Consent Form (ICF). The investigator or designate must then sign and date the form on the same day as the patient/parent/legal guardian. Written assent will also be obtained from patients under the age of 16 years wherever it is possible to do so using the relevant section on the Parent ICF. For those children who are not able to read or write or understand the ICF, where it is deemed appropriate for them to provide assent, the clinician will explain the trial and obtain verbal assent where possible and this will be documented in the medical notes. Patients should be re-consented at the age of majority.

A copy of the ICF should be given to the patient or parent/legal guardian, a copy should be filed in the patient's medical records, and the original placed in the Investigator Site File (ISF) or country specific equivalent (henceforth referred to as ISF). Once the patient has been entered into the trial, the patient's trial number should be entered on the ICF maintained in the ISF. If the patient/parent/legal guardian has given explicit consent, a copy of the signed ICF should be sent in the post to the Trials Office for review.

Details of the informed consent/assent discussions should be recorded in the patient's medical records. This should include date of, and information regarding, the initial discussion, the date consent was obtained, the trial name and the version number of the PIS and ICF. Throughout the trial, the patient and/or parent/legal guardian should have the opportunity to ask questions about the trial and any new information that may be relevant to the patient's continued participation should be shared with them in a timely manner. On occasion it may be necessary to re-consent the patient, for example if new information becomes available or an amendment is made to the protocol that might affect the patient's participation in the trial. In this case the process above should be followed and the patient's right to withdraw from the trial respected.

Electronic copies of the PIS and ICF are available from the Trials Office and should be printed or photocopied onto the headed paper of the local institution.

Details of all patients approached about the trial should be recorded on a screening and enrolment log. With the patient's and/or parent's/legal guardian's consent, their General Practitioner (GP) should be informed of their trial participation. A GP letter is provided electronically for this purpose.

5.3 Contraception

Females of and males with partners of childbearing potential (i.e. not post-menopausal or surgically sterilised) must use 2 methods of adequate and appropriate contraception while on trial drug and following treatment discontinuation. Men should be advised not to father a child for 4 months after receiving the last dose of trial treatment. Women of childbearing potential should be advised to use effective contraception to avoid pregnancy for 7 months after the last dose of trial treatment.

Effective contraceptive methods include implants, injectables, combined oral contraceptives, intrauterine device (IUD or coil), bilateral tubal occlusion, true sexual abstinence or vasectomised partner, progestogen-only oral hormonal contraception where inhibition of ovulation is not the primary mode of action, male or female condom with or without spermicide and cap, diaphragm or sponge with spermicide. Sperm or egg preservation should be offered as per standard practice to patients at risk of irreversible infertility, where appropriate. Sexual abstinence must be in line with the preferred and usual lifestyle of the patient. Periodic abstinence (e.g. calendar, ovulation, symptothermal, post-ovulation methods), declaration of abstinence for the duration of the trial, and withdrawal are not acceptable methods of contraception.

Patients should not breast feed for at least one month after treatment discontinuation.

6. TRIAL ENTRY

Patients may be entered into the trial by a treatment site once the Trials Office has confirmed that all regulatory requirements have been met by the site and the site has been formally activated by the Trials Office.

Before approaching a potential patient, Investigators should contact the GOTHAM Trials Office to check that there is a slot available in the applicable treatment group.

Registration into the trial should be performed by sites using the GOTHAM online electronic Remote Data Entry Capture (eRDC) system which has been developed by the Cancer Research UK Clinical Trials Unit (CRCTU), University of Birmingham.

https://www.cancertrials.bham.ac.uk/GOTHAMLive

In order to perform registration, the GOTHAM eligibility Checklist must be completed and signed by an Investigator. A copy of the registration report should be printed out and filed in the Investigator Site File (ISF), and the registration documented in the patient's notes.

In the event of a problem with the online trial entry, the Eligibility Checklist and Registration Form should be completed. These details can be emailed to the Trials Office using the email below numbers below.

**REGISTRATION**
✉ GOTHAM@trials.bham.ac.uk
09:00 TO 17:00 GMT/BST, Monday to Friday

The Trial Number will be used to identify the patient and should be recorded on all further correspondence with the Trials Office. The Trial Number should also be documented on the original signed ICF filed in the ISF.

7. TREATMENT DETAILS

7.1 Investigational Medicinal Products

Gemtuzumab ozogamicin is the only investigational medicinal product (IMP).

Chemical structure: Gemtuzumab ozogamicin is an antibody-drug conjugate composed of the CD33-directed monoclonal antibody (hP67.6; recombinant humanised immunoglobulin G4, kappa antibody produced by mammalian cell culture in NS0 cells) that is covalently linked to the cytotoxic agent N-acetyl gamma calicheamicin.

Mechanism of action: Gemtuzumab ozogamicin binds cell surface CD33 on target cells and is rapidly internalised. The calicheamicin is cleaved from the scFV within the endosomes, leading to DNA damage and cell death.

Gemtuzumab ozogamicin will be provided free of charge to sites by Pfizer Ltd. Full details of supply, storage preparation, labelling and accountability are contained in the Pharmacy Manual.

7.2 Treatment Schedule

Gemtuzumab ozogamicin is administered as 3 doses (3 mg/m^2/dose: up to a maximum of one 5 mg vial) on days 1, 22 and 43, infused over a 2-hour period. It must be administered as an infusion not as an intravenous push or bolus.

Treatment should be administered under the supervision of a physician experienced in the use of anticancer products and in an environment where full resuscitation facilities are immediately available.

Patients in Group 1 (HLH/MAS) must be treated in an inpatient setting; patients in Group 2 (R/R solid cancers) may be administered as an inpatient or outpatient depending on the patient's clinical assessment by the responsible physician.

The dose must be administered at 21 day intervals (± 3 days) to ensure sustained adequate myeloid cell depletion. Further alteration to dosing schedule should be discussed with the Chief Investigator.

Gemtuzumab ozogamicin should be given for 3 doses only.

Doses of gemtuzumab ozogamicin are based on Body Surface Area (BSA). BSA should be calculated in accordance with institutional practice.

See section 7.7 for dose modifications.

7.3 Pre-Medication

Pre-medication with methylprednisolone 1mg/kg (for all ages), chlorpheniramine 4-8mg po/IV (dose dependent on age) and paracetamol (15mg/kg, max 1g) is recommended 1 hour prior to dosing to help prevent infusion-related reactions.

7.4 Warnings for use

- In clinical studies infusion related reactions, including anaphylaxis were reported. There have been reports of fatal infusion reactions in the post-marketing setting. Signs and symptoms of infusion related reactions may include fever and chills, and less frequently hypotension, tachycardia, and respiratory symptoms that may occur in the first 24 hours after

administration. Infusions of gemtuzumab ozogamicin should be performed under close clinical monitoring.

- Vital signs (including pulse, blood pressure, and temperature) should be monitored during the infusion. A gemtuzumab ozogamicin infusion should be interrupted immediately in patients who develop evidence of severe reactions, especially dyspnoea, bronchospasm or clinically significant hypotension. Patients should be monitored until signs and symptoms completely resolve.

- Discontinuation of treatment should be strongly considered for patients who develop signs or symptoms of anaphylaxis, including severe respiratory symptoms or clinically significant hypotension.

- See also section 7.7

- Patients should be monitored for signs and symptoms of SOS/VOD and treated according to institutional practise (see section 7.7)
- Patients who receive gemtuzumab ozogamicin either before or after an haematopoietic stem cell transplant (HSCT), and patients with moderate or severe hepatic impairment are at increased risk for developing SOS/VOD (see section 7.7)
- Patients should be monitored for signs and symptoms of tumour lysis syndrome (TLS) and treated according to institutional practice (see section 7.7)
- Gemtuzumab ozogamicin has moderate influence on the ability to drive and use machines. Patients should be advised they may experience fatigue, dizziness and headache during treatment.

7.5 Assessments

Assessments should be carried out in accordance with the Schedule of Events in the Trial Synopsis. Further patient monitoring can be performed according to institutional practice.

7.6 Sample Collection

			Days from first dose **Samples must be taken pre-dose on the day of treatment**							
Sample	Screening[1]	Pre-dose 1 (Day 1)	Day 8	Day 15	Pre-dose 2 (Day 22)	Pre-dose 3 (Day 43)	Day 29	Day 50	Day 57	At relapse / progression
Whole blood for CD33 analysis (5 ml)[3]		✓	✓	✓	✓	✓	✓	✓	✓	✓
Whole blood for translational research (9 ml for children, 30 ml for adults)[3]		✓	✓	✓	✓	✓	✓	✓	✓	✓
Fresh frozen tissue from bone marrow aspirate (group 1) or biopsy (group 2)[2]	✓ (if available)									✓ (if available)
Paraffin embedded tumour tissue (group 2)[2]	✓ (if available)									✓ (if available)

[1]Screening samples should not be sent for central collection/analysis until the patient has consented and enrolled on the GOTHAM trial

[2]Where available from bone marrow or biopsy and where procedure has been performed as part of standard of care

[3]Smaller volumes may be collected for children < 10 kg: see EMA guidelines for recommendations on trial related blood loss https://ec.europa.eu/health//sites/health/files/files/eudralex/vol-10/ethical_considerations_en.pdf

For details on sampling procedures, sample processing and shipment refer to the GOTHAM Laboratory Manual.

7.6.1 Blood samples for CD33+ analysis

The primary objective of the GOTHAM trial is to assess the activity of gemtuzumab ozogamicin on Days 1, 8, 15, 22, 29, 43, 50 and 57 in the change in CD33+ myeloid cells. This will be measured in the blood samples collected from trial patients as described in this section. Collection of these samples is therefore fundamental to the outcome of the trial, and considered mandatory.

Samples must be taken on the dosing days prior to treatment. If this is not possible please contact the laboratory (contact details at the front of the protocol) for advice.

7.6.2 Tumour tissue and blood sample collection for translational research

Where available from existing biopsies/surgeries tumour tissue should be provided (approx. 10 slides from paraffin embedded tumour tissue block) and fresh frozen tissue where available from bone marrow

aspirate or biopsy. Tumour tissue will be analysed for infiltration of CD33+ cells, T cells and tumour cells before and after treatment, as well as to assess biomarkers of response.

Whole blood should be collected from all patients for translational research. Samples will be collected at the same time as the samples for CD33+ analysis.

7.7 Dose Modifications

Doses of gemtuzumab ozogamicin should be capped at a maximum of 1 vial.

7.7.1 SOS/VOD

Hepatotoxicity, including SOS/VOD have been reported in patients treated with gemtuzumab ozogamicin.

Due to the risk of SOS/VOD, signs and symptoms of SOS/VOD should be closely monitored; these may include elevations in ALT, AST, total bilirubin, and alkaline phosphatase (ALP), which should be monitored prior to each dose of gemtuzumab ozogamicin, hepatomegaly (which may be painful), rapid weight gain, and ascites. Monitoring only total bilibrubin may not identify all patients at risk of VOD/SOS.

For patients who develop abnormal liver tests, more frequent monitoring of liver tests and clinical signs and symptoms of hepatotoxicity is recommended.

Note as liver derangements in HLH/MAS patients may be grossly abnormal due to their disease it is not possible to apply an exact scientifically justified threshold for the upper limits of acceptable liver function in this population prior to the first dose of Gemtuzumab ozogamicin.

Patients who experience SOS/VOD should discontinue trial treatment.

The following is a guideline for identifying SOS/VOD in paediatric patients.

Where cases are suspected, this can be further discussed with the GOTHAM chief investigator.

There is no limitation for time of onset of SOS/VOD.

Diagnosis is based on the presence of two or more of the following: [a]

- Unexplained consumptive and transfusion-refractory thrombocytopenia [b]
- Otherwise unexplained weight gain on three consecutive days despite the use of diuretics or a weight gain >5% above baseline value
- Hepatomegaly (best if confirmed by imaging) above baseline value [c]
- Ascites (best if confirmed by imaging) above baseline value [c]
- Rising bilirubin from a baseline value on 3 consecutive days or bilirubin ⩾2 mg/dL within 72 hours

[a] With the exclusion of other potential differential diagnoses.
[b] ⩾1 weight-adjusted platelet substitution/day to maintain institutional transfusion guidelines.
[c] Suggested: imaging ultrasonography, computed tomography (CT) or magnetic resonance imaging (MRI) immediately before hematopoietic cell transplantation (HCT) to determine baseline value for both hepatomegaly

7.7.2 Haematopoietic stem cell transplant (HSCT), moderate/severe liver impairment and SOS/VOD

Patients who receive gemtuzumab ozogamicin either before or after an HSCT and patients with moderate or severe hepatic impairment are at increased risk for developing SOS/VOD.

For patients who proceed to HSCT, close monitoring of liver tests is recommended during the post-HSCT period, as appropriate and as per institutional practise.

Table 1: Dose modifications for toxicity

Toxicity	Modification
VOD/SOS	Discontinue gemtuzumab ozogamicin
Group 1: Liver toxicity (not including deranged LFTs and Bilirubin prior to the first dose of Gemtuzumab ozogamicin)	If AST/ALT and Bilirubin are not stable or improved from baseline prior to dose 2 or dose 3, then postpone Gemtuzumab ozogamicin until baseline parameters are reached, In the event this does not occur no further Gemtuzumab ozogamicin should be administered
Group 2: Liver toxicity (not including SOS/VOD) with suspected causal relationship to gemtuzumab ozogamicin	Postpone gemtuzumab ozogamicin treatment until recovery of total bilirubin to ≤2 x ULN and ≤2.5x AST/ALT ULN prior to each dose.
Infusion related reactions	See section 7.4<br>Interrupt the infusion and institute appropriate medical management based on the severity of symptoms. Patients should be monitored until signs and symptoms completely resolve and infusion may resume.<br>Consider permanent discontinuation of treatment for severe or life-threatening infusion reactions.
Thrombocytopenia/neutropenia	See supportive treatment section 7.9
Other severe or life-threatening non-haematological toxicities	Delay treatment with gemtuzumab ozogamicin until recovery to a severity of no more than mild.

7.8 Treatment Compliance

Compliance with gemtuzumab ozogamicin treatment will be monitored by the Trials Office and as specified in the GOTHAM Pharmacy Manual and using data on the Case Report Form (CRF).

7.9 Supportive Treatment

See section 7.3 for pre-medication

Other supportive care should be given according to local guidelines/institutional policy.

7.9.1 Nausea and Vomiting

Significant nausea and vomiting is not an expected side-effect of gemtuzumab ozogamicin and routine anti-emetic use is not indicated. If required, anti-emetics may be given according to local guidelines/institutional policy.

7.9.2 Fever and Neutropenia

Management of fever and neutropenia, including choice of antibiotics should be on the basis of local guidelines/institutional policy.

7.9.3 Thrombocytopenia, decreased haemoglobin or infection

This should be managed according to local guidelines/institutional policy.

7.9.4 Diarrhoea

This should be managed according to institutional policy to avoid dehydration and other complications.

7.9.5 Allergic reaction

In case of an allergic reaction during gemtuzumab ozogamicin administration, the infusion should be stopped and medication for allergic reaction given as per local guidelines/institutional policy. See Table 1: Dose modifications for toxicity.

7.9.6 Blood products

Therapeutic use of blood products is permitted and should be administered according to local guidelines/institutional policy.

7.9.7 GCSF / GM-CSF

Use of Granulocyte-colony stimulating factor (GCSF) and Granulocyte-macrophage colony-stimulating factor (GM-CSF) is not permitted, as it may counter the myelo-reductive effects of gemtuzumab ozogamicin.

7.9.8 Tumour lysis syndrome

Manage according to institutional practice.

7.9.9 SOS/VOD

Manage according to institutional practice.

7.10 Concomitant Medication

For Group 1 (HLH/MAS) – gemtuzumab ozogamicin may be given alongside or following other treatments used to manage the condition. Other anti-CD33 targeting or depleting agents are not permitted.

For Group 2 (R/R tumours) – gemtuzumab ozogamicin should be given as a single agent and not alongside other cytotoxic or immunomodulatory drugs. Other anti-CD33 targeting or depleting agents are not permitted.

Other concomitant medication may be given as medically indicated. Palliative and supportive care for disease-related symptoms should be offered to all patients when appropriate.

7.11 Patient Follow Up

Following completion of treatment, follow-up assessments should be conducted as per local practice. Patients will be followed-up for a minimum of 1 year.

8. TREATMENT DISCONTINUATION AND PATIENT WITHDRAWAL

8.1 Discontinuation of gemtuzumab ozogamicin

If a patient stops GOTHAM protocol treatment prematurely, the reason should be recorded in the patient's medical records and should be reported on the CRF. Reasons for stopping protocol treatment may include, but are not limited to:

- Unacceptable toxicity
- The patient and/or parent/legal guardian withdraws consent for further treatment
- Patient becomes pregnant

Any patients who stop trial treatment prematurely will remain on the trial for follow-up unless the patients and/or parent/legal guardian explicitly withdraws consent for data collection.

If a patient discontinues treatment after administration of at least one dose of gemtuzumab ozogamicin, trial blood and tissue samples should still be collected and sent for analysis, unless the patient explicitly withdraws consent for further sample collection.

In the event of disease progression while on treatment, treatment should be continued until all doses are given in accordance with the protocol, unless the patient explicitly withdraws consent for further treatment.

8.2 Withdrawal of consent

The patient and/or parent/legal guardian may withdraw consent at any time during the trial. For the purposes of this trial, two types of withdrawal are defined:

- The patient would like to withdraw from trial medication, but is willing to be followed up according to the schedule of assessments (i.e. the patient has agreed that data and samples can be collected and used in the trial analysis)
- The patient would like to withdraw from trial medication or has completed trial medication and is not willing to be followed up for the purposes of the trial at any further visits (i.e. only data collected prior to the withdrawal of consent can be used in the trial analysis)

The details of the withdrawal (date, reason and type of withdrawal) should be clearly documented in the patient's medical records. A Withdrawal Form should be completed. A patient's wishes with respect to their data must be respected.

9. ADVERSE EVENT REPORTING

The collection and reporting of adverse events (AEs) will be in accordance with the Medicines for Human Use Clinical Trials Regulations 2004 and its subsequent amendments. Definitions of different types of AE are listed in Appendix 2. The Investigator should assess the seriousness and causality (relatedness) of all AEs experienced by the patient (this should be documented in the source data) with reference to the Summary of Product Characteristics (SmPC).

9.1 Reporting Requirements

9.1.1 Adverse Events (AE)

All medical occurrences which meet the definition of a grade 3 or above AE (see Appendix 3 for definition) should be reported. Please note this includes abnormal laboratory findings that meet the definition of CTCAE criteria. In addition, all SOS/VOD toxicity should be reported on a specific SOS/VOD AE form.

9.1.2 Serious Adverse Advents

Investigators should report AEs that meet the definition of an SAE (see Appendix 2 - Definition of Adverse Events for definition), or are listed in section 9.1.2.1.

9.1.2.1 Gemtuzumab ozogamicin serious adverse events of special interest

The following events are also required to be reported as a Serious Adverse Event (SAE):

- Hy's Law Cases, defined as:
    - ALT increased with abnormal elevations in total bilirubin and no other known cause of liver injury
      OR
    - AST increased with abnormal elevations in total bilirubin and no other known cause of liver injury

9.1.2.2 Events that do not require expedited reporting on a Serious Adverse Event Form

The following events should not be reported on an SAE Form:

- Hospitalisations for:
    - o Protocol defined treatment
    - o Pre-planned elective procedures unless the condition worsens
    - o Treatment for progression of the patient's disease
- Progression or death as a result of the patient's disease, as this information is captured elsewhere on the Case Report Form

9.1.2.3 Monitoring pregnancies for potential Serious Adverse Events

It is important to monitor the outcome of pregnancies of patients in order to provide SAE data on congenital anomalies or birth defects.

In the event that a patient or their partner becomes pregnant during the SAE reporting period please complete a Pregnancy Notification Form (providing the patient's details) and return to the Trials Office as soon as possible. If it is the patient who is pregnant provide outcome data on a follow-up Pregnancy Notification Form. Where the patient's partner is pregnant consent must first be obtained and the patient should be given a pregnancy Release of Information Form to give to their partner. If the partner is happy to provide information on the outcome of their pregnancy they should sign the pregnancy Release of Information Form. Once consent has been obtained provide details of the outcome of the pregnancy on a follow-up Pregnancy Notification Form. If appropriate also complete an SAE Form as detailed below.

9.1.3 Reporting period

Details of all reportable AEs will be documented and reported from the date of commencement of protocol defined treatment until 28 days after the administration of the last treatment.

9.1.3.1 Post trial Suspected Unexpected Serious Adverse Reactions (SUSARs)

SAEs that are judged to be at least possibly related to the IMP and are unexpected must still be reported in an expedited manner irrespective of how long after the IMP administration the reaction occurred.

9.2 Reporting Procedure

9.2.1 Site

9.2.1.1 Adverse Events

AEs should be reported on the Case Report Form (and where applicable) on an SAE Form. An AE Form should be completed at each visit and returned to the Trials Office.

AEs will be reviewed using the Common Terminology Criteria for Adverse Events (CTCAE), version 4.0 (see Appendix 3). Any AEs experienced by the patient but not included in the CTCAE should be graded by an Investigator and recorded on the AE Form using a scale of (1) mild, (2) moderate or (3) severe. For each sign/symptom, the highest grade observed since the last visit should be recorded.

9.2.1.2 Serious Adverse Events

For more detailed instructions on SAE reporting refer to the SAE Form Completion Guidelines contained in the Investigator Site File (ISF).

AEs which require reporting as an SAE should be reported on an SAE Form. When completing the form, the Investigator will be asked to define the causality and the severity of the AE which should be documented using the CTCAE version 4.0.

On becoming aware that a patient has experienced an SAE, the Investigator (or delegate) must complete, date and sign an SAE Form. The form should be emailed together with a SAE Cover Sheet to the Trials Office using one of the numbers listed below as soon as possible and no later than 24 hours after first becoming aware of the event:

To report an SAE

Email the report to:

reg@trials.bham.ac.uk

Include "Gotham SAE" in the subject line

On receipt the Trials Office will allocate each SAE a unique reference number. This reference number will be provided back to the site as proof of receipt. If confirmation of receipt is not received within 1 working day please contact the Trials Office. The SAE reference number should be quoted on all correspondence and follow-up reports regarding the SAE. The confirmation of receipt completed by the Trials Office should be filed with the SAE Form in the ISF.

For SAE Forms completed by someone other than the Investigator the Investigator will be required to countersign the original SAE Form to confirm agreement with the causality and severity assessments. The form should then be returned to the Trials Office in the post and a copy kept in the ISF.

Investigators should also report SAEs to their own Trust in accordance with local practice.

9.2.1.3 Provision of follow-up information

Patients should be followed up until resolution or stabilisation of the event. Follow-up information should be provided on a new SAE Form (refer to the SAE Form Completion Guidelines for further information).

9.2.2 Trials Office

On receipt of an SAE Form seriousness and causality will be determined independently by a Clinical Coordinator. An SAE judged by the Investigator or Clinical Coordinator to have a reasonable causal relationship with the trial medication will be regarded as a Serious Adverse Reaction (SAR). The Clinical Coordinator will also assess all SARs for expectedness. If the event meets the definition of a SAR that is unexpected (i.e. is not defined in the Reference Safety Information it will be classified as a Suspected Unexpected Serious Adverse Reaction (SUSAR).

9.2.3 Reporting to the Competent Authority and main Research Ethics Committee

9.2.3.1 Suspected Unexpected Serious Adverse Reactions

The Trials Office will report a minimal data set of all individual events categorised as a fatal or life threatening SUSAR to the Medicines and Healthcare products Regulatory Agency (MHRA) and main Research Ethics Committee (REC) within 7 days. Detailed follow-up information will be provided within an additional 8 days.

All other events categorised as SUSARs will be reported within 15 days.

9.2.3.2 Serious Adverse Reactions

The Trials Office will report details of all SARs (including SUSARs) to the MHRA and main REC annually from the date of the Clinical Trial Authorisation, in the form of an Annual Safety Report.

9.2.3.3 Adverse Events

Details of all AEs will be reported to the MHRA on request.

9.2.3.4 Other safety issues identified during the course of the trial

The MHRA and main REC will be notified immediately if a significant safety issue is identified during the course of the trial.

9.2.4 Investigators

Details of all SUSARs and any other safety issue which arises during the course of the trial will be reported to Principal Investigators. A copy of any such correspondence should be filed in the ISF.

9.2.5 Trial Steering Committee

The Trial Steering Committee (TSC) will review all SAEs.

10. DATA HANDLING AND RECORD KEEPING

10.1 Data Collection

This trial will utilise an eRDC system for completion of the CRF. Access to the eRDC system will be given to individuals via the Trials Office. The GOTHAM eRDC system can be accessed from:

http://www.cancertrials.bham.ac.uk/CRCTUPortal/GOTHAMLive

SAE reporting will be paper-based.

If the eRDC system is unavailable for an extended period of time a paper based CRF should be completed and forms returned to the Trials Office for data entry.

The CRF must be completed by an Investigator or an authorised member of the site research team (as delegated on the site signature and delegation log, or country specific equivalent) within the timeframe listed in the eRDC. The exceptions to this are the SAE form and Eligibility Checklists, which must be signed by an Investigator.

Entries on the paper CRF should be made in ballpoint pen, in blue or black ink, and must be legible. Any errors should be crossed out with a single stroke, the correction inserted and the change initialled and dated. If it is not obvious why a change has been made, an explanation should be written next to the change.

Data reported on each form should be consistent with the source data or the discrepancies should be explained. If information is not known, this must be indicated on the form. Missing and ambiguous data will be queried. All sections are to be completed before being submitted.

In all cases, it remains the responsibility of the Investigator to ensure that the CRF has been completed correctly and that the data are accurate.

The CRF may be amended by the Trials Office, as appropriate, throughout the duration of the trial. Whilst this will not constitute a protocol amendment, new versions of the form must be implemented by participating sites immediately on receipt, and acknowledgement of receipt and implementation should be sent to the Trials Office.

10.2 Archiving

It is the responsibility of the Principal Investigator to ensure all essential trial documentation and source records (e.g. signed Informed Consent Forms, Investigator Site Files, Pharmacy Files, patients' hospital notes, copies of CRFs etc.) at their site are securely retained for at least 25 years after the end of the trial. Do not destroy any documents without prior approval from the CRCTU Document Storage Manager.

11. QUALITY MANAGEMENT

11.1 Site Set-up and Initiation

All sites will be required to sign a Clinical Trial Site Agreement prior to participation. In addition all participating Investigators will be asked to sign the necessary agreements and supply a current CV to the Trials Office. All members of the site research team will also be required to sign the Site Signature and Delegation Log, which should be returned to the Trials Office. Prior to commencing recruitment all sites will undergo a process of initiation. Key members of the site research team will be required to attend either a meeting or a teleconference covering aspects of the trial design, protocol procedures, Adverse Event reporting, collection and reporting of data and record keeping. Sites will be provided with an Investigator Site File and a Pharmacy File containing essential documentation, instructions, and other documentation required for the conduct of the trial. The Trials Office must be informed immediately of any change in the site research team.

11.2 On-site Monitoring

Monitoring will be carried out as required following a risk assessment and as documented in the GOTHAM Quality Management Plan. Additional on-site monitoring visits may be triggered for example by poor CRF return, poor data quality, low SAE reporting rates, excessive number of patient withdrawals or deviations. If a monitoring visit is required the Trials Office will contact the site to arrange a date for the proposed visit and will provide the site with written confirmation. Investigators will allow the GOTHAM trial staff access to source documents as requested.

11.3 Central Monitoring

Where a patient has given explicit consent sites are requested to send in copies of signed Informed Consent Forms for in-house review.

Trials staff will be in regular contact with the site research team to check on progress and address any queries that they may have. Trials staff will check incoming Case Report Forms for compliance with the protocol, data consistency, missing data and timing. Sites will be sent Data Clarification Forms requesting missing data or clarification of inconsistencies or discrepancies.

Sites may be suspended from further recruitment in the event of serious and persistent non-compliance with the protocol and/or GCP, and/or poor recruitment. Any major problems identified during monitoring may be reported to Trial Management Group and the relevant regulatory bodies. This includes reporting serious breaches of GCP and/or the trial protocol to the main Research Ethics Committee (REC) and the Medicines for Healthcare products Regulatory Agency (MHRA).

11.4 Audit and Inspection

The Investigator will permit trial-related monitoring, audits, ethical review, and regulatory inspection(s) at their site, providing direct access to source data/documents.

Sites are also requested to notify the Trials Office of any MHRA inspections.

11.5 Notification of Serious Breaches

In accordance with Regulation 29A of the Medicines for Human Use (Clinical Trials) Regulations 2004 and its amendments the Sponsor of the trial is responsible for notifying the licensing authority in writing of any serious breach of:

- The conditions and principles of GCP in connection with that trial or;
- The protocol relating to that trial, within 7 days of becoming aware of that breach

For the purposes of this regulation, a “serious breach” is a breach which is likely to effect to a significant degree:

- The safety or physical or mental integrity of the subjects of the trial; or
- The scientific value of the trial

Sites are therefore requested to notify the Trials Office of a suspected trial-related serious breach of GCP and/or the trial protocol. Where the Trials Office is investigating whether or not a serious breach has occurred sites are also requested to cooperate with the Trials Office in providing sufficient information to report the breach to the MHRA where required and in undertaking any corrective and/or preventive action.

12. END OF TRIAL DEFINITION

The end of trial will be 12 months after the last data capture. This will allow sufficient time for the completion of protocol procedures, data collection and data input. The Trials Office will notify the MHRA and main REC that the trial has ended and will provide them with a summary of the clinical trial report within 6 months of the end of trial.

13. STATISTICAL CONSIDERATIONS

13.1 Definition of Outcome Measures

13.1.1 Primary outcome measures

- CD33+ cell count in the blood samples of patients collected at the pre-specified time points (day 1, day 8, day 15, day 22, day 29, day 43, day 50, and day 57).

13.1.2 Secondary outcome measures

- Overall survival (OS) time is defined as the time from the date of entry into the trial to the date of death. Patients lost to follow-up will be censored accordingly at date last seen.

- Progression free survival (PFS) time (Group 2 only) is defined as the time from the date of entry into the trial to the date of disease progression. Patients lost to follow-up will be censored accordingly at date last seen.
- Incidence of grade 3 and 4 adverse events is the frequency of adverse events as defined in section 9.

13.1.3 Exploratory Outcome Measures

- Change in IL-1/IL-6/ TNF-α in the plasma
- Change of CD33+ cells in the bone marrow/ tumour tissue (as available)

13.2 Analysis of Outcome Measures

Primary outcome measures

As an early phase II trial, the statistical analysis will essentially be descriptive, providing sufficient information to determine whether further research is warranted. The level of CD33+ will be repeatedly measured for each patient before, during and after treatment. For each patient this data will be tabulated and displayed graphically, visualising the change in CD33+ over time. Similarly, the mean CD33+ count at each time point will be plotted to show overall trend for both disease groups separately.

The mean/median relative change in CD33+, for each group, will be calculated and reported along with standard deviations/inter-quartile ranges where appropriate. This is the difference in CD33+ count from the initial blood samples, at either initial screening or day 1 of treatment, to the most recent.

The mean/median absolute change in CD33+, for each group, will also be calculated and reported along with standard deviations or inter-quartile ranges where appropriate. This is the biggest difference in CD33+ count from blood samples taken post day 1 of treatment.

The time to reach the lowest CD33+ cell count will also be reported. We will present the number and percentage of patients who reach their lowest CD33+ count, in each group, for each inter-dose day interval.

If appropriate, a more efficient Bayesian repeated measures analysis will be performed using flexible mixed effects models accounting for subject correlation to assess changes over time in CD33+ count.

Secondary outcome measures

OS (for each group separately) and PFS time (for group 2 only) will be assessed using the method of Kaplan and Meier with point estimates presented at 3, 6, 9 and 12 months along with median survival time.

Incidence of grade 3 and 4 adverse events will be assessed for each group. A summary of the number of events and patients for all toxicities split by event and grade will be reported. The maximum grade experienced for all patients will also be reported.

13.3 Planned Interim Analysis

The trial is expected to recruit over two years. There is no formal interim statistical analysis embedded into the design but an interim analysis of the data will be presented to an independent Trial Steering Committee (TSC) after first 3 patients have received treatment and reach day 57 for them to review the safety and feasibility of the trial. The next interim analysis of the data will be presented to the TSC after another 3 patients have received treatment and reach day 57.

13.4 Planned Final Analyses

The main analysis of the primary outcome will be performed separately for each group, once all patients in their respective group have reached the final sample collection time point (day 57 after final dose sample.

The main analysis of the secondary outcomes will be performed once each patient has been followed up for a minimum of 1 year after the end of trial treatment. A full end of trial report will be produced at this time.

13.5 Sample Size and Power Calculations

No statistical testing will take place as such there is no target sample size. A minimum of 10 patients per group will be recruited. If any Bayesian modelling is to be performed simulations will be run to determine effective sample sizes and the protocol and SAP will be updated accordingly.

14. TRIAL ORGANISATIONAL STRUCTURE

14.1 Sponsor

The University of Birmingham is the Sponsor.

14.2 Coordinating Centre

The trial is being conducted under the auspices of the Cancer Research UK Clinical Trials Unit (CRCTU), University of Birmingham according to their local procedures.

14.3 Trial Management Group

The Trial Management Group (TMG) is composed of the Chief Investigator, co-investigators, laboratory lead and representatives from the trial team at the CRCTU. The TMG is responsible for the day-to-day running and management of the trial and will meet by teleconference or in person approximately every 3 months.

14.4 Trial Steering Committee

Data analyses will be supplied in confidence to the Trial Steering Committee (TSC) which will have an independent chair. The TSC will be asked to give advice on whether the accumulated data from the trial, together with the results from other relevant research, justifies the continuing recruitment of further patients. During the recruitment phase of the trial the TSC is scheduled to meet after 3 patient have completed treatment and 6 monthly thereafter. Additional meetings may be called if recruitment is much faster than anticipated and the TSC may, at their discretion, request to meet more frequently or continue to meet following completion of recruitment. An emergency meeting may also be convened if a safety issue is identified.

The TSC will report directly to the Trial Management Group (TMG) who will convey the findings to the Sponsor, MHRA, REC or funder if applicable. The TSC may consider recommending the discontinuation of the trial if the recruitment rate or data quality are unacceptable or if any issues are identified which may compromise patient safety.

14.5 Data Monitoring Committee

Given the small size of the study, a formal data monitoring committee will not be established. Oversight of the study will be maintained by the Trial Steering Committee

14.6 Finance

This is a clinician-initiated and clinician-led trial funded by The Little Princess Trust, The Rosetrees Trust and the Eveson Charitable Trust.

No individual per patient payment will be made to, Investigators or patients. Sites will be compensated for their research activities carried out in relation to the trial as defined in the Clinical Trial Site Agreement. Pfizer are providing the IMP to the sites.

This trial has been adopted into the NIHR CRN Portfolio.

15. ETHICAL CONSIDERATIONS

The trial will be performed in accordance with the recommendations guiding physicians in biomedical research involving human subjects, adopted by the 18th World Medical Association General Assembly, Helsinki, Finland, June 1964, amended at the 48th World Medical Association General Assembly, Somerset West, Republic of South Africa, October 1996 (website: http://www.wma.net/en/30publications/10policies/b3/index.html).

The trial will be conducted in accordance with the Research Governance Framework for Health and Social Care, the applicable UK Statutory Instruments, (which include the Medicines for Human Use Clinical Trials 2004 and subsequent amendments and the General Data Protection Regulation and Human Tissue Act 2008) and Good Clinical Practice (GCP). This trial will be carried out under a Clinical Trial Authorisation in accordance with the Medicines for Human Use Clinical Trials regulations. The protocol will be submitted to and approved by the main Research Ethics Committee (REC) prior to circulation.

Before any patients are enrolled into the trial, the Principal Investigator at each site is required to obtain local R&D approval. Sites will not be permitted to enrol patients until written confirmation of R&D approval is received by the Trials Office.

It is the responsibility of the Principal Investigator to ensure that all subsequent amendments gain the necessary local approval. This does not affect the individual clinicians' responsibility to take immediate action if thought necessary to protect the health and interest of individual patients.

16. CONFIDENTIALITY AND DATA PROTECTION

Personal data recorded on all documents will be regarded as strictly confidential and will be handled and stored in accordance with the General Data Protection Regulation and the Data Protection Act 2018. With the patient's consent, their initials and date of birth will be collected at trial entry.

Patients will be identified using only their unique trial number on the CRF/eRDC and in correspondence between the Trials Office and the participating site. Any laboratory samples will be labelled with the patient's unique trial number, initials and date of birth to ensure that samples can be correctly identified. Pathology number may also be collected. National Health Service (NHS) number will be collected where the patients consent. Patients are asked to give permission for the Trials Office to be sent a copy of their signed Informed Consent Form which will not be anonymised. This will be used to perform in-house monitoring of the consent process.

The Investigator must maintain documents not for submission to the Trials Office (e.g. Patient Identification Logs) in strict confidence. In the case of specific issues and/or queries from the regulatory authorities, it will be necessary to have access to the complete trial records, provided that patient confidentiality is protected.

The Trials Office will maintain the confidentiality of all patient's data and will not disclose information by which patients may be identified to any third party other than those directly involved in the treatment of the patient and organisations for which the patient has given explicit consent for data transfer. Representatives of the GOTHAM trial team may be required to have access to patient's notes for quality assurance purposes but patients should be reassured that their confidentiality will be respected at all times.

17. INSURANCE AND INDEMNITY

University of Birmingham employees are indemnified by the University insurers for negligent harm caused by the design or co-ordination of the clinical trials they undertake whilst in the University's employment.

In terms of liability at a site, NHS Trust and non-Trust hospitals have a duty to care for patients treated, whether or not the patient is taking part in a clinical trial. Compensation is therefore available via NHS indemnity in the event of clinical negligence having been proven.

The University of Birmingham cannot offer indemnity for non-negligent harm. The University of Birmingham is independent of any pharmaceutical company, and as such it is not covered by the Association of the British Pharmaceutical Industry (ABPI) guidelines for patient compensation.

18. PUBLICATION POLICY

Results of this trial will be submitted for publication in a peer reviewed journal. The manuscript will be prepared by the Trial Management Group (TMG) and authorship will be determined by mutual agreement.

Any secondary publications and presentations prepared by Investigators must be reviewed by the TMG. Manuscripts must be submitted to the TMG in a timely fashion and in advance of being submitted for publication, to allow time for review and resolution of any outstanding issues. Authors must acknowledge that the trial was performed with the support of the University of Birmingham and funding bodies. Intellectual property rights will be addressed in the Clinical Trial Site Agreement between Sponsor and site.

19. REFERENCE LIST

1. Yang, M., et al., *Diverse Functions of Macrophages in Different Tumor Microenvironments.* Cancer Res, 2018. **78**(19): p. 5492-5503.
2. Park, J.H., et al., *Long-Term Follow-up of CD19 CAR Therapy in Acute Lymphoblastic Leukemia.* N Engl J Med, 2018. **378**(5): p. 449-459.
3. Louis, C.U., et al., *Antitumor activity and long-term fate of chimeric antigen receptor-positive T cells in patients with neuroblastoma.* Blood, 2011. **118**(23): p. 6050-6.
4. Khanna, S., et al., *Tumor-Derived GM-CSF Promotes Granulocyte Immunosuppression in Mesothelioma Patients.* Clin Cancer Res, 2018. **24**(12): p. 2859-2872.
5. Mussai, F., C. De Santo, and V. Cerundolo, *Interaction between invariant NKT cells and myeloid-derived suppressor cells in cancer patients: evidence and therapeutic opportunities.* J Immunother, 2012. **35**(6): p. 449-59.
6. Bronte, V., et al., *Recommendations for myeloid-derived suppressor cell nomenclature and characterization standards.* Nat Commun, 2016. **7**: p. 12150.
7. Mantovani, A., et al., *Tumour-associated macrophages as treatment targets in oncology.* Nature Reviews Clinical Oncology, 2017. **14**(7): p. 399-416.
8. Tobin, R.P., et al., *The clinical evidence for targeting human myeloid-derived suppressor cells in cancer patients.* J Leukoc Biol, 2017. **102**(2): p. 381-391.
9. Bracaglia, C., G. Prencipe, and F. De Benedetti, *Macrophage Activation Syndrome: different mechanisms leading to a one clinical syndrome.* Pediatr Rheumatol Online J, 2017. **15**(1): p. 5.
10. Daver, N., et al., *A consensus review on malignancy-associated hemophagocytic lymphohistiocytosis in adults.* Cancer, 2017. **123**(17): p. 3229-3240.
11. Schulert, G.S. and A.A. Grom, *Pathogenesis of macrophage activation syndrome and potential for cytokine- directed therapies.* Annu Rev Med, 2015. **66**: p. 145-59.
12. Marsh, R.A., et al., *Salvage therapy for refractory hemophagocytic lymphohistiocytosis: A review of the published experience.* Pediatr Blood Cancer, 2017. **64**(4).
13. Dunleavy, K., et al., *Dose-adjusted EPOCH-R (etoposide, prednisone, vincristine, cyclophosphamide, doxorubicin, and rituximab) in untreated aggressive diffuse large B-cell lymphoma with MYC rearrangement: a prospective, multicentre, single-arm phase 2 study.* Lancet Haematol, 2018. **5**(12): p. e609-e617.
14. Antonia, S.J., et al., *Overall Survival with Durvalumab after Chemoradiotherapy in Stage III NSCLC.* New England Journal of Medicine, 2018. **379**(24): p. 2342-2350.
15. Horwitz, S., et al., *Brentuximab vedotin with chemotherapy for CD30-positive peripheral T-cell lymphoma (ECHELON-2): a global, double-blind, randomised, phase 3 trial.* Lancet, 2019. **393**(10168): p. 229-240.
16. Mussai, F., et al., *Cytotoxicity of the anti-CD22 immunotoxin HA22 (CAT-8015) against paediatric acute lymphoblastic leukaemia.* Br J Haematol, 2010. **150**(3): p. 352-8.
17. Viardot, A., et al., *Phase 2 study of the bispecific T-cell engager (BiTE) antibody blinatumomab in relapsed/refractory diffuse large B-cell lymphoma.* Blood, 2016. **127**(11): p. 1410-6.
18. Maude, S.L., et al., *Tisagenlecleucel in Children and Young Adults with B-Cell Lymphoblastic Leukemia.* N Engl J Med, 2018. **378**(5): p. 439-448.
19. Lemos, H., et al., *Immune control by amino acid catabolism during tumorigenesis and therapy.* Nat Rev Cancer, 2019.
20. Gabrilovich, D.I., S. Ostrand-Rosenberg, and V. Bronte, *Coordinated regulation of myeloid cells by tumours.* Nat Rev Immunol, 2012. **12**(4): p. 253-68.
21. Mussai, F., et al., *Targeting the arginine metabolic brake enhances immunotherapy for leukaemia.* Int J Cancer, 2018.
22. De Santo, C., et al., *Invariant NKT cells modulate the suppressive activity of IL-10-secreting neutrophils differentiated with serum amyloid A.* Nat Immunol, 2010. **11**(11): p. 1039-46.

23. Fultang, L., et al., *Macrophage-Derived IL1beta and TNFalpha Regulate Arginine Metabolism in Neuroblastoma.* Cancer Res, 2019. **79**(3): p. 611-624.

24. Marsh, R.A. and E. Haddad, *How i treat primary haemophagocytic lymphohistiocytosis.* British Journal of Haematology, 2018. **182**(2): p. 185-199.

25. Neelapu, S.S., et al., *Chimeric antigen receptor T-cell therapy - assessment and management of toxicities.* Nat Rev Clin Oncol, 2018. **15**(1): p. 47-62.

26. Teachey, D.T., et al., *Cytokine release syndrome after blinatumomab treatment related to abnormal macrophage activation and ameliorated with cytokine-directed therapy.* Blood, 2013. **121**(26): p. 5154-7.

27. De Santo, C., et al., *Nitroaspirin corrects immune dysfunction in tumor-bearing hosts and promotes tumor eradication by cancer vaccination.* Proc Natl Acad Sci U S A, 2005. **102**(11): p. 4185-90.

28. Daley, J.M., et al., *Use of Ly6G-specific monoclonal antibody to deplete neutrophils in mice.* Journal of Leukocyte Biology, 2008. **83**(1): p. 64-70.

29. Schumak, B., et al., *Specific Depletion of Ly6C(hi) Inflammatory Monocytes Prevents Immunopathology in Experimental Cerebral Malaria.* Plos One, 2015. **10**(4).

30. Crocker, P.R., J.C. Paulson, and A. Varki, *Siglecs and their roles in the immune system.* Nature Reviews Immunology, 2007. **7**(4): p. 255-266.

31. McMillan, S.J. and P.R. Crocker, *CD33-related sialic-acid-binding immunoglobulin-like lectins in health and disease.* Carbohydr Res, 2008. **343**(12): p. 2050-6.

32. Gustafson, M.P., et al., *A method for identification and analysis of non-overlapping myeloid immunophenotypes in humans.* PLoS One, 2015. **10**(3): p. e0121546.

33. Kossman, S.E., et al., *A phase I trial of humanized monoclonal antibody HuM195 (anti-CD33) with low-dose interleukin 2 in acute myelogenous leukemia.* Clin Cancer Res, 1999. **5**(10): p. 2748-55.

34. Sutherland, M., et al., *SGN-33 (Lintuzumab), a humanized anti-CD33 antibody, modulates the activity of CD33⁺ tumor-associated macrophages.* Cancer Research, 2007. **67**(9 Supplement): p. 4111-4111.

35. Sievers, E.L., et al., *Selective ablation of acute myeloid leukemia using antibody-targeted chemotherapy: A phase I study of an anti-CD33 calicheamicin immunoconjugate.* Blood, 1999. **93**(11): p. 3678-3684.

36. Fultang, L., et al., *MDSC targeting with Gemtuzumab ozogamicin restores T cell immunity and immunotherapy against cancers.* EBioMedicine, 2019. **47**: p. 235-246.

37. van Der Velden, V.H., et al., *Targeting of the CD33-calicheamicin immunoconjugate Mylotarg (CMA-676) in acute myeloid leukemia: in vivo and in vitro saturation and internalization by leukemic and normal myeloid cells.* Blood, 2001. **97**(10): p. 3197-204.

38. Wunderlich, M., et al., *A xenograft model of macrophage activation syndrome amenable to anti-CD33 and anti-IL-6R treatment.* JCI Insight, 2016. **1**(15): p. e88181.

39. Pollard, J.A., et al., *CD33 Expression and Its Association With Gemtuzumab Ozogamicin Response: Results From the Randomized Phase III Children's Oncology Group Trial AAML0531.* J Clin Oncol, 2016. **34**(7): p. 747-55.

40. Zahler, S., et al., *A Phase I Study of Reduced-Intensity Conditioning and Allogeneic Stem Cell Transplantation Followed by Dose Escalation of Targeted Consolidation Immunotherapy with Gemtuzumab Ozogamicin in Children and Adolescents with CD33+ Acute Myeloid Leukemia.* Biol Blood Marrow Transplant, 2016. **22**(4): p. 698-704.

41. Tarlock, K., et al., *Gemtuzumab Ozogamicin Reduces Relapse Risk in FLT3/ITD Acute Myeloid Leukemia: A Report from the Children's Oncology Group.* Clin Cancer Res, 2016. **22**(8): p. 1951-7.

42. Satwani, P., et al., *A Phase I study of gemtuzumab ozogamicin (GO) in combination with busulfan and cyclophosphamide (Bu/Cy) and allogeneic stem cell transplantation in children with poor-risk CD33+ AML: a new targeted immunochemotherapy myeloablative conditioning (MAC) regimen.* Biol Blood Marrow Transplant, 2012. **18**(2): p. 324-9.

43. Arceci, R.J., et al., *Safety and efficacy of gemtuzumab ozogamicin in pediatric patients with advanced CD33+ acute myeloid leukemia.* Blood, 2005. **106**(4): p. 1183-8.

44. Gibson, B., et al., *Intensified Dosing of Gemtuzumab Ozogamicin Can be Safely Combined with Induction Chemotherapy in Children with Acute Myeloid Leukaemia (AML) and High Risk Myelodysplasia (MDS).* Blood, 2018. **132**(Supplement 1): p. 2681-2681.

45. Buckwalter, M., et al., *Pharmacokinetics of gemtuzumab ozogamicin as a single-agent treatment of pediatric patients with refractory or relapsed acute myeloid leukemia.* Journal of Clinical Pharmacology, 2004. **44**(8): p. 873-880.

46. Dowell, J.A., et al., *Pharmacokinetics of gemtuzumab ozogamicin, an antibody-targeted chemotherapy agent for the treatment of patients with acute myeloid leukemia in first relapse.* Journal of Clinical Pharmacology, 2001. **41**(11): p. 1206-1214.

47. Lambert, J., et al., *Gemtuzumab ozogamicin for de novo acute myeloid leukemia: final efficacy and safety updates from the open-label, phase III ALFA-0701 trial.* Haematologica, 2019. **104**(1): p. 113-119.

48. Gamis, A.S., et al., *Gemtuzumab ozogamicin in children and adolescents with de novo acute myeloid leukemia improves event-free survival by reducing relapse risk: results from the randomized phase III Children's Oncology Group trial AAML0531.* J Clin Oncol, 2014. **32**(27): p. 3021-32.

49. Henter, J.I., et al., *HLH-2004: Diagnostic and therapeutic guidelines for hemophagocytic lymphohistiocytosis.* Pediatr Blood Cancer, 2007. **48**(2): p. 124-31.

50. La Rosée, P., et al., *Recommendations for the management of hemophagocytic lymphohistiocytosis in adults.* Blood, 2019. **133**(23): p. 2465-2477.

51. Ravelli, A., et al., *2016 Classification Criteria for Macrophage Activation Syndrome Complicating Systemic Juvenile Idiopathic Arthritis: A European League Against Rheumatism/American College of Rheumatology/Paediatric Rheumatology International Trials Organisation Collaborative Initiative.* Arthritis Rheumatol, 2016. **68**(3): p. 566-76.

52. Lerkvaleekul, B. and S. Vilaiyuk, *Macrophage activation syndrome: early diagnosis is key.* Open Access Rheumatol, 2018. **10**: p. 117-128.

53. Cui, Y., et al., *High-Volume Hemofiltration in Critically Ill Patients With Secondary Hemophagocytic Lymphohistiocytosis/Macrophage Activation Syndrome: A Prospective Study in the PICU.* Pediatr Crit Care Med, 2016. **17**(10): p. e437-e443.

54. Shakoory, B., et al., *Interleukin-1 Receptor Blockade Is Associated With Reduced Mortality in Sepsis Patients With Features of Macrophage Activation Syndrome: Reanalysis of a Prior Phase III Trial.* Crit Care Med, 2016. **44**(2): p. 275-81.

55. Kyriazopoulou, E., et al., *Macrophage activation-like syndrome: an immunological entity associated with rapid progression to death in sepsis.* BMC Med, 2017. **15**(1): p. 172.

56. Lehmberg, K., et al., *Malignancy-associated haemophagocytic lymphohistiocytosis in children and adolescents.* Br J Haematol, 2015. **170**(4): p. 539-49.

57. Wang, Y.N., et al., *Multicenter study of combination DEP regimen as a salvage therapy for adult refractory hemophagocytic lymphohistiocytosis.* Blood, 2015. **126**(19): p. 2186-2192.

APPENDIX 1 - WMA DECLARATION OF HELSINKI

WORLD MEDICAL ASSOCIATION DECLARATION OF HELSINKI
Recommendations guiding physicians
in biomedical research involving human subjects
Adopted by the 18th World Medical Assembly
Helsinki, Finland, June 1964
and amended by the
29th World Medical Assembly, Tokyo, Japan, October 1975
35th World Medical Assembly, Venice, Italy, October 1983
41st World Medical Assembly, Hong Kong, September 1989
and the
48th General Assembly, Somerset West, Republic of South Africa, October 1996

INTRODUCTION

It is the mission of the physician to safeguard the health of the people. His or her knowledge and conscience are dedicated to the fulfillment of this mission.

The Declaration of Geneva of the World Medical Association binds the physician with the words, "The Health of my patient will be my first consideration," and the International Code of Medical Ethics declares that, "A physician shall act only in the patient's interest when providing medical care which might have the effect of weakening the physical and mental condition of the patient."

The purpose of biomedical research involving human subjects must be to improve diagnostic, therapeutic and prophylactic procedures and the understanding of the aetiology and pathogenesis of disease.

In current medical practice most diagnostic, therapeutic or prophylactic procedures involve hazards. This applies especially to biomedical research.

Medical progress is based on research which ultimately must rest in part on experimentation involving human subjects.

In the field of biomedical research a fundamental distinction must be recognised between medical research in which the aim is essentially diagnostic or therapeutic for a patient, and medical research, the essential object of which is purely scientific and without implying direct diagnostic or therapeutic value to the person subjected to the research.

Special caution must be exercised in the conduct of research which may affect the environment, and the welfare of animals used for research must be respected.

Because it is essential that the results of laboratory experiments be applied to human beings to further scientific knowledge and to help suffering humanity, the World Medical Association has prepared the following recommendations as a guide to every physician in biomedical research involving human subjects. They should be kept under review in the future. It must be stressed that the standards as drafted are only a guide to physicians all over the world. Physicians are not relieved from criminal, civil and ethical responsibilities under the laws of their own countries.

I. BASIC PRINCIPLES

2. Biomedical research involving human subjects must conform to generally accepted scientific principles and should be based on adequately performed laboratory and animal experimentation and on a thorough knowledge of the scientific literature.

3. The design and performance of each experimental procedure involving human subjects should be clearly formulated in an experimental protocol which should be transmitted for consideration, comment and guidance to a specially appointed committee independent of the investigator and the

sponsor provided that this independent committee is in conformity with the laws and regulations of the country in which the research experiment is performed.

4. Biomedical research involving human subjects should be conducted only by scientifically qualified persons and under the supervision of a clinically competent medical person. The responsibility for the human subject must always rest with a medically qualified person and never rest on the subject of the research, even though the subject has given his or her consent.

5. 4. Biomedical research involving human subjects cannot legitimately be carried out unless the importance of the objective is in proportion to the inherent risk to the subject.

6. Every biomedical research project involving human subjects should be preceded by careful assessment of predictable risks in comparison with foreseeable benefits to the subject or to others. Concern for the interests of the subject must always prevail over the interests of science and society.

7. The right of the research subject to safeguard his or her integrity must always be respected. Every precaution should be taken to respect the privacy of the subject and to minimise the impact of the study on the subject's physical and mental integrity and on the personality of the subject.

8. Physicians should abstain from engaging in research projects involving human subjects unless they are satisfied that the hazards involved are believed to be predictable. Physicians should cease any investigation if the hazards are found to outweigh the potential benefits.

9. In publication of the results of his or her research, the physician is obliged to preserve the accuracy of the results. Reports of experimentation not in accordance with the principles laid down in this Declaration should not be accepted for publication.

10. In any research on human beings, each potential subject must be adequately informed of the aims, methods, anticipated benefits and potential hazards of the study and the discomfort it may entail. He or she should be informed that he or she is at liberty to abstain from participation in the study and that he or she is free to withdraw his or her consent to participation at any time. The physician should then obtain the subject's freely-given informed consent, preferably in writing.

11. When obtaining informed consent for the research project the physician should be particularly cautious if the subject is in a dependent relationship to him or her or may consent under duress. In that case the informed consent should be obtained by a physician who is not engaged in the investigation and who is completely independent of this official relationship.

12. In case of legal incompetence, informed consent should be obtained from the legal guardian in accordance with national legislation. Where physical or mental incapacity makes it impossible to obtain informed consent, or when the subject is a minor, permission from the responsible relative replaces that of the subject in accordance with national legislation. Whenever the minor child is in fact able to give a consent, the minor's consent must be obtained in addition to the consent of the minor's legal guardian.

13. The research protocol should always contain a statement of the ethical considerations involved and should indicate that the principles enunciated in the present Declaration are complied with.

II. MEDICAL RESEARCH COMBINED WITH PROFESSIONAL CARE (Clinical Research)

1. In the treatment of the sick person, the physician must be free to use a new diagnostic and therapeutic measure, if in his or her judgement it offers hope of saving life, reestablishing health or alleviating suffering.

2. The potential benefits, hazards and discomfort of a new method should be weighed against the advantages of the best current diagnostic and therapeutic methods.

3. In any medical study, every patient - including those of a control group, if any - should be assured of the best proven diagnostic and therapeutic method. This does not exclude the use of inert placebo in studies where no proven diagnostic or therapeutic method exists.

4. The refusal of the patient to participate in a study must never interfere with the physician-patient relationship.

5. If the physician considers it essential not to obtain informed consent, the specific reasons for this proposal should be stated in the experimental protocol for transmission to the independent committee (I, 2).

6. The physician can combine medical research with professional care, the objective being the acquisition of new medical knowledge, only to the extent that medical research is justified by its potential diagnostic or therapeutic value for the patient.

III. NON-THERAPEUTIC BIOMEDICAL RESEARCH INVOLVING HUMAN SUBJECTS (Non-Clinical Biomedical Research)

7. In the purely scientific application of medical research carried out on a human being, it is the duty of the physician to remain the protector of the life and health of that person on whom biomedical research is being carried out.

8. The subject should be volunteers - either healthy persons or patients for whom the experimental design is not related to the patient's illness.

9. The investigator or the investigating team should discontinue the research if in his/her or their judgement it may, if continued, be harmful to the individual.

10. In research on man, the interest of science and society should never take precedence over considerations related to the wellbeing of the subject.

APPENDIX 2 - DEFINITION OF ADVERSE EVENTS

Adverse Event

Any untoward medical occurrence in a patient or clinical trial subject administered a medicinal product and which does not necessarily have a causal relationship with this treatment.

Comment:

An AE can therefore be any unfavourable and unintended sign (including abnormal laboratory findings), symptom or disease temporally associated with the use of an investigational medicinal product, whether or not related to the investigational medicinal product.

Adverse Reaction

All untoward and unintended responses to an IMP related to any dose administered.

Comment:

An AE judged by either the reporting Investigator or Sponsor as having causal relationship to the IMP qualifies as an AR. The expression reasonable causal relationship means to convey in general that there is evidence or argument to suggest a causal relationship.

Serious Adverse Event

Any untoward medical occurrence or effect that at any dose:

- Results in death
- Is life-threatening*
- Requires hospitalisation** or prolongation of existing inpatients' hospitalisation
- Results in persistent or significant disability or incapacity
- Is a congenital anomaly/birth defect
- Or is otherwise considered medically significant by the Investigator***

Comments:

The term severe is often used to describe the intensity (severity) of a specific event. This is not the same as serious, which is based on patients/event outcome or action criteria.

* Life threatening in the definition of an SAE refers to an event in which the patient was at risk of death at the time of the event; it does not refer to an event that hypothetically might have caused death if it were more severe.

**Hospitalisation is defined as an unplanned, formal inpatient admission, even if the hospitalisation is a precautionary measure for continued observation. Thus hospitalisation for protocol treatment (e.g. line insertion), elective procedures (unless brought forward because of worsening symptoms) or for social reasons (e.g. respite care) are not regarded as an SAE.

*** Medical judgment should be exercised in deciding whether an AE is serious in other situations. Important AEs that are not immediately life threatening or do not result in death or hospitalisation but may jeopardise the subject or may require intervention to prevent one of the other outcomes listed in the definition above, should be considered serious.

Serious Adverse Reaction

An Adverse Reaction which also meets the definition of a Serious Adverse Event.

Suspected Unexpected Serious Adverse Reaction

A SAR that is unexpected i.e. the nature, or severity of the event is not consistent with the applicable product information.

A SUSAR should meet the definition of an AR, UAR and SAR.

Unexpected Adverse Reaction

An AR, the nature or severity of which is not consistent with the applicable product information (e.g. Investigator Brochure for an unapproved IMP or (compendium of) Summary of Product Characteristics (SPC) for a licensed product).

When the outcome of an AR is not consistent with the applicable product information the AR should be considered unexpected.

APPENDIX 3 - COMMON TOXICITY CRITERIA GRADINGS

Toxicities will be recorded according to the Common Terminology Criteria for Adverse Events (CTCAE), version 4.0. The full CTCAE document is available on the National Cancer Institute (NCI) website.

APPENDIX 4 – HLH DIAGNOSTIC CRITERIA

The diagnosis of HLH can be established if Criterion 1 or 2 is fulfilled.
1. A molecular diagnosis consistent with HLH
2. Diagnostic criteria for HLH fulfilled (5 of the 8 criteria below)<br>Fever<br>Splenomegaly<br>Cytopenias (affecting ≥2 of 3 lineages in the peripheral blood)<br>Hemoglobin <90 g/L (hemoglobin <100 g/L in infants <4 wk)<br>Platelets <100 × 10^9/L<br>Neutrophils <1.0 × 10^9/L<br>Hypertriglyceridemia and/or hypofibrinogenemia<br>Fasting triglycerides ≥3.0 mmol/L (ie, ≥265 mg/dL)<br>Fibrinogen ≤1.5 g/L<br>Hemophagocytosis in bone marrow or spleen or lymph nodes. No evidence of malignancy.<br>Low or no NK cell activity (according to local laboratory reference)<br>Ferritin ≥500 μg/L<br>sCD25 (ie, soluble IL-2 receptor) ≥2400 U/mL

If hemophagocytic activity is not proven at the time of presentation, further search for hemophagocytic activity is encouraged. If the bone marrow specimen is not conclusive, material may be obtained from other organs. Serial marrow aspirates over time may also be helpful. The following findings may provide strong supportive evidence for the diagnosis: spinal fluid pleocytosis (mononuclear cells) and/or elevated spinal fluid protein and histological picture in the liver resembling chronic persistent hepatitis (biopsy). Other abnormal clinical and laboratory findings consistent with the diagnosis are cerebromeningeal symptoms, lymph node enlargement, jaundice, edema, skin rash, hepatic enzyme abnormalities, hypoproteinemia, hyponatremia, and elevated very lowdensity lipoprotein (VLDL↑)/low high-density lipoprotein (HDL↓).

Appendix 2. GOTHAM trial statistical analysis plan

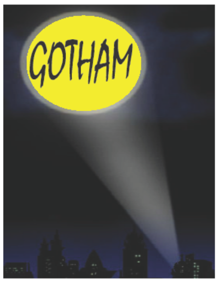

Statistical Analysis Plan

A phase II trial to assess the activity of Gemtuzumab Ozogamicin Therapy in HAemophagocytic lymphohistiocytosis (HLH) or Macrophage activation syndrom (MAS) or relapsed/refractory solid tumours

Version: 2.0
January 17, 2023

Sponsor(s): University of Birmingham
EudraCT Number: 2020-002428-36
Sponsor Protocol Number: RG_19-271
CAS Number: HC2001

Author(s):

**Name:**	Amit Patel
**Trial role:**	Trial Statistician

Reviewed & Approved by:

**Name:**	Professor Gary Middleton
**Trial role:**	Chief Investigator

CANCER RESEARCH UK | BIRMINGHAM CANCER RESEARCH UK CLINICAL TRIALS UNIT | UNIVERSITY OF BIRMINGHAM

Key personnel involved in the Statistical Analysis Plan:

Name	Trial role
Professor Gary Middleton	Chief Investigator
Professor Lucinda Billingham	Professor of Biostatistics
Amit Patel	Trial Statistician

Document Control Sheet:

Statistical Analysis Plan version:	Reason for update:
Vn1.0, Vd04-Mar-2021	Creation from Protocol version 1.0 dated 10th June 2020.
Vn2.0, Vd17-Jan-2023	A change was made to the treatment schedule.

Contents

1 INTRODUCTION 4
1.1 Purpose of the Statistical Analysis Plan . . . 4
1.2 Summary of the Trial . . . 4

2 TIMING AND REPORTING OF INTERIM AND FINAL ANALYSES 5

3 RECRUITMENT AND RANDOMISATION 5
3.1 Recruitment . . . 5
3.2 Randomisation . . . 6
3.3 Ineligible Patients . . . 6

4 DATA QUALITY 6
4.1 Return Rate for each CRF . . . 6
4.2 Length of Patient Follow-Up . . . 6

5 TRIAL POPULATION 6
5.1 Baseline Patient Characteristics . . . 6
5.2 Definition of Populations for Analysis . . . 6

6 TREATMENT RECEIVED 6

7 ADVERSE EVENT REPORTING 7

8 ANALYSIS 7
8.1 Definition and Calculation of Outcome Measures and Descriptive Analyses . . . 7
8.1.1 Primary Objective(s) . . . 7
8.1.2 Secondary Outcome Measures(s) . . . 8
8.2 Sample Size Calculations . . . 8
8.3 Subgroup Analysis . . . 9

9 STATISTICAL SOFTWARE 9

10 STORAGE AND ARCHIVING 9

1 INTRODUCTION

1.1 Purpose of the Statistical Analysis Plan

This Statistical Analysis Plan (SAP) provides guidelines for the analysis and presentation of results for the GOTHAM trial. This plan, along with all other documents relating to the analysis of this trial, will be stored in the 'Statistical Documentation' section of the Trial Master File. The statistical analysis will be carried out by the Trial Statistician in collaboration with the Lead Statistician.

1.2 Summary of the Trial

Trial Design
GOTHAM is a single arm, open label phase II trial in patients with Haemophagocytic Lymphohistiocytosis (HLH) or Macrophage activation syndrome (MAS) (Group 1) or patients with relapsed/refractory (R/R) solid tumours (Group 2).

Primary Objective(s)

- To assess the activity of gemtuzumab ozogamicin (GO) 3 mg/m_2/dose on days 1, 22, 43 by measuring the change in CD33+ myeloid cells in the blood in 2 parallel groups: patients with HLH/MAS (Group 1) or R/R solid tumours (Group 2).

Secondary Objectives

- To assess the effect of gemtuzumab ozogamicin on overall survival time (OS) and progression free survival time (PFS) (Group 2 only).
- To assess the feasibility of delivering gemtuzumab ozogamicin in the HLH/MAS (Group 1) and R/R solid tumours (Group 2)

Exploratory Research Objectives

- To assess the change in IL-1/IL-6/TNF-α in the plasma
- To assess the change of CD33+ cells in the bone marrow/tumour tissue (as available)

Primary Outcome Measure(s)

- CD33+ cell count in the blood samples of patients collected at the pre-specified time points (day 1, day 8, day 15, day 22, day 43, day 50, and day 57).

Secondary Outcome Measures

- Overall survival time
- Progression free survival time (Group 2 only)
- Incidence of grade 3 and 4 adverse events

Exploratory Research Outcomes

- Change in IL-1/IL-6/TNF-α in the plasma
- Change of CD33+ cells in the bone marrow/tumour tissue (as available)

Patient Population
Group 1: Patients with Haemophagocytic Lymphohistiocytosis (HLH) or Macrophage activation syndrome (MAS)
Group 2: Patients with relapsed/refractory solid tumours

Sample Size
A total of 20 evaluable patients: 10 patients per group

Trial Duration
The trial will recruit for approximately 2 years, and all patients will be followed up for a minimum of 1 year.

Trial schema

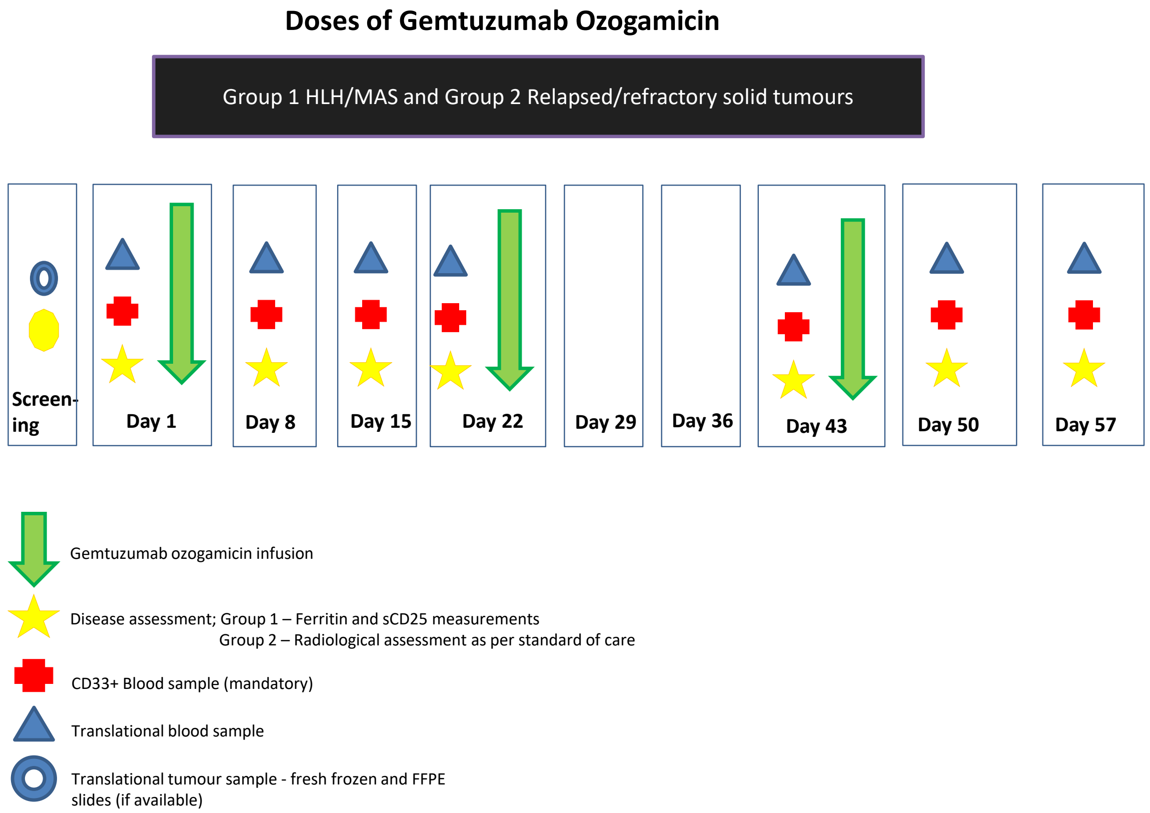

Figure 1: Trial schema

2 TIMING AND REPORTING OF INTERIM AND FINAL ANALYSES

The trial is expected to recruit over two years. There is no formal interim statistical analysis embedded into the design but an interim analysis of the data will be presented to an independent Trial Steering Committee after 3 patients have received treatment and reach day 57 for them to review the safety and feasibility of the trial.

The main analysis of the primary outcome will be performed by group, once all patients in each group have reached the final sample collection time point (day 57) after final dose sample.

The main analysis of the secondary outcomes will be performed once each patient has been followed up for a minimum of 1 year after the end of trial treatment. A full end of trial report will be produced at this time.

3 RECRUITMENT AND RANDOMISATION

3.1 Recruitment

At the point of analysis the following data will be reported:

- Length of recruitment (date opened and closed for recruitment)
- Recruitment over time (monthly) both cumulative and monthly counts

- Recruitment by site

3.2 Randomisation

As there is only one 'arm' per disease group and these are independent, there is no randomisation in the trial.

3.3 Ineligible Patients

Ineligible patients are defined as those registered patients who are subsequently found to not meet the eligibility criteria of the trial. The number of ineligible patients and reasons for their ineligibility will be reported; a sensitivity analysis may be conducted and reported if the number of ineligible patients is substantial. Protocol deviations relating to treatment will be reported as part of treatment compliance (section 6).

4 DATA QUALITY

Patient data is collected using paper case report forms (CRFs) for screening data and electronic case report forms (eCRFs) for the rest of the trial. Data collected in this way will be stored on a trial database. The trial database will be checked for missing data and any logical discrepancies as defined according to the trial specific data validation plan which will be developed by both the trial statistician and the trial coordinator.

4.1 Return Rate for each CRF

The proportion of returned CRFs compared to those that were expected will be reported. Missing critical data items will be highlighted (or at least acknowledged as queried).

4.2 Length of Patient Follow-Up

The number of patients lost to follow up will be reported as a proportion of all patients entered into the trial, along with the median length of follow up for patients. This will be reported for both disease groups separately and for the while trial.

5 TRIAL POPULATION

5.1 Baseline Patient Characteristics

Patient baseline characteristics will be tabulated; counts (%) and relevant summary statistics e.g. median and range, will be provided for categorical and continuous variables respectively. This information will be produced in separate tables for each disease group.

5.2 Definition of Populations for Analysis

Evaluable population will include all patients who received at least one dose of treatment and provide samples on day 1 of treatment and at least one other time point.

6 TREATMENT RECEIVED

A summary of the protocol treatment received over the treatment period will be reported. No significance testing will be carried out. Analyses will report the following:

- The proportion of patients who received treatment in total will be reported
- The number of patients receiving treatment in total
- The number of patients who did not start treatment, including the reason(s) for not starting treatment

- The number of patients discontinuing treatment and the reason(s) for discontinuation
- The number of doses missed and the reason(s)
- The number of treatment delays and the reason(s) for these delays
- Summary statistics for the time from registration to first treatment

7 ADVERSE EVENT REPORTING

The number of serious adverse events (including SARs), and the number of treatment-related deaths will be reported. The reporting period for SAE's will commence from the date of consent. Safety will be assessed by looking at grade 3 and 4 adverse events (CTCAE v4.0) as defined in the protocol.

The following details will be reported for each group:

- Max grade experienced for all patients.
- A summary of number of events and patients for all toxicities by event and grade.
- All serious adverse events will be reported, details to be presented include; admitting event, other events, reason for SAE, outcome, sequel and relatedness.

8 ANALYSIS

8.1 Definition and Calculation of Outcome Measures and Descriptive Analyses

8.1.1 Primary Objective(s)

Estimands will be used to address the question posed by the trial objective which is to assess the activity of gemtuzumab ozogamicin in the change in CD33+ myeloid cells in 2 parallel groups.

Change in CD33+

Population: All patients who receive at least one dose and have blood samples collected at initial screening or day 1 of treatment and at least one other future time point(day 8, 15, 22, 43, 50, 57). All evaluable patients.

Treatment: GO infusions at days 1, 22 and 43.

Outcome measure: CD33+ myeloid cell count from the blood sample collected on day 1 and at least one other future time point (day 8, 15, 22, 43, 50, or 57).

Intercurrent events: Blood samples may be collected at the wrong time or not at all. Blood samples could be collected at unscheduled time points, however the various summary statistics we calculate should capture and present this information. On treatment days blood samples should be taken prior to receiving treatment, if this is violated the next appropriate sample will be used for the analysis of summary statistics.

Summary statistics:

- Mean/Median relative change in CD33+ count for each group
- Mean/Median greatest absolute change in CD33+ count for each group
- Mean/Median time to reach lowest value of CD33+ count for each group

Change in CD33+ cells in the blood of patients is measured from samples taken on days (1, 8, 15, 22, 43), and on days 50 and 57 (7 and 14 days after administering the final dose respectively). The level of CD33+ will be repeatedly measured for each patient before and after treatment. For each patient

this data will be tabulated and displayed graphically, visualising the change in CD33+ cells over time. Similarly, the mean CD33+ count at each time will also be plotted to show overall trend for both disease groups separately. In the scenario where the majority of patients (more than 5 in one group) receive their doses on non-specified treatment days (i.e. not day 22 or 43) a summary plot of the mean CD33+ count at each treatment day will be produced.

Relative change is defined as the final value of CD33+ count (ideally taken on day 57) minus initial value of CD33+ count (taken on day 1 of treatment) all divided by the initial value. Depending on the normality of the distribution of the relative changes, the mean or median will be calculated and reported with standard deviation and inter-quartile range (IQR) respectively. If patients do not have a final value at day 57 the next most recent value will be used. Sensitivity analyses will be conducted, removing patients who do not have a final value at day 57. This will be done for each group separately.

Greatest absolute change is defined as the largest absolute value difference in CD33+ count from day 1 of treatment to any other subsequent time point. So, for each patient, the value of CD33+ count from day 8 onwards will be subtracted from values at day 1 to identify the greatest absolute change. For each disease group this will be summarised using means or medians with standard deviation or IQR respectively dependent on the normality of the absolute changes.

Time to reach lowest value is defined as the number of days to the day the lowest value of CD33+ is measured. As measurements are taken on predefined days (1, 8, 15, 22, 43, 50, 57) the time to reach the lowest value is either (0, 7, 14, 21, 42, 49 or 56 days) for each patient. As such numbers and percentages of patients, in each disease group, who reach their lowest CD33+ value at each time point will be reported. It is also possible for patients to deviate from the treatment plan as such the mean/median time to the lowest value will also be calculated.

All summary statistics will be reported with 95% confidence intervals. If appropriate, a more efficient Bayesian repeated measures analysis will be performed using flexible mixed effect models accounting for subject correlation to assess changes over time in CD33+ count.

8.1.2 Secondary Outcome Measures(s)

Overall and Progression free survival time

Population: For overall survival time all evaluable patients. For progression free survival only evaluable patients in group 2.

Outcome measure: Time from date of entry into the trial to the date of event. Where for overall survival time the event is death and for progression free survival time the event is disease progression.

Intercurrent events: Patients discontinuing the study, lost to follow up or are still alive at the time of analysis will be censored at date last confirmed alive.

Summary statistic: Median survival time for each disease group separately. Overall and progression free survivor functions will be estimated using the method Kaplan and Meier with rates presented at 3,6,9 and 12 months along with median survival time.

Incidence of grade 3 and 4 adverse events

As detailed in section 7.

8.2 Sample Size Calculations

No statistical testing will take place as such there is no target sample size. A maximum of 10 patients per group will be recruited. If any Bayesian modelling is to be performed simulations will be run to determine effective sample sizes and the protocol and SAP will be updated accordingly.

8.3 Subgroup Analysis

Outcome measures will be evaluated in relation to the number of treatment doses received to identify any potential associations. If any further subgroups of interest come to light then this SAP will be updated accordingly.

9 STATISTICAL SOFTWARE

Statistical analyses will be carried out using relevant statistical software; SAS (9.4 or later), Stata (14 or later) or R (Version 3.5.0 or later) respectively.

10 STORAGE AND ARCHIVING

Data snapshots will be named according to the date at which the snapshot was taken and to give an indication of its intended use e.g. final analysis. All programming files and snapshots of data will be stored here: $S:\backslash Stats\backslash Shared\backslash Trials\ Work\backslash CCTT\backslash Gotham\backslash GothamAnalysis$

Appendix 3. MetaCyto-derived positivity thresholds for macrophage markers

Thresholds used to determine macrophage markers' positivity calculated on normalised primary tumour data.

CD Marker	Macrophage subset	Cut-off (log2+1)
CD68	Pan-macrophage	2·65425
CD64	M1	3·81936
CD163	M2	2·84192
CD206	M2	4·92359

Appendix 4. CD33 expression on monocyte-derived macrophages and HLA-DR on CD14+ and CD15+ cells in leukocytes from cancer patients

(A) CD33 expression on monocytes post-treatment with granulocyte macrophage-colony-stimulating factor (GM-CSF) or macrophage-colony-stimulating factor (M-CSF) is shown as assessed by flow cytometry. (B) HLA-DR expression was assessed by flow cytometry to compare differential expression on CD14+ and CD15+ cells. (C) Increased HLA-DR expression was observed on CD14+ cells following gemtuzumab ozogamicin treatment. Representative from a single patient are shown.

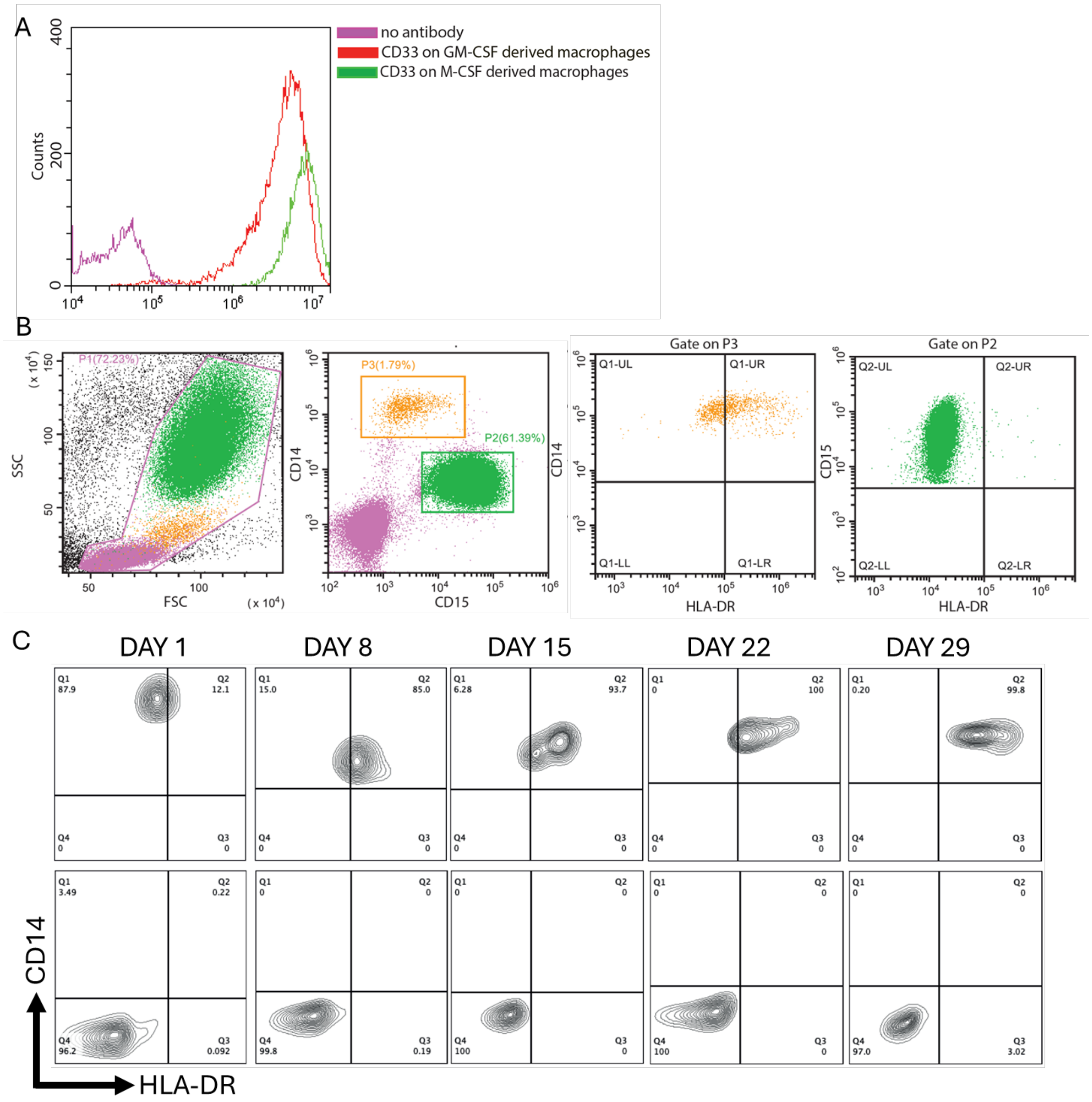

Appendix 5. Percentage change in CD33+ myeloid cell populations following gemtuzumab ozogamicin therapy

Blood samples collected from participants recruited into the GOTHAM trial at each visit (prior to gemtuzumab ozogamicin [GO] treatment were relevant) analysed for the percentage of CD33+ cells (A) and CD33+CD14+ cells (B) relative to baseline using flow cytometry. Each plot represents samples from a single patient; timing of GO administration (in days) is indicated above each plot. Note the prompt and robust drop in CD33+ cell count after cycle 1 and cycle 3 of GO given on the 3-weekly schedule in the final two plots of each panel. No blood draw was performed the week after GO cycle 2 on this schedule (day 29), hence the apparent lack of drop after cycle 2.

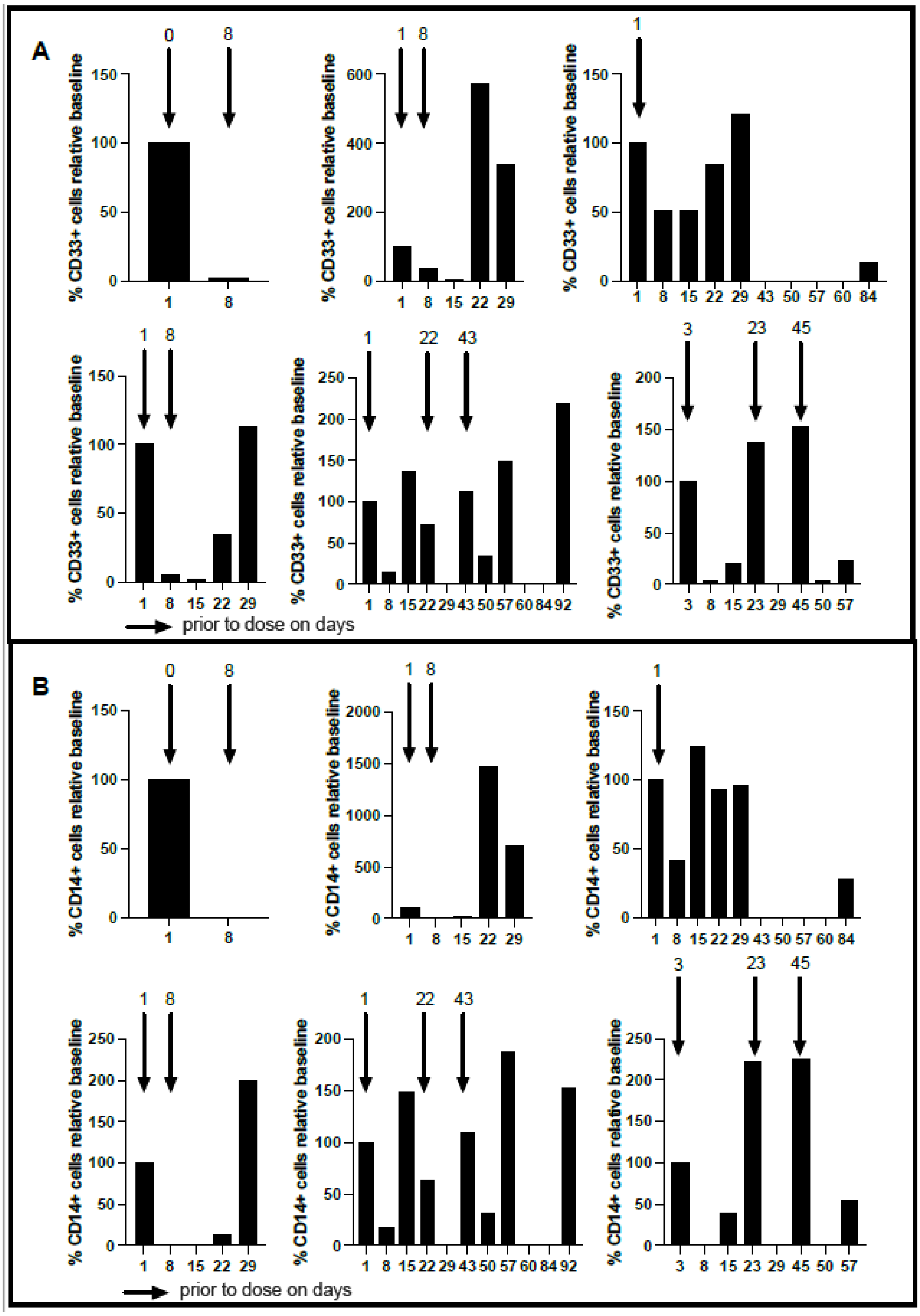

Appendix 6. Immune cell phenotype in leukocytes from cancer patient blood

The frequencies of CD19 (A), NK (B) and T reg (C) cells were quantified in leukocytes from cancer patients on the day prior to gemtuzumab ozogamicin administration. No effect on the frequency of the different cell population was observed following the treatment. Individual patients' samples are identified by a distinct colour on a separate plot.

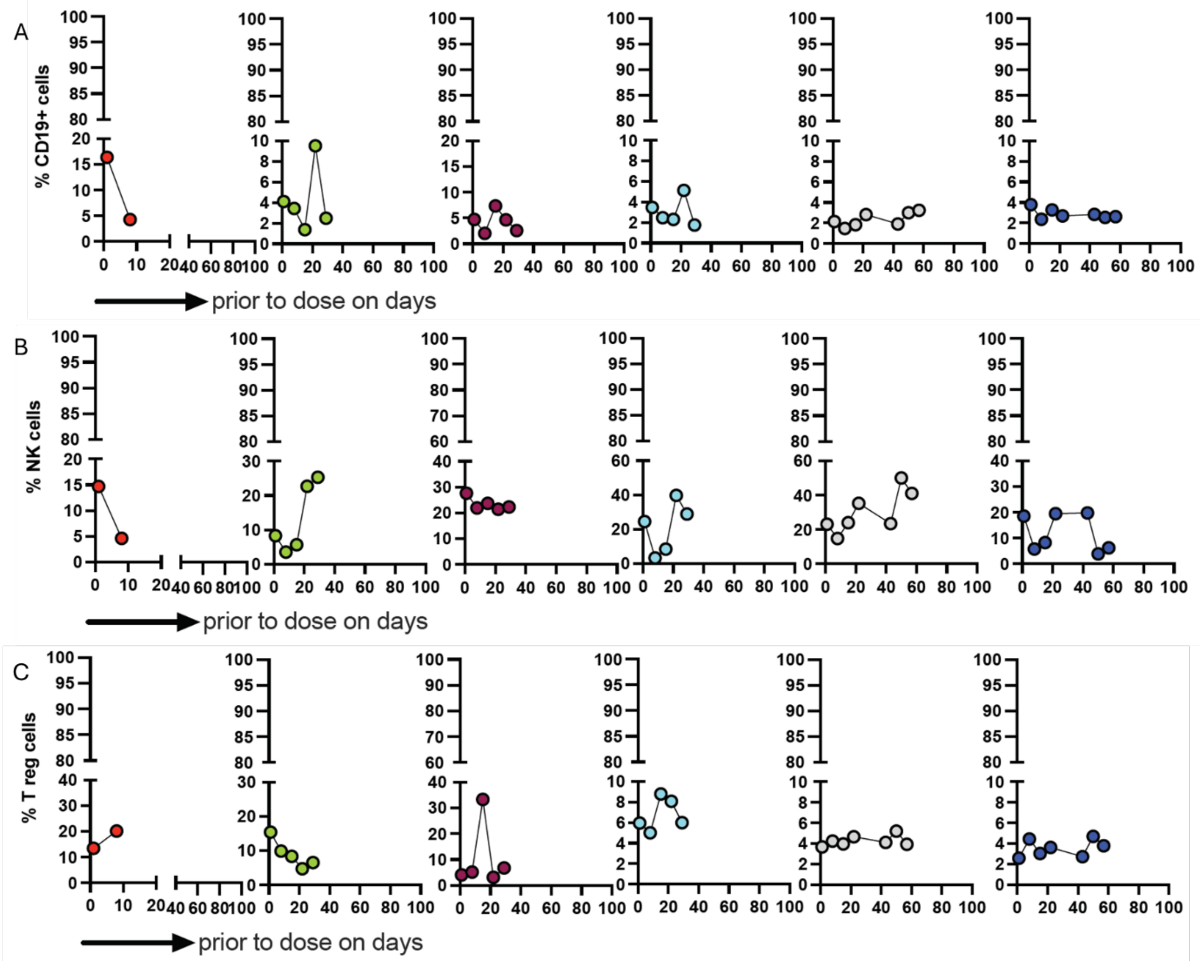

Appendix 7. Adverse events reported during GOTHAM

A list of (A) Grade 3 and 4 adverse events and (B) serious adverse events reported during GOTHAM.

Table A.

Toxicity	CTCAE Grade 3 (Number of Participants)	CTCAE Grade 4 (Number of Participants)	CTCAE Grade 5 (Number of Participants)
Abdominal pain	3 (2)	0	0
Anaemia	1 (1)	0	0
Back pain	1 (1)	0	0
Biliary tract infection	1 (1)	0	0
Dyspnoea	0	1 (1)	0
Fatigue	1 (1)	0	0
Febrile neutropenia	1 (1)	0	0
Infection – other 'Pneumonia'	0	0	1 (1)
Neutrophil count decreased	0	5 (4)	0
Respiratory failure	0	1 (1)	0
White blood cells decreased	0	1 (1)	0
Total	8 (3)	8 (4)	1 (1)

Table B.

Dose schedule	Relatedness	Days to Onset (Duration)	Reason	Admitting Toxicity (Grade)	Other Symptoms (Grade)
7-day	Definitely related	14 (4)	Hospitalisation	Febrile neutropenia (3)	-
7-day*	Possibly related	5 (14)	Hospitalisation	Abdominal pain (3)	Constipation (2); anorexia (2)
21-day**	Possibly related	8 (6)	Hospitalisation	Abdominal pain (3)	Anaemia (3); constipation (2); neutrophil count decreased (4)
21-day**	Possibly related	10 (4)	Hospitalisation	Pneumonia (5)	Dyspnoea (4), Respiratory failure (4)
7-day*	Unlikely to be related	25 (3)	Hospitalisation	Abdominal pain (3)	-

* & ** indicate events reported in the same participant.
